# How much of the forest sink is passive? Case of the United States

**DOI:** 10.1073/pnas.2513588123

**Published:** 2026-01-20

**Authors:** Eric C. Davis, Brent Sohngen, David J. Lewis

**Affiliations:** ^a^Market and Trade Economics Division, United States Department of Agriculture-Economic Research Service, Kansas City, MO 64105; ^b^Department of Agricultural, Environment, and Development Economics, The Ohio State University, Columbus, OH 43210; ^c^Department of Applied Economics, College of Agricultural Sciences, Oregon State University, Corvallis, OR 97331

**Keywords:** passive carbon, forest management, attribution, carbon fertilization, climate impact

## Abstract

Over time, carbon sequestered in temperate forests has increased, but the relative effects of passive and active drivers remain unclear. This study uses plot-level data to disentangle the contributions of six drivers (temperature, precipitation, CO_2_, management, age composition, and area) to these increases in 14 forest groups of the conterminous United States. From 2005 to 2022, the passive drivers (CO_2_, temperature, and precipitation) increased live tree carbon (C) by 66 teragrams (Tg) C y^−1^ with CO_2_ fertilization contributing most of the change. Among the anthropogenic drivers, declining forest area reduced live tree C by 31 Tg C y^−1^ while tree planting increased it by 23 Tg C y^−1^. Changes in age composition, driven by both passive traits and anthropogenic choices, increased live tree C by 89 Tg C y^−1^. By quantifying the share of removals attributable to passive uptake, this approach enables nations with national forest inventories to better utilize their forests to meet net-zero requirements.

## Significance

This study disentangles the impact of six active and passive drivers on forest C change in the conterminous United States. Separately identifying these drivers (temperature, precipitation, CO_2_, management, age composition, and area) enables more precise reporting of anthropogenic removals that are additional to passive uptake. From 2005 to 2022, the passive drivers (i.e., CO_2_, temperature, and precipitation) were responsible for 45% of the 148 Tg C y^−1^ increase precipitated by these six drivers.

**Fig. 1.**
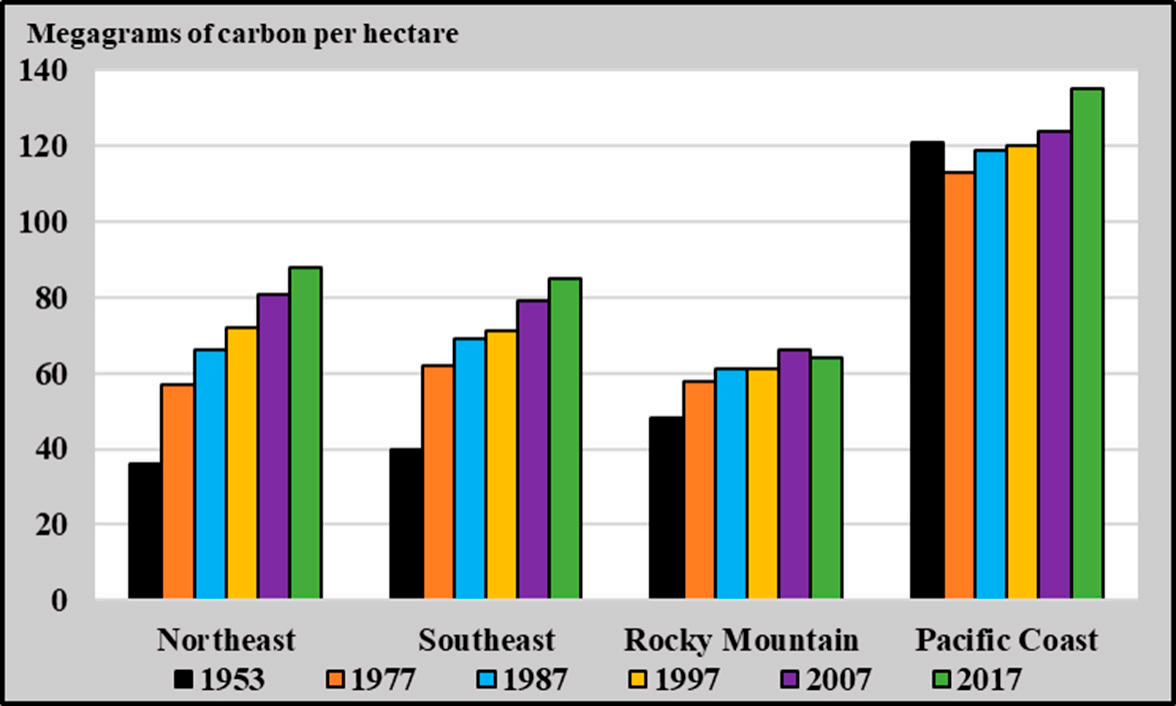
Change in carbon sequestration in the live biomass of forests of the conterminous United States from 1953 to 2017 by region. Northeastern states: CT/DE/IA/IL/IN/MA/MD/ME/MI/MN/MO/NH/NJ/NY/OH/PA/RI/VT/WV/WI. Southeastern states: AL/AR/FL/GA/KY/LA/MS/NC/OK/SC/TN/TX/VA. Rocky Mountain states: KS/NE/ND/SD/AZ/CO/ID/MT/NV/NM/UT/WY. Pacific Coast states: CA/OR/WA. Using data from the USFS Forest Resources of the United States (11), shown in *SI Appendix*, Fig. S2, and procedures established by Birdsey (12) and Smith et al. (13), the amount of carbon stored in live biomass, including in the coarse roots, is estimated.

The global terrestrial carbon sink has increased from 1.2 ± 0.4 petagrams (Pg) carbon (C) y^−1^ in the 1960s to 3.1 ± 0.6 Pg C y^−1^ in the 2010s (1). Across 2010–2019, the global net forest sink, including land-use changes, was 1.4 ± 0.7 Pg C y^−1^ (2) with forests in the United States responsible for 12% (3) or roughly 168 teragrams (Tg) C y^−1^. While forest succession (reforestation after agricultural use) was the largest contributor to the terrestrial carbon sink in the United States in the first half of the 20th century (4–10), its impact has lessened with forestland area increasing by just 3% since 1953 (11). Fig. 1 shows that, as increases in area slowed (*SI Appendix*, Fig. S1), carbon density (measured as live biomass density) grew significantly, driving most of the increase in the US net forest sink since the 1950s. Between 1953 and 2017 in the Northeast United States, the amount of carbon sequestered increased 144% (+52 Mg C ha^−1^), and in the Southeast, it increased 111% (+45 Mg C ha^−1^). Smaller changes occurred in the Rocky Mountain (+34%: +16 Mg C ha^−1^) and Pacific Coast regions (+12%: +14 Mg C ha^−1^), potentially due to natural disturbances like forest fires (11).

The drivers of increased carbon density include shifts in the average age of forests (14–18), changes in temperature and precipitation (9, 17, 19, 20), nitrogen deposition (21), CO_2_ fertilization (22–29), and forest management (30). The relative effect of each of these drivers though is unclear. Empirical studies have examined the effects of temperature and precipitation on forest biomass (17), the types of forests planted (31), and the profitability of forestland (32), but they did not assess the effects of CO_2_ fertilization and management. Other works, including the Free-air CO_2_ Enrichment (FACE) studies, have examined the impact of CO_2_ fertilization on forest growth or biomass (22–29), but they did not separately address temperature, precipitation, and management.

This study presents an approach to disentangle the contributions of six drivers (temperature, precipitation, CO_2_, management, age composition, and area) to the density changes illustrated in Fig. 1 as well as the overall US forest carbon flux in the conterminous United States. To accomplish this, we derive spatially and temporally explicit forest yield estimates that, in combination with observed forest inventory data from the 1960s to the present, enable identification of the impact of these drivers on aboveground US wood volume and the carbon stored in live biomass, including coarse roots. The results help illustrate the role of passive uptake (e.g., CO_2_ fertilization) relative to anthropogenic actions (e.g., forest management) (33–35) and enable countries with similar plot-based data to quantify the role of anthropogenic C in their Nationally Determined Contributions.

## Results

We begin by identifying the impact of shifts in temperature, precipitation, and CO_2_ on wood volume between 1960 and 2023. This period captures 76.5% of the increase in atmospheric CO_2_ concentration since 1850 (36–38)—as well as most of the forest inventory data available in the United States (39). Precipitation and temperature patterns in the conterminous US shifted considerably over this period (*SI Appendix*, Figs. S3 and S4). Average precipitation increased in all seasons except fall with the largest increases concentrated in the East and decreases more prevalent in the West. The largest precipitation increase occurred in winter (+15.4 mm). Average temperatures also increased each season, with the biggest increase in winter (+0.7 °C).

This first analysis uses the data for all available USFS-FIA inventories (39) for 10 forest groups: 1) Aspen/Birch, 2) Elm/Ash/Cottonwood, 3) Loblolly/Shortleaf Pine, 4) Maple/Beech/Birch, 5) Oak/Gum/Cypress, 6) Oak/Hickory, 7) Oak/Pine, 8) Slash/Shortleaf Pine, 9) Spruce/Fir, and 10) White/Red/Jack Pine. By combining data across species and time, we capture the substantial variation in climate (and other variables) within the US forest population, allowing estimation of the drivers of wood volume with generalizability. We include only naturally regenerated (unmanaged) stands in this cross-type analysis to avoid identification issues related to technological improvements in planted stands. In the regression results (*SI Appendix*, Table S1: Column 1), the parameter for CO_2_ (*P* < 0.01) is significant, and *F*-tests on the cubic polynomial for each seasonal climate variable are all significant (*P* < 0.01).

**Fig. 2.**
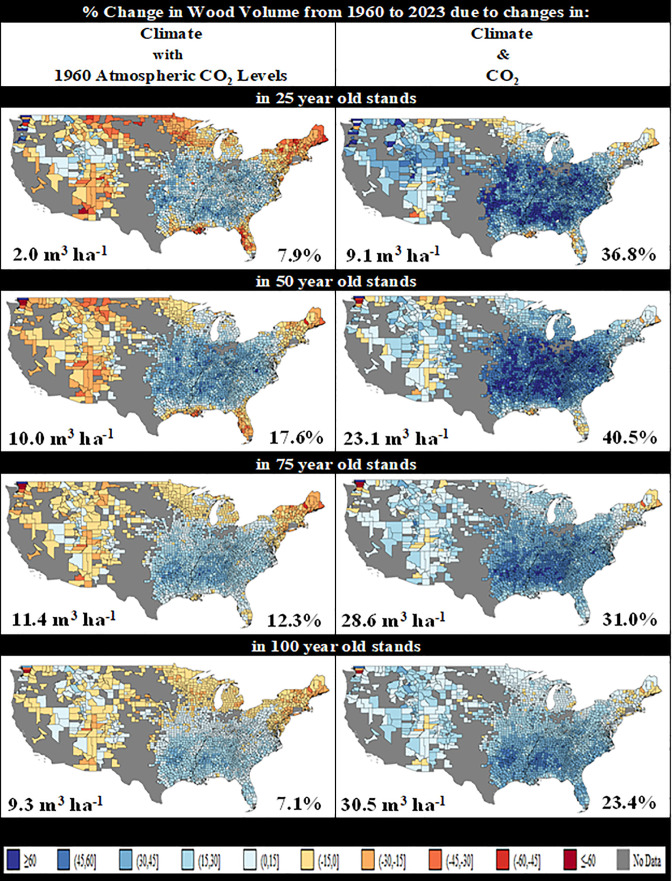
Estimated impact of changes in climate and elevated CO_2_ on wood volume from 1960 to 2023, by county and age, based on the combined observations of naturally regenerated stands aged 1 to 150 of 10 forest groups. Note: The numerical values in the bottom left of each pane detail the mean impact and the values in the bottom right of each pane detail the mean percentage change. Forest groups: Aspen/Birch, Elm/Ash/Cottonwood, Oak/Gum/Cypress, Oak/Hickory, Oak/Pine, Loblolly/Shortleaf, Maple/Beech/Birch, Slash/Shortleaf, Spruce/Fir, and White/Red/Jack.

We use these results to calculate how changes in temperature, precipitation, and CO_2_ affected volume between 1960 and 2023 for each county where at least 1 of our 10 forest groups was observed (39). Our estimates suggest that, due to changes in climate alone, a 25-y-old stand in 2023 had 7.9% more volume than a comparable 25-y-old stand did in 1960. Similarly, a 100-y-old stand had 7.1% more volume (Fig. 2: Column 1). Changes in precipitation increased volume in most of the East (*SI Appendix*, Fig. S5: Column 1) with an average impact on 25-y-old stands of +8.7% (+2.2 m^3^ ha^−1^). Changing temperatures decreased volume by 0.8% (−0.2 m^3^ ha^−1^) on average in 25-y-old stands, with the strongest impacts in the Northeast, upper Midwest, and West (*SI Appendix*, Fig. S5: Column 2). While the climate variables had mixed impacts, elevated CO_2_ had a uniformly positive impact on volume, ranging from +16.2% for 100-y-old stands to +28.9% for 25-y-old stands. The combined impacts of temperature, precipitation, and CO_2_ were positive, increasing average volume 36.8% (9.1 m^3^ ha^−1^) for 25-y-old stands and 23.4% (30.5 m^3^ ha^−1^) for 100-y-old stands (Fig. 2: Column 2).

We are unable to assess whether there is an attenuation of CO_2_ or climate impacts. The smaller percentage-change impact reported for older stands likely reflects the degree of overlap between the compared periods. A stand that was 100 y old in 2023 was exposed to the same environmental conditions as a stand that was 100 y old in 1960 for 37 y, ceteris paribus. For 25- and 50-y-old stands, there was no overlap but instead gaps of 39 and 14 y, respectively.

To assess the climate sensitivity of each forest group (i.e., differences in parameter estimates on climate and CO_2_ drivers), our second analysis estimates separate regression equations for the forest groups (*SI Appendix*, Tables S2 and S3) and finds that the parameters on the CO_2_ variable and the *F*-tests on the cubic polynomial for each seasonal climate variable are significant for all forest groups (*P* < 0.01). Using these results, we predict the change in stand volume for each forest group from 1960 to 2023 (*SI Appendix*, Figs. S6–S25). Changes in temperature and precipitation resulted in increased volume in 25-y-old stands for all forest groups except Spruce/Fir (−65.2%), White/Red/Jack (−4.0%), and Elm/Ash/Cottonwood (−1.5%). Elevated CO_2_ had a positive (*P* < 0.01) effect on volume for all 10 forest groups, with impacts on 25-y-old stands ranging from +25.7% for Loblolly/Shortleaf to +33.1% for Aspen/Birch. In most counties and age groups, the impact of elevated CO_2_ offset any negative impacts from changes in climate (Column 2 of *SI Appendix*, Figs. S6–S15). The exception is Spruce/Fir, where the combined impact was negative and volume declined 36.4% (15.5 m^3^ ha^−1^) in 25-y-old stands and 6.2% (13.5 m^3^ ha^−1^) in 100-y-old stands. Robustness checks in our third analysis confirm these results using alternate specifications (*SI Appendix*, Tables S4–S7).

To assess the impact of management, the fourth analysis focuses on the act of planting trees as an observable proxy for a variety of management actions that are more likely to occur on planted stands than naturally regenerated ones. These include seed selection, initial density selection, and vegetation management actions like precommercial thinning and prescribed burning. For this analysis, the three softwood groups with considerable historical planting and management effort are examined (*SI Appendix*, Table S8). The impact of planting on volume is identified by estimating the difference in volume in 2023 between naturally regenerated and planted stands using observations of forests up to 50 y of age, aligning with typical rotational forestry practices (40–45) and data in our study (*SI Appendix*, Fig. S26). At 10 y of age, two-sided *t*-test results indicate that planting did not affect volume (*P* < 0.01) (*SI Appendix*, Table S9). This, we believe, is due to management activities like competition suppression that foster the growth of selected trees but take a temporary toll on volume. At and above 20 y of age, planting is shown to have a significant (*P* < 0.01), positive impact on volume. By 40 y of age, planted Loblolly/Shortleaf stands had a volume that was +27.7% (+98.6 m^3^ ha^−1^) larger than naturally regenerated stands while planted Slash/Longleaf stands had +33.0% more volume (+57.6 m^3^ ha^−1^) than naturally regenerated stands. Planting increased White/Red/Jack volume by +25.3% (+77.3 m^3^ ha^−1^).

To identify the relative impacts of changes in climate, CO_2_, and planting as well as changes in age composition and area, in our fifth analysis, we focus on specific periods in which robust information on the age-class, area, and volume distributions of all forest groups was collected. The inventory data allow us to disentangle these effects for 12 southern states over two periods: 1974–2005 and 2005–2022 (*SI Appendix*, Tables S10–S12). We also adopt a region-specific yield function for this analysis (*SI Appendix*, Table S13: Column 1).

**Fig. 3.**
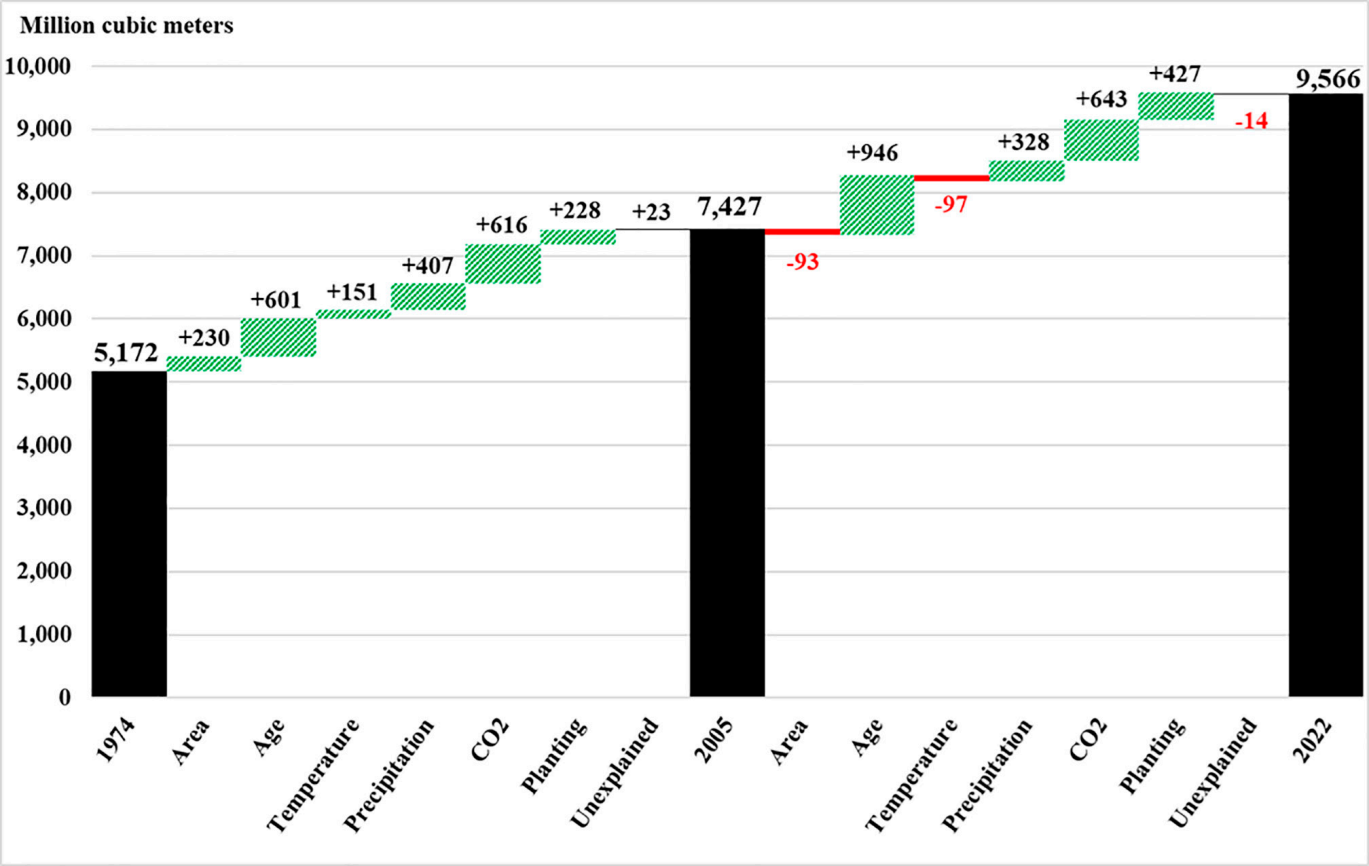
Estimated impact of key drivers (area/age/temperature/precipitation/CO_2_/planting) on change in wood volume in 12 southern states from 1974 to 2022, based on the combined observations of eight forest groups. For the South (states: AL/AR/FL/GA/LA/MS/NC/OK/SC/TN/ TX/VA), USFS-FIA inventories closest to 1974 (range: 1968–1980), 2005 (range: 2004–2010), and 2022 (range: 2020–2022) were used. The area column shows the impact of the change in the range. The age column shows the impact of changes in age composition across the ages studied which is 1 to 100 y. The planting column details the gains derived from planting across all ages of planted stands, relative to naturally regenerated stands, in the end-of-period years (2005 and 2022). The unexplained column shows the gap between our estimates and the actual change that the USFS recorded for these forest groups.

For the early period (1974–2005), we calculate that the six drivers generated a 2,232 million m^3^ increase in aboveground volume (Fig. 3), implying an additional 1,771 Tg C stored in live biomass, including coarse roots. Of that total, elevated CO_2_ (+616 million m^3^: +489 Tg C), changes in age composition (+601 million m^3^: +477 Tg C), and changes in precipitation (+407 million m^3^: +323 Tg C) had the largest aggregate effects. Increased forest area (+230 million m^3^: +183 Tg C), planting (+228 million m^3^: +181 Tg C), and temperature (+151 million m^3^: +120 Tg C) also had positive impacts.

The latter period (2005–2022) covered 14 fewer years, but the aggregate change from the six drivers was similar (+2,153 million m^3^: +1,708 Tg C) (Fig. 3). Drivers with the largest effect were age composition (+946 million m^3^: +751 Tg C), elevated CO_2_ (+643 million m^3^: +510 Tg C), planting (+427 million m^3^: +339 Tg C), and precipitation (+328 million m^3^: +260 Tg C). Relative to the early period, elevated CO_2_ and planting had larger impacts in the latter period, +4.4% and +87.0%, respectively, while the impact of precipitation was smaller and the impacts of temperature and area change turned negative.

In our sixth analysis, we present the results by forest group (*SI Appendix*, Tables S2, S3, and S14), which illustrate that in the South from 1974 to 2022 a reduction in area drove volume losses in Oak/Pine (−332 million m^3^), Oak/Gum/Cypress (−128 million m^3^), Oak/Hickory (−78 million m^3^), and Slash/Longleaf (−34 million m^3^) (*SI Appendix*, Figs. S27 and S28). The losses for Oak/Pine and Slash/Longleaf likely resulted from land conversion to planted Loblolly/Shortleaf, whose volume grew by 644 million m^3^ due to increased area. The combined impact of temperature and precipitation change was positive for all forest groups from 1974 to 2005. After 2005, climate impacts were smaller for all forest groups, except for Loblolly/Shortleaf, and negative for three forest groups: Oak/Gum/Cypress (−70 million m^3^), Oak/Hickory (−13 million m^3^), and Elm/Ash/Cottonwood (−12 million m^3^). The impact of elevated CO_2_ was positive for all forest groups, with the magnitude of the impact being larger in the latter period for four of the eight forest groups, most notably for Loblolly/Shortleaf (+87 million m^3^) and Oak/Hickory (+26 million m^3^). The impact of planting was positive for all three managed forest groups in both periods but larger in the latter period. Planting was the second largest driver of Loblolly/Shortleaf volume growth.

**Fig. 4.**
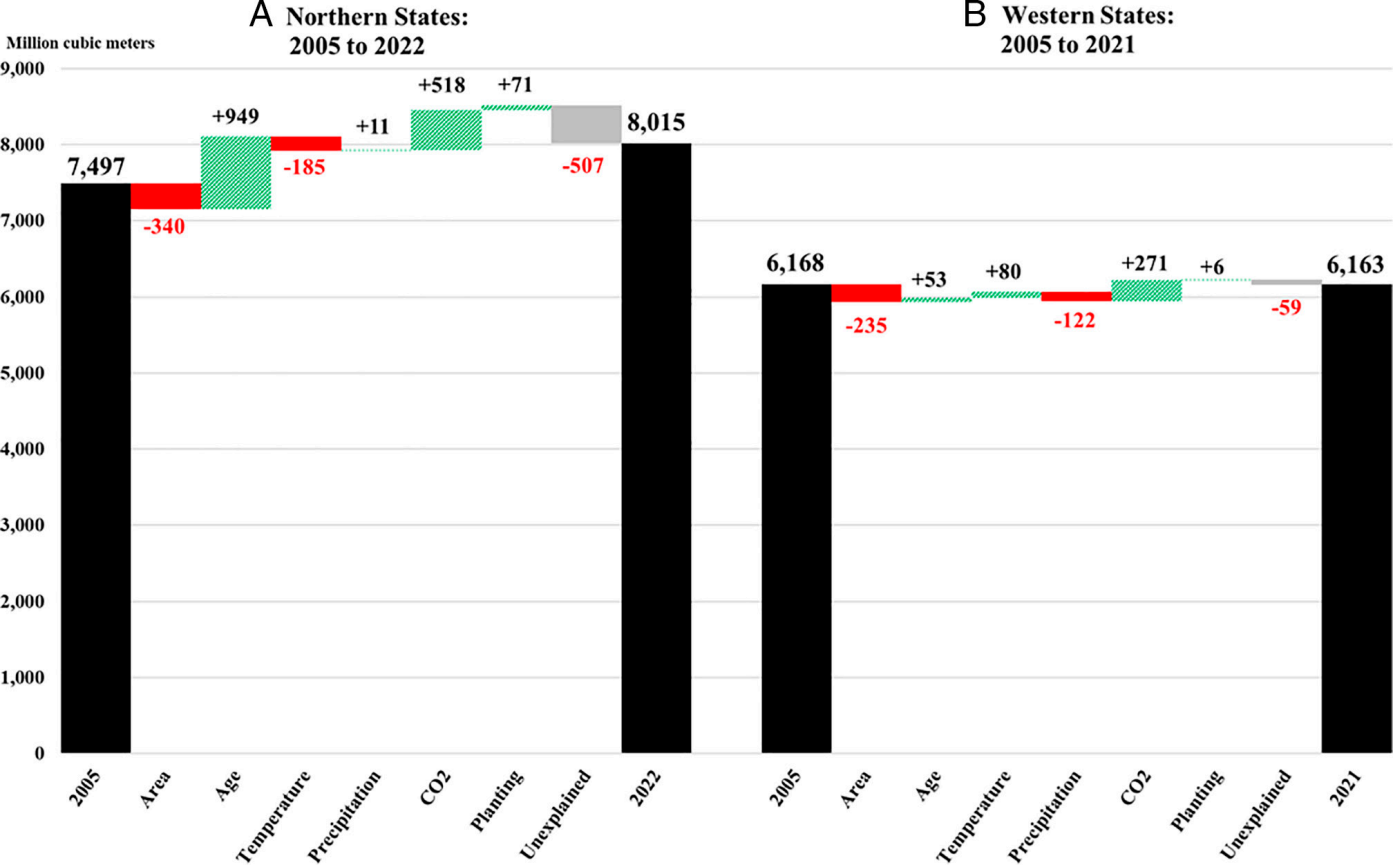
Estimated impact of key drivers (area/age/temperature/precipitation/CO_2_/planting) on change in wood volume: (*A*) in 21 northern states from 2005 to 2022, based on the combined observations of nine forest groups and (*B*) in 15 western states from 2005 to 2021, based on the combined observations of four forest groups. For the North (CT/DE/IA/IL/IN/KY/MA/MD/ME/MI/MN/MO/NH/NJ/NY/OH/PA/RI/VT/WI/WV), the inventories from 2005 and closest to 2022 (range: 2020-2022) are used. For the West (AZ/CA/CO/ID/KS/MT/ND/NE/NM/NV/OR/SD/UT/WA/WY), the inventories closest to 2005 (range: 1999 to 2011) and 2021 (range: 2020 to 2022) are used. The area column shows the impact of the change in the range. The age column shows the impact of changes in age composition across the ages studied which is 1 to 100 y for the northern states and 1 to 200 y for the western states. The planting column details the gains derived from planting in the end-of-period year. The unexplained column shows the gap between our estimates and the actual change that the USFS recorded.

In our seventh analysis, the 21 northern states were analyzed starting in 2005 as inventory data for prior years were not sufficiently robust to identify shifts in area and age-class distribution (*SI Appendix*, Tables S13: Column 2, *SI Appendix*, Tables S15 and S16). The six drivers generated a 1,025 million m^3^ increase in volume (+786 Tg C) from 2005 to 2022 (Fig. 4*A*). Changes in age composition, elevated CO_2_, and precipitation increased volume the most while planting had a modest positive effect. Volume fell as a result of declining forest area and higher temperatures. When decomposed by forest type, area losses happened in seven of the nine forest groups (*SI Appendix*, Fig. S29). Elevated CO_2_ and continuing increases in age exerted a positive impact on all forest groups. The combined effect of changes in temperature and precipitation was negative for all forest groups except Oak/Pine and Loblolly/Shortleaf. Planting had a positive impact on volume for both Loblolly/Shortleaf and White/Red/Jack.

For 15 western states (*SI Appendix*, Table S13: Column 3, *SI Appendix*, Tables S17 and S18), we examine four forest groups (Douglas-Fir, Ponderosa Pine, Lodgepole Pine, and Fir/Spruce) that constitute considerable volume in the region and have the most robust data over the period 2005–2021. The six drivers generated a 54 million m^3^ volume increase (Fig. 4*B*) or 27 Tg C. Elevated CO_2_ had the largest positive influence while changes in temperature, age composition, and planting had smaller, but still positive effects on volume. A decline in forest area caused volume to fall, as did changes in precipitation. The changes from 2005 to 2021 by forest group are presented in *SI Appendix*, Fig. S30.

## Discussion

In the South, forest planting and an expanded forest area increased volume by an average of 15 million m^3^ y^−1^ from 1974 to 2005 and by 20 million m^3^ y^−1^ from 2005 to 2022 (Fig. 3). Between 1974 and 2005, forest area increased due to economic factors that favored land-use changes from agriculture to forests (46). After 2005, however, there was a moderate loss in forest area, driven by continued urban development combined with changes in the relative profitability of cropland and forestland (47). The impact of forest management (planting) was 87.0% larger in the latter period than the early period, providing a volume boost that largely offset the reduction in volume due to the losses in forest area. Changes in age class contributed 19 million m^3^ y^−1^, on average, to the increase in volume in the early period and 56 million m^3^ y^−1^ in the latter period. The passive drivers (temperature, precipitation, and CO_2_) had a stronger impact on volume in the latter period (+51 million m^3^ y^−1^) than in the early period (+37.9 million m^3^ y^−1^). The contribution of CO_2_ fertilization strengthened from 20 million m^3^ y^−1^ in the early period to 38 million m^3^ y^−1^ in the latter period, and the impact of precipitation increased from 13 to 19 million m^3^ y^−1^. The impact of temperature, however, turned negative and decreased volume by an average of 6 million m^3^ y^−1^ in the latter period.

Outside of the South, changes in temperature and precipitation have had a negative impact for many forest groups. Spruce/Fir has seen the negative effects of changes in climate outweigh the gains conferred by elevated CO_2_ (*SI Appendix*, Figs. S7, S17, and S29). In the long-term, Spruce/Fir likely needs to migrate, either naturally (48, 49) or via planting, to have a long-term future in the United States, although our mapped results suggest that suitable habitat for Spruce/Fir is declining (*SI Appendix*, Figs. S7 and S17). This issue of changes in climate prompting shifts in habitat is not limited to Spruce/Fir. Warmer temperatures in the North are expected to enable many traditionally southern forest groups, like Loblolly/Shortleaf, to expand their ranges northward via anthropogenic planting in eastern US forests (50).

More broadly, across the conterminous United States where at least one of the 14 forest groups used in this study was observed, the six drivers, from 2005 to 2022,^*^ increased wood volume by 3,232 million m^3^ (+148 Tg C y^-1^), from 21,091 to 24,323 million m^3^ (Figs. 3 and 4). Of this increase, the passive drivers contributed 45% (+1,447 million m^3^ or +66 Tg C y^−1^). Potentially via increased water use efficiency (51, 52), CO_2_ fertilization contributed the most (+1,432 million m^3^). For the climate variables, gains due to increases in precipitation were counterbalanced by the effects of higher temperatures, but the impacts were not uniform. Lower average precipitation, for example, reduced volume in the West while higher average precipitation increased volumes in the North and South.

For the anthropogenic drivers, planting (management) increased volume by 504 million m^3^ (+23 Tg C y^−1^) with 84.7% of the gain happening in southern forests. Declining forest area reduced volume by 668 million m^3^ (−31 Tg C y^−1^). Changes in age composition had the largest impact across all drivers, increasing volume by 1,949 million m^3^ (+89 Tg C y^−1^) due to forests becoming older. Whether to classify this age-related volume change as passive uptake or anthropogenic removal merits careful consideration. Some observers consider carbon sequestration resulting from reforestation and aging as passive uptake (34). When, however, there is significant private ownership of timberland, as there is the United States, owners determine how their land is used (46) and when (or if) the forests are cut (53). Their decisions affect stand ages and carbon storage. For example, during and after the 2008 housing crisis, economic conditions led owners to reduce wood harvesting, which increased the average age of stands and CO_2_ uptake. Thus, changes in age composition driven by the decisions of private landowners can be considered anthropogenic removal.

There are other factors that affect volume that cannot be identified in this study. These unexplained factors are behind the difference between our estimates and those reported by the USFS (Figs. 3 and 4). One such factor is nitrogen deposition. Although we control for nitrogen deposition in our estimates through time-varying fixed effects, we cannot cleanly identify its effect in our aggregate estimates. Other factors include invasive plants (54), insects like the Emerald Ash Borer (*Agrilus planipennis Fairmaire*) (55), ice storms and wind damage (including hurricanes) (56), and wildfire disturbances, which are growing in geographical scale and intensity in the West (57–59) and can lead to losses in forest area through a lack of tree regeneration (60). Although we are unable to separately identify these impacts, we estimate that from 2005 to 2022, on aggregate, they reduced volume by 580 million m^3^ with the largest impacts in the northern United States (−507 million m^3^).

## Methods

This study disentangles the impact of six key drivers of forest carbon flux in US forests. Changes in age composition and forest area are captured directly using inventory data. Temperature and precipitation are identified by linking wood volume in forest plots to climate data, which varies spatially and temporally (61). Atmospheric CO_2_ concentration is identified using methods described in ref. 25. Forest management is estimated by examining differences between naturally regenerated and planted stands of the same forest group. Detailed information about the *Methods and Materials* is provided in *SI Appendix* file (62).

## Supplementary Material

Appendix 01 (PDF)

## Data Availability

The data and code we developed for this analysis from the primary sources are available at https://github.com/bsohngen/Davis_etal (62).

## Footnotes

^*^This calculation uses the increase from 2005 to 2021 for the U.S. West.
