# Supplementary material for: How much of the forest sink is passive? Case of the United States
Source: Proc Natl Acad Sci U S A. 2026 Jan 20;123(4):e2513588123. doi: 10.1073/pnas.2513588123 (PMC12846823; doi:10.1073/pnas.2513588123)
Supplement: Supplementary file 1 — Appendix 01 (PDF) [file pnas.2513588123.sapp.pdf]

# Supporting Information for
# How Much of the Forest Sink Is Passive? Case of the United States

Eric C. Davis, Brent Sohngen, & David J. Lewis

Corresponding author: Brent Sohngen
Email: Sohngen.1@osu.edu

**This PDF file includes:**

Materials and Methods
Figures S1 to S32
Tables S1 to S20
SI References

## Materials and Methods

In this study, the impact of six key drivers (forest management/age composition/area/ temperature/precipitation/$CO_2$ fertilization) on aboveground wood volume is disentangled using historical data of U.S. forests.

### Materials

Data from the U.S. Forest Service's Forest Inventory and Analysis (USFS-FIA) Program *(1)* were used for the calculations of aboveground wood volume per hectare as well as for many controls regarding the physical environment of the stands. For example, controls were included for elevation, land ownership, and the presence or absence of fire events or other such disturbances. Ten forest groups were used throughout our analysis: 1) Elm/Ash/Cottonwood, 2) Oak/Pine, 3) Oak/Hickory, 4) Maple/Beech/Birch, 5) White/Red/Jack Pine, 6) Aspen/Birch, 7) Slash/Shortleaf Pine, 8) Oak/Gum/Cypress, 9) Loblolly/Shortleaf Pine, and 10) Spruce/Fir. Observations of trees aged 1 to 150 within the 48 conterminous states over the years from 1968 to 2023 were included (Figs. S26 & S31). Four other forest groups (Douglas-Fir, Ponderosa pine, Lodgepole pine, and Fir/Spruce) were used in only one analysis, as limited observations were available (years 2005 to 2023). For these forest groups, ages ranged from 1 to 200 years (Fig. S32).

Data from the PRISM Climate Group *(2)* were used for temperature and precipitation controls. As in our study we have stands up to 200 years of age and as we wanted to understand the effect of the climate conditions under which they grew by comparing stands observed in 1960 to those observed in 2023, we ideally would have had climate data going back as far as 1761. However, the PRISM data only goes back to 1895. To overcome this data availability issue, we took an average of the climate conditions that existed in each county between 1895 and

1914. As anthropogenic climate impacts are known to have been low in those years, using this twenty-year average to approximate the conditions that existed in each of the missing years seemed reasonable. Using an average also reduced the risk of potentially basing missing year values on an outlier year, which might have occurred had we chosen only one year upon which to base our calculations for the missing years. With the needed climate data established, we then calculated the lifetime average seasonal temperature and precipitation for each stand, by age and location. The seasons were defined as follows: Winter (December through February), Spring (March through May), Summer (June through August), and Autumn (September through November).

Methods

*Analysis 1: Aggregate impact of $CO_2$ and climate change on forest yield*

- Table S1, Fig. S5, and Main Text Fig. 2

To disentangle the impact of the six drivers under study (area/age composition/forest management/temperature/precipitation/$CO_2$ fertilization) on wood volume, we constructed an exponential tree volume function to model changes in aboveground wood volume across the yield curve. The generalized form is presented in Eq. (1).

$$\text{Ln(volume/hectare)} = \alpha + \beta 0 * 1/\text{Age} + \beta j * Xj + \varepsilon \quad (1)$$

Climate variables that detail the average lifetime precipitation and temperature that each stand experienced entered our model in polynomial form. For climate effects, there is spatial variation, and thus we were able to use the lifetime seasonal average temperature and precipitation each plot experienced, as all forest plots in the dataset had experienced different lifetime temperature and precipitation exposures due to this variation in climate outcomes at any

point in time and over time. In most of our models, the climate variables were interacted with either age or latitude. Interacting the variables with latitude was considered important as some research now suggests that, while for many years there has been a relatively stable optimal geographic location for each forest group, changes in climate will lead to forests migrating northward *(3-4)*. Others though feel that factors like photoperiod may make such migration less successful *(5)*, as its relationship with the emerging climate may create dramatically different results *(6)*. What is sure is that photoperiod has an interactive effect with climate that influences important events, such as the onset of the growing season *(7-9)*, and thus we accounted for this in our model by interacting our climate variables with latitude, which is a proxy for photoperiod. The interaction captures how the effects of changes in temperature and precipitation strengthen or weaken moving from south to north with a positive parameter indicating that an increase in the seasonal temperature or precipitation variable had a stronger effect further north.

The variable that was used to assess the impact of elevated $CO_2$ was constructed as the logged lifetime cumulative exposure of each stand to atmospheric $CO_2$. That is, to capture the effect of elevated $CO_2$, the age- and time-specific exposure to $CO_2$ each plot experienced over its lifetime was utilized. For example, for a stand that was two years old in 1960, the lifetime $CO_2$ exposure was calculated as the sum of the 1959 value of 316.0 ppm plus the 1960 value of 316.9 ppm for a total lifetime exposure of 632.9 ppm. This was important, as over the years that trees in our study lived (1761 to 2023), atmospheric $CO_2$ has increased from around 280 ppm to more than 420 ppm *(10-12)*. While the level of $CO_2$ in the atmosphere does not vary spatially, by summing the yearly concentrations of atmospheric $CO_2$ over a period equal to the age of each stand in the study, heterogeneity was introduced through different time-period and age-class combinations. To address potential confounding factors that also are correlated with time (e.g. nitrogen deposition and large-scale disturbances associated with invasive species like the

Emerald Ash Borer, *Agrilus planipennis Fairmaire*), time dummy variables (fixed effects) and controls for evidence of disturbances were employed in all regressions.

The impact of planting (forest management) was captured with a dummy variable that indicates the regeneration method, and this variable was interacted with our age variable. This enabled us to quantify the difference in wood volume between naturally regenerated and planted stands. Other $\beta_j$ coefficients show the impact of a range of variables that have been shown to have a strong connection with growth patterns *(13-14)*. These include things like water availability and soil quality. The stocking condition on the land was also controlled, as changes in stocking could allow an increased number of trees to be misinterpreted as an average tree having an increased wood volume. Information on these salient site- and time-specific covariates is provided in Table S20. U.S. Federal Information Processing Standards (FIPS) code dummy variables were used to control for the unobserved, time-invariant characteristics of counties. The $\beta_0$ coefficient details the impact of additional years of life on wood volume. The unidentified noise in the model is represented by $\varepsilon$. To transform our results into a volume in cubic meters per hectare, we used the nonparametric smearing estimate method *(15)*. To provide an estimate of the mass of carbon, in grams, stored in the forest groups we study, we followed established precedent in converting from wood volume *(16-17)*. These conversion factors allowed us to provide an estimate of the amount of carbon stored in the live biomass of these forest groups, including in the coarse roots. The amount of carbon stored in deadwood and litter was not considered when making our estimates.

Using data from the PRISM Climate Group *(2)*, visual representations of the changes in precipitation and temperature over the period from 1960 to 2023 were created (Figs. S3 & S4). The input values for both 1960 and 2023 are 5-yr averages to minimize the possibility of anomalous weather driving the results. That is, 1960 precipitation was calculated as the average

of the seasonal precipitation in the years from 1956 to 1960.

To identify how these changes impacted wood volume over this period on a county-by-county basis, data were first limited to observations of naturally regenerated plots aged 1 to 150 of the ten forest groups in our study. This was done to ascertain the effect of changes in climate and elevated $CO_2$ absent worries about things such as changing seed stocks that could confound their identification. Our model was formulated as shown in Eq. (2).

$$\begin{aligned}\text{Ln(volume/hectare)} = \alpha + \beta0^*\text{Ln(Lifetime } CO_2) + \beta1^*1/\text{Age} + \beta2^*\text{Site Class} + \\ \beta3^*\text{Seasonal Temperature} + \beta4^*\text{Seasonal Temperature}^2 + \beta5^*\text{Seasonal Temperature}^3 + \\ \beta6^*\text{Seasonal Precipitation} + \beta7^*\text{Seasonal Precipitation}^2 + \beta8^*\text{Seasonal Precipitation}^3 + \\ \beta9^*\text{Seasonal Temperature}^*\text{Latitude} + \beta10^*\text{Seasonal Precipitation}^*\text{Latitude} + \\ \beta11^*\text{Stocking} + \beta12^*\text{Disturbances} + \beta13^*\text{Physiographic Class} + \beta14^*\text{Aspect} + \\ \beta15^*\text{Slope} + \beta16^*\text{Elevation} + \beta17^*\text{Latitude} + \beta18^*\text{Longitude} + \beta19^*\text{Ownership} + \\ \beta20^*\text{Forest-Group Dummies} + \beta21^*\text{FIPS Dummies} + \beta22^*\text{Time Dummies} + \varepsilon\end{aligned} \quad (2)$$

Using the result of the regression on the combined observations of all ten forest groups (Table S1: Column 1) with the $CO_2$ and seasonal precipitation and temperature inputs for 1960 and 2023, the logged wood volume was estimated by county at 25, 50, 75, and 100 years of age for stands experiencing: 1) 1960 temperature, precipitation, and $CO_2$, 2) 2023 precipitation and 1960 temperature and $CO_2$, 3) 2023 temperature and 1960 precipitation and $CO_2$, 4) 1960 temperature and precipitation and 2023 $CO_2$, and 5) 2023 temperature, precipitation, and $CO_2$. The average impact across all counties was calculated, and then the significance of the change in wood volume across this time period was assessed for both climate, using a two-sided t-test, and elevated $CO_2$, using a one-sided t-test.

Fig. S5 disentangles the climate effects by identifying the impact of changes in temperature apart from the impact of changes in precipitation. Column 1 shows the effect of changes in precipitation patterns from 1960 to 2023 while holding $CO_2$ and temperature constant at 1960 levels. Column 2 shows the effect of changes in temperature patterns from 1960 to 2023

while holding $CO_2$ and precipitation constant at 1960 levels. Then, the combined effect of the changes in temperature and precipitation are shown (Fig. 2: Column 1). The effect of elevated $CO_2$ is not shown in isolation, as its impact is not spatially heterogeneous, but the combined effect of the changes in climate and $CO_2$ is shown (Fig. 2: Column 2). The numbers that appear in the bottom left of each pane detail the mean county impact in cubic meters per hectare and the numbers in the bottom right detail the mean percentage change.

*Analysis 2: Impact of $CO_2$ and climate change on yield of specific forest groups*

- Tables S2 & S3; Figs. S6-S25

The impacts were further disentangled by repeating the above analyses for each forest group, using the same procedures as described above. Regression results are presented in Tables S2 and S3, and estimates of the combined climate impact and the impact of both climate and elevated $CO_2$ are presented in Figs. S6-S15. Finally, the impact of the changes in precipitation and temperature on the wood volume of each forest group are shown in Figs. S16-S25.

*Analysis 3: Robustness check on aggregate impact of $CO_2$ and climate change on forest yield*

- Tables S4-S7

As a robustness check, the analysis was rerun with the climate variables interacted not with latitude but with age, as shown in Eq. (3).

$$\begin{gathered}\text{Ln(volume/hectare)} = \alpha + \beta0*\text{Ln(Lifetime CO}_2) + \beta1*1/\text{Age} + \beta2*\text{Site Class} + \\ \beta3*\text{Seasonal Temperature} + \beta4*\text{Seasonal Temperature}^2 + \beta5*\text{Seasonal Temperature}^3 + \\ \beta6*\text{Seasonal Precipitation} + \beta7*\text{Seasonal Precipitation}^2 + \beta8*\text{Seasonal Precipitation}^3 + \\ \beta9*(\text{Seasonal Temperature})/\text{Age} + \beta10*(\text{Seasonal Precipitation})/\text{Age} + \\ \beta11*\text{Stocking} + \beta12*\text{Disturbances} + \beta13*\text{Physiographic Class} + \beta14*\text{Aspect} + \\ \beta15*\text{Slope} + \beta16*\text{Elevation} + \beta17*\text{Latitude} + \beta18*\text{Longitude} + \beta19*\text{Ownership} + \\ \beta20*\text{Forest-Group Dummies} + \beta21*\text{FIPS Dummies} + \beta22*\text{Time Dummies} + \varepsilon\end{gathered} \quad (3)$$

After running the regressions (Tables S4-S6), the estimated logged wood volume for each forest group was calculated for the following environmental conditions: 1) 1960 climate and $CO_2$, 2) 2023 climate and 1960 $CO_2$, 3) 1960 climate and 2023 $CO_2$, and 4) 2023 climate and $CO_2$. This allowed us to identify the impact of both climate, using a two-sided t-test, and elevated $CO_2$, using a one-sided t-test, across the period from 1960 to 2023 by examining in the case of climate the difference between the values with 2) 2023 climate and 1960 $CO_2$ and 1) 1960 climate and $CO_2$ (Table S7).

*Analysis 4: Effect of forest planting on yield, controlling for $CO_2$ and climate*

- Tables S8 & S9; Fig. S26

Next, we examined the three commercial forest groups with focus being on the impact of forest management (Tables S8 & S9). All observations of both naturally regenerated and planted stands up to 50 years of age were used, to align with forest management practices for Slash/Longleaf and Loblolly/Shortleaf pine *(18-20)* and the data available in our study (Fig. S26). Even though White/Red/Jack pine has a longer rotation *(21-23)*, its age range was matched to that of the other two to aid in making comparisons across groups. Regressions were then run (Table S8) using the approach detailed in Eq. (4).

$$\begin{aligned} \text{Ln(volume/hectare)} = {} & \alpha + \beta0^*\text{Ln(Lifetime } CO_2) + \beta1^*1/\text{Age} + \beta2^*\text{Site Class} + \\ & \beta3^*\text{Seasonal Temperature} + \beta4^*\text{Seasonal Temperature}^2 + \beta5^*\text{Seasonal Temperature}^3 + \\ & \beta6^*\text{Seasonal Precipitation} + \beta7^*\text{Seasonal Precipitation}^2 + \beta8^*\text{Seasonal Precipitation}^3 + \\ & \beta9^*\text{(Seasonal Temperature)/Age} + \beta10^*\text{(Seasonal Precipitation)/Age} + \\ & \beta11^*\text{Management} + \beta12^*\text{Management/Age} + \\ & \beta13^*\text{Stocking} + \beta14^*\text{Disturbances} + \beta15^*\text{Physiographic Class} + \beta16^*\text{Aspect} + \\ & \beta17^*\text{Slope} + \beta18^*\text{Elevation} + \beta19^*\text{Latitude} + \beta20^*\text{Longitude} + \\ & \beta21^*\text{Ownership} + \beta22^*\text{FIPS Dummies} + \beta23^*\text{Time Dummies} + \varepsilon \end{aligned} \tag{4}$$

The difference in wood volume between naturally regenerated and planted stands was then calculated by age decile between the volume on naturally regenerated and planted plots in 2023, as in Eq. (5). Significance was assessed using a two-sided t-test.

$$\% \Delta \text{ in volume due to management} = (Vol_{2023}(management = 1) - Vol_{2023}(management = 0)) / (Vol_{2023}(management = 0)) \quad (5)$$

*Analysis 5: Aggregate volume/carbon change calculation for South (1974 to 2022).*

- Tables S10-S12; Table S13: Column 1; Fig. 3

Fig. 3 adds in an examination of the impact of two other drivers: age composition and area. The first step in this analysis used the web-based EVALIDator Version 2.1.2 tool *(24)* to ascertain data on key attributes for a select group of 12 southern states in two time periods, 1974 and 2005, for each 10-year age class from 1 to 100 (Tables S10 & S11). The first attribute in the table, volume, is the net merchantable bole wood volume in cubic meters on growing-stock trees and the second attribute is the area in hectares of timberland.

The data for the early period (1974) were used to create the baseline conditions. They were taken from USFS observations that best approximated conditions in 1974 (South Carolina, SC: 1968; Florida, FL: 1970; Alabama, AL & Georgia, GA: 1972; Louisiana, LA & North Carolina, NC: 1974; Texas, TX: 1975; Oklahoma, OK: 1976; Mississippi, MS & Virginia, VA: 1977; Arkansas, AR: 1978; Tennessee, TN: 1980). No other contiguous states had evaluations taken before 1980. The USFS data for 2005 were compiled using observations from the 2004 inventory for Florida, the 2006 inventory for Mississippi, the 2008 inventory for Louisiana, the 2010 inventory for Oklahoma, and the 2005 inventories for the other states. Spruce/Fir and Aspen/Birch were unable to be examined, as there were insufficient observations of these forest groups in these states. A regression of the form detailed in Eq. 4 was run on the combined

observations of all stands aged 1 to 150 of the eight forest groups in the South, and the resulting coefficients (Table S13: Column 1) were applied only to the USFS observations from the 12 southern states when estimating the impact of each major driver (temperature, precipitation, $CO_2$, age composition, area, and planting) in the region.

Decomposing the impacts of the drivers was done with the following process. First, the total wood volume in cubic meters that the USFS reported across all ages from 1 to 100 years was summed across all forest groups and recorded in the "1974" and "2005" columns (Fig. 3). Then, the impact of area on wood volume was calculated using the forested range that the USFS reports in hectares and applying the percentage change to the 1974 wood volume, as in Eq. (6).

$$\begin{gathered}\%\ \Delta \text{ in volume due to change in area} = \\ (\text{Timberland Area}_{(2005)}/\text{Timberland Area}_{(1974)}) * \text{Wood Volume}_{(1974)}\end{gathered} \tag{6}$$

To identify the impact of age composition, we needed the USFS-reported timberland area belonging to each age decile for 1974 and 2005 and the estimated wood volume by age decile with temperature, precipitation, and $CO_2$ fixed at their 1974 levels, which we calculated from our regression results. Using these two inputs, we were able to get at the percentage change in wood volume, which then had to be differenced from the percentage change in area to isolate the impact of the shifts in age composition. This process is shown in Eq. (7), where a = age decile.

$$\begin{gathered}\%\ \Delta \text{ in volume due to change in age composition} = \\ (\sum(\text{Timberland Area}_{(a,\ 2005)} * \text{Volume per Hectare}_{(a,\ 1974)} - \\ \sum(\text{Timberland Area}_{(a,\ 1974)} * \text{Volume per Hectare}_{(a,\ 1974)}) / \\ \sum(\text{Timberland Area}_{(a,\ 1974)} * \text{Volume per Hectare}_{(a,\ 1974)}) - \\ \%\ \Delta \text{ in volume due to change in area}\end{gathered} \tag{7}$$

Adding the impacts from the changes in area and age composition to the 1974 baseline wood volume yields a depiction of a 2005 forest where temperature, precipitation, and elevated $CO_2$ had no impact.

This hypothetical forest is then used to show the impact of changes in climate and $CO_2$. To assess the impact of temperature and precipitation, we examined how the wood volume in this 2005 forest changed when considering the changes in climate that occurred between 1974 and 2005. Using the age composition and area values that the USFS reported for 2005 for each age decile and our post-regression estimates of the wood volume per hectare given: 1) 2005 climate and 1974 $CO_2$ values and 2) 1974 climate and $CO_2$ values, we were able to identify the impact of the changes in climate, as shown in Eq. (8), where a = age decile.

$$
\begin{gathered}
\%\ \Delta \text{ in volume due to changes in climate} = \\
[\sum(\text{Timberland Area}_{(a,\ 2005)} * \text{Volume per Hectare}_{(a,\ 2005\ \text{Climate}\ \&\ 1974\ CO_2)}) - \\
\sum(\text{Timberland Area}_{(a,\ 1974)} * \text{Volume per Hectare}_{(a,\ 1974\ \text{Climate}\ \&\ CO_2)})] / \\
\sum(\text{Timberland Area}_{(a,\ 1974)} * \text{Volume per Hectare}_{(a,\ 1974\ \text{Climate}\ \&\ CO_2)}) - \\
[\sum(\text{Timberland Area}_{(a,\ 2005)} * \text{Volume per Hectare}_{(a,\ 1974\ \text{Climate}\ \&\ CO_2)}) - \\
\sum(\text{Timberland Area}_{(a,\ 1974)} * \text{Volume per Hectare}_{(a,\ 1974\ \text{Climate}\ \&\ CO_2)})] / \\
\sum(\text{Timberland Area}_{(a,\ 1974)} * \text{Volume per Hectare}_{(a,\ 1974\ \text{Climate}\ \&\ \text{CO2})})
\end{gathered}
\tag{8}
$$

Estimating the impact of $CO_2$ was handled similarly, using the process detailed in Eq. (9).

$$
\begin{gathered}
\%\ \Delta \text{ in volume due to elevated } CO_2 = \\
[\sum(\text{Timberland Area}_{(a,\ 2005)} * \text{Volume per Hectare}_{(a,\ 2005\ CO_2\&\ 1974\ \text{Climate})}) - \\
\sum(\text{Timberland Area}_{(a,\ 1974)} * \text{Volume per Hectare}_{(a,\ 1974\ CO_2\&\ \text{Climate})})] / \\
\sum(\text{Timberland Area}_{(a,\ 1974)} * \text{Volume per Hectare}_{(a,\ 1974\ CO_2\&\ \text{Climate})}) - \\
[\sum(\text{Timberland Area}_{(a,\ 2005)} * \text{Volume per Hectare}_{(a,\ 1974\ CO_2\&\ \text{Climate})}) - \\
\sum(\text{Timberland Area}_{(a,\ 1974)} * \text{Volume per Hectare}_{(a,\ 1974\ CO_2\&\ \text{Climate})})] / \\
\sum(\text{Timberland Area}_{(a,\ 1974)} * \text{Volume per Hectare}_{(a,\ 1974\ CO_2\&\ \text{Climate})})
\end{gathered}
\tag{9}
$$

Finally, to identify the impact of planting, we used the area that the USFS reported as being planted and the estimated 2005 wood volumes on naturally regenerated and planted stands by age decile with both climate and $CO_2$ at 2005 levels. Then, the difference in wood volume between naturally regenerated and planted stands was calculated by age decile between the volume on naturally regenerated and planted plots in 2005. The impact was identified through the approach detailed in Eq. (10).

$$\% \Delta \text{ in volume due to planting} = \tag{10}$$
$$[\sum(\text{Planted Timberland Area}_{(a,\ 2005)} * \text{Volume per Hectare}_{(a,\ 2005\ CO2\ \&\ Climate\ \&\ Planting)}) -$$
$$\sum(\text{Planted Timberland Area}_{(a,\ 2005)} * \text{Volume per Hectare}_{(a,\ 2005\ CO2\ \&\ Climate\ \&\ Natural\ Regeneration)})] /$$
$$\sum(\text{Planted Timberland Area}_{(a,\ 2005)} * \text{Volume per Hectare}_{(a,\ 2005\ CO2\ \&\ Climate\ \&\ Natural\ Regeneration)})$$

Fig. 3 also examines a shorter period from 2005 to 2022 using the same states and approach. The USFS data for 2005 were those used in the previous analysis (Table S11), and the late period (2022) consisted of evaluations taken in 2020 for Tennessee; 2021 for Florida, Louisiana, and Oklahoma; and 2022 for the other states (Table S12). Once again, Spruce/Fir and Aspen/Birch were unable to be examined.

*Analysis 6: Volume/carbon change calculation for individual Southern forest groups (1974 to 2022)*

- Tables S2, S3 & S14; Figs. S27-S28

Analyses for the two time periods, 1974 to 2005 and 2005 to 2022, were conducted at the individual forest group level using regression results for the five naturally regenerated forest groups in the South (Tables S2-S3) and the three pine forest groups (Table S14). The impact of each driver for the two time periods is shown in Figs. S27 and S28.

*Analysis 7: Aggregate volume/carbon change calculation for North (2005 to 2022) and West (2005 to 2021), including forest-group specific effects*

- Table S13: Columns 2 & 3; Tables S15-S19; Figs. S29-S30, and Main Text Fig. 4

Panel A of Fig. 4 focuses on a group of 21 northern states: Connecticut, CT; Delaware, DE; Iowa, IA; Illinois, IL; Indiana, IN; Kentucky, KY; Massachusetts, MA; Maryland, MD; Maine, ME; Michigan, MI; Minnesota, MN; Missouri, MO; New Hampshire, NH; New Jersey, NJ; New York, NY; Ohio, OH; Pennsylvania, PA; Rhode Island, RI; Vermont, VT; Wisconsin, WI; &

West Virginia, WV. The evaluations used for 2005 were all made in 2005 and the data for 2022 came from the inventories closest to 2022 (range: 2020 to 2022) (Tables S15-S16). Observations included nine of the ten forest groups. Slash/Longleaf pine was not included, as it does not have a footprint in the North. The observations were combined, and a regression was run (Table S13: Column 2). The results were applied only to the USFS observations from the 21 northern states. Other procedures involved in the creation of this figure were identical to those used in the creation of Fig. 3. Next, the impacts were assessed by forest group, using the procedures detailed above. Impacts are shown in Fig. S29.

Panel B of Fig. 4 focuses on a group of 15 western states: Arizona, AZ; California, CA; Colorado, CO; Idaho, ID; Kansas, KS; Montana, MT; Nebraska, NE; Nevada, NV; New Mexico, NM; North Dakota, ND; Oregon, OR; South Dakota, SD; Utah, UT; Washington, WA; & Wyoming, WY. Data for 2005 were pulled from USFS-FIA evaluations closest to 2005 (range: 1999 to 2011) (Table S17). Data for 2021 were obtained from the evaluations closest to 2021 (range: 2020 to 2022) (Table S18). The major forest groups in these states are Douglas-fir, Ponderosa pine, Fir/spruce, and Lodgepole pine. Observations of the four forest groups were combined, and then a regression was run (Table S13: Column 3). As there are many observations of older stands, this analysis focused on ages from 1 to 200 years. Other procedures were identical to those used in the creation of Fig. 3. Finally, the procedures were repeated by forest group (Table S19) and results presented in Fig. S30.

## SI Figures and Tables

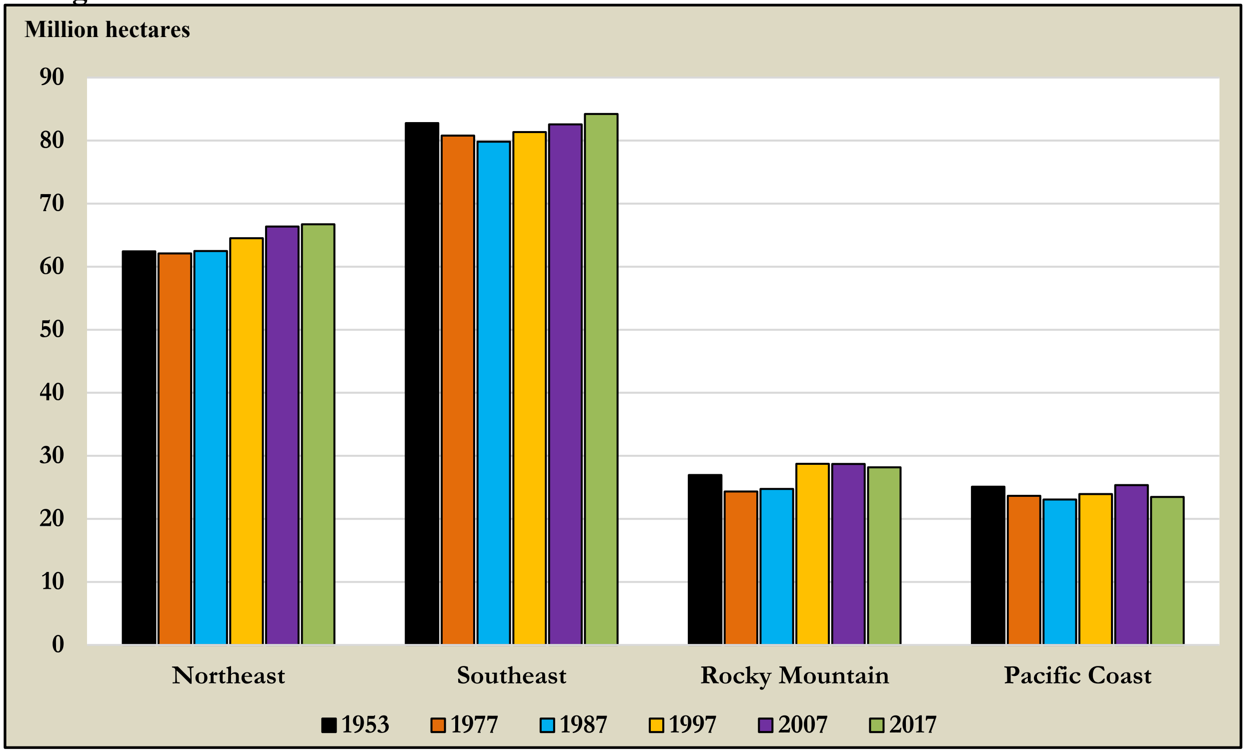


**Fig. S1. Change in timberland area from 1953 to 2017 by region.** Northeastern states: CT, DE, IA, IL, IN, MA, MD, ME, MI, MN, MO, NH, NJ, NY, OH, PA, RI, VT, WV & WI. Southeastern states: AL, AR, FL, GA, KY, LA, MS, NC, OK, SC, TN, TX & VA. Rocky Mountain states: KS, NE, ND, SD, AZ, CO, ID, MT, NV, NM, UT & WY. Pacific Coast states: CA, OR & WA. Source: USDA-Forest Service Forest Resources of the United States, 2017.

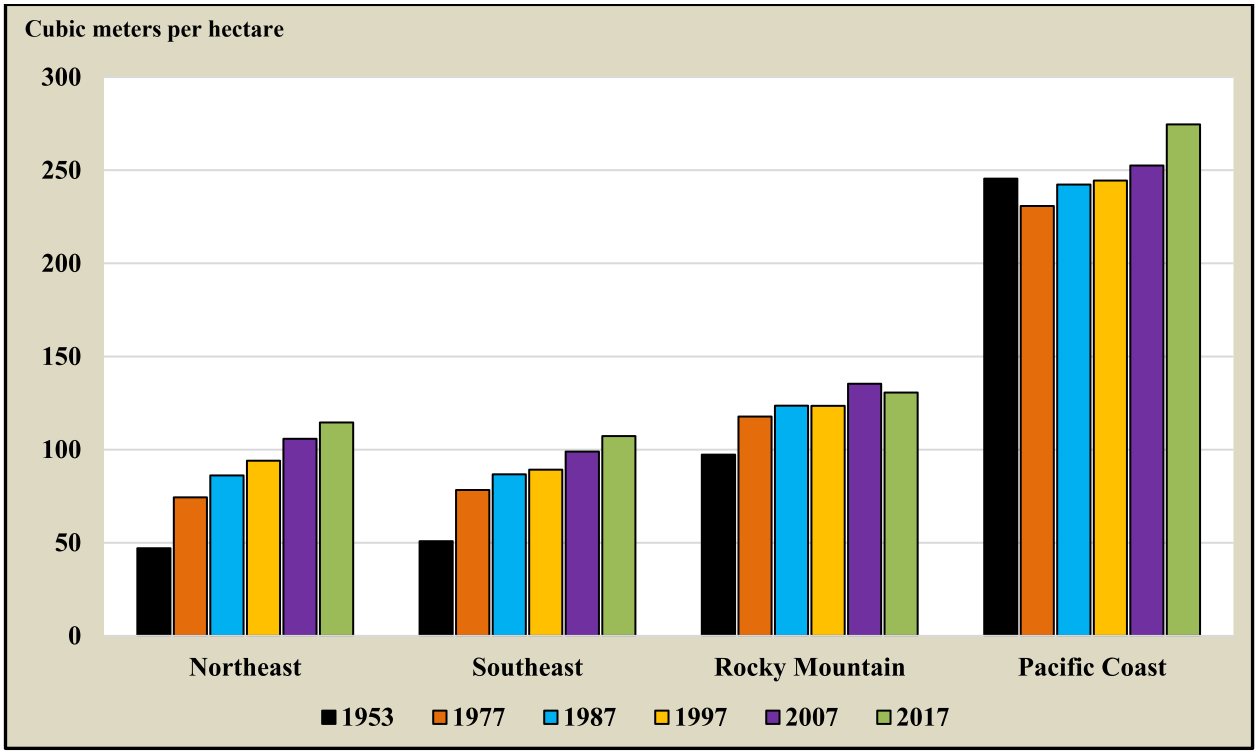


**Fig. S2. Change in timberland wood volume per area from 1953 to 2017 by region.** Northeastern states: CT, DE, IA, IL, IN, MA, MD, ME, MI, MN, MO, NH, NJ, NY, OH, PA, RI, VT, WV & WI. Southeastern states: AL, AR, FL, GA, KY, LA, MS, NC, OK, SC, TN, TX & VA. Rocky Mountain states: KS, NE, ND, SD, AZ, CO, ID, MT, NV, NM, UT & WY. Pacific Coast states: CA, OR & WA. Source: USDA-Forest Service Forest Resources of the United States, 2017.

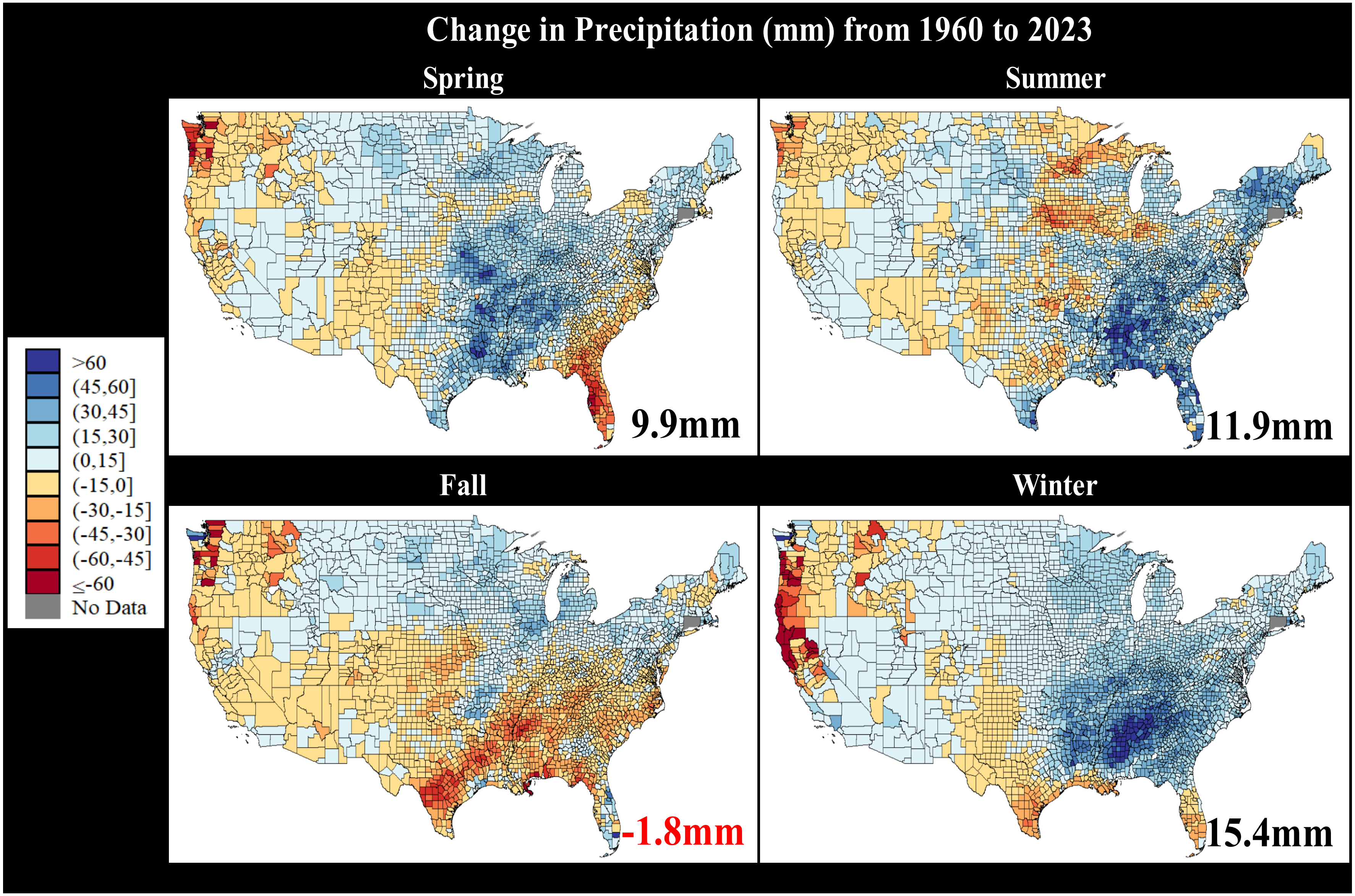


**Fig. S3. Mean change in precipitation in millimeters between 1960 and 2023, by county for the coterminous United States.** *Note:* The 1960 and 2023 values are 5-yr averages. That is, the 1960 precipitation was calculated as the average of the seasonal precipitation in the years 1956 to 1960. The numerical values in the bottom right of each pane detail the mean impact across the coterminous United States.

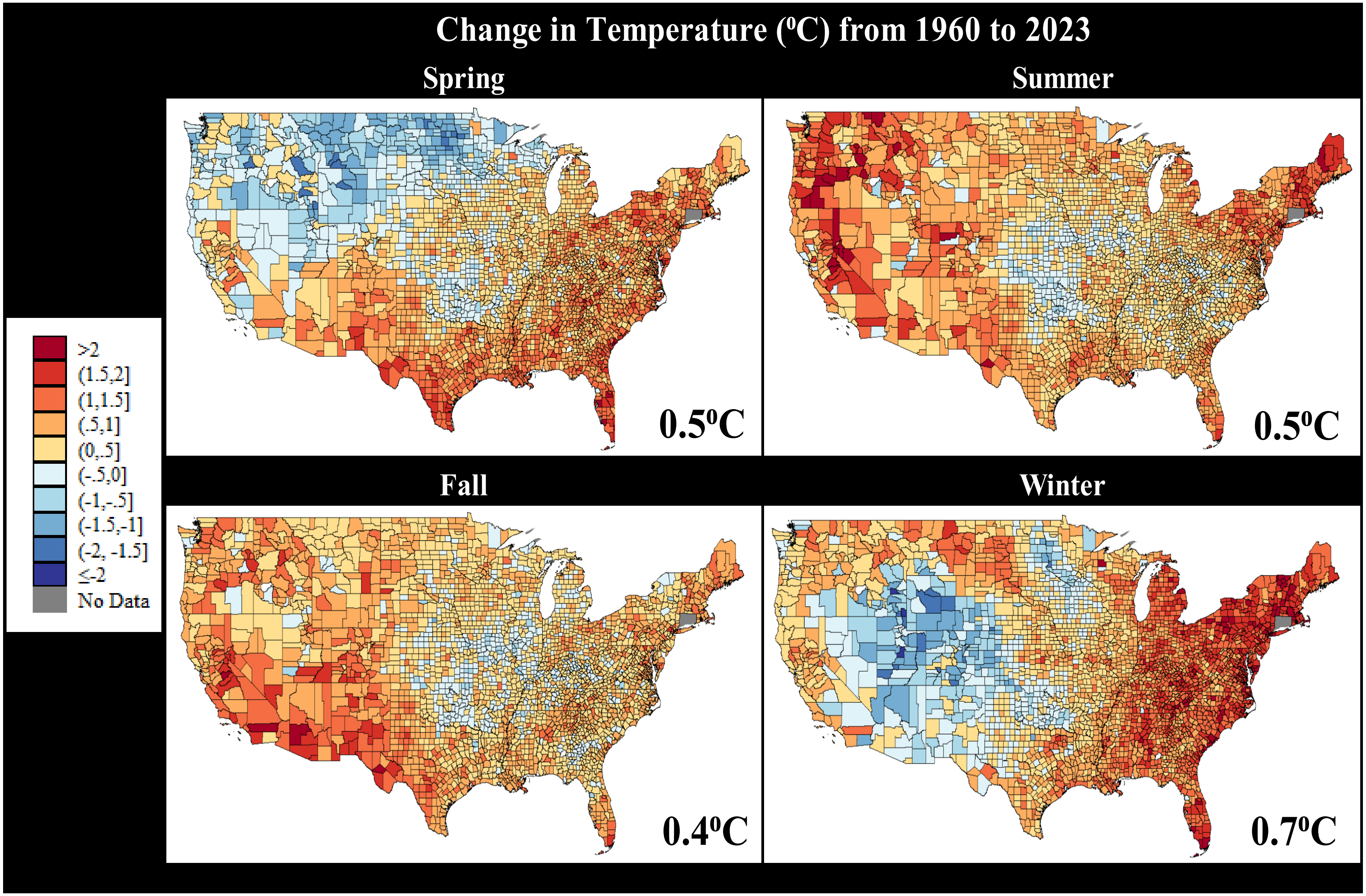


**Fig. S4. Mean change in temperature in degrees Celsius between 1960 and 2023, by county for the coterminous United States.** *Note:* The 1960 and 2023 values are 5-yr averages. That is, the 1960 temperature was calculated as the average of the seasonal temperatures in the years 1956 to 1960. The numerical values in the bottom right of each pane detail the mean impact across the coterminous United States.

**Table S1. Regression model raw output based on observations of naturally regenerated stands of ten forest groups aged 1 to 150.**

| | Natural Log of Volume per Hectare | | |
|---|---|---|---|
| Natural Log of Lifetime CO2 | 1.247*10^+00*** | 1.253*10^+00*** | |
| 1/Age | -4.474*10^+00*** | -4.423*10^+00*** | -1.076*10^+01*** |
| Mean Spring Temperature | -1.514*10^+00*** | -1.203*10^+00*** | |
| Mean Spring Temperature Squared | 1.662*10^-02*** | -2.442*10^-03 | |
| Mean Spring Temperature Cubed | -4.536*10^-04*** | 2.157*10^-04** | |
| Mean Summer Temperature | -1.814*10^+00*** | -1.591*10^+00*** | |
| Mean Summer Temperature Squared | 5.333*10^-02*** | 1.937*10^-02* | |
| Mean Summer Temperature Cubed | -8.890*10^-04*** | -3.102*10^-04* | |
| Mean Fall Temperature | 3.477*10^+00*** | 3.135*10^+00*** | |
| Mean Fall Temperature Squared | -6.940*10^-02*** | -1.735*10^-02*** | |
| Mean Fall Temperature Cubed | 1.223*10^-03*** | -3.277*10^-04*** | |
| Mean Winter Temperature | -9.947*10^-01*** | -1.117*10^+00*** | |
| Mean Winter Temperature Squared | 1.622*10^-02*** | 1.849*10^-02*** | |
| Mean Winter Temperature Cubed | 4.453*10^-04*** | 7.430*10^-04*** | |
| Mean Spring Precipitation | -8.700*10^-03** | 2.293*10^-02*** | |
| Mean Spring Precipitation Squared | 9.38*10^-05*** | -1.20*10^-05 | |
| Mean Spring Precipitation Cubed | -3.70*10^-07*** | -1.02*10^-07 | |
| Mean Summer Precipitation | 2.951*10^-03 | -8.468*10^-03*** | |
| Mean Summer Precipitation Squared | -8.19*10^-06 | 3.20*10^-05** | |
| Mean Summer Precipitation Cubed | -4.53*10^-08 | -9.70*10^-08*** | |
| Mean Fall Precipitation | 3.839*10^-02*** | 3.125*10^-02*** | |
| Mean Fall Precipitation Squared | 7.79*10^-05*** | 7.47*10^-05*** | |
| Mean Fall Precipitation Cubed | -6.52*10^-07*** | -5.74*10^-07*** | |
| Mean Winter Precipitation | 2.956*10^-02*** | 1.260*10^-02*** | |
| Mean Winter Precipitation Squared | 6.85*10^-05*** | 1.625*10^-04*** | |
| Mean Winter Precipitation Cubed | -2.30*10^-07*** | -4.78*10^-07*** | |
| Spring Temperature - Latitude Interaction | 3.410*10^-02*** | 2.966*10^-02*** | |
| Summer Temperature - Latitude Interaction | 2.141*10^-02*** | 3.151*10^-02*** | |
| Fall Temperature - Latitude Interaction | -6.327*10^-02*** | -6.525*10^-02*** | |
| Winter Temperature - Latitude Interaction | 1.748*10^-02*** | 1.852*10^-02*** | |
| Spring Precipitation - Latitude Interaction | 1.17*10^-05 | -4.990*10^-04*** | |
| Summer Precipitation - Latitude Interaction | 9.77*10^-06 | 8.80*10^-05** | |
| Fall Precipitation - Latitude Interaction | -7.464*10^-04*** | -6.558*10^-04*** | |
| Winter Precipitation - Latitude Interaction | -9.076*10^-04*** | -7.349*10^-04*** | |
| Site Class 1 | 3.770*10^-01*** | | |
| Site Class 2 | 5.616*10^-01*** | | |
| Site Class 3 | 5.288*10^-01*** | | |
| Site Class 4 | 4.022*10^-01*** | | |
| Site Class 5 | 2.338*10^-01*** | | |
| Site Class 6 | - | | |
| Time Dummy (2001 to 2023) | -1.188*10^-01*** | | |
| Time Dummy (1981 to 2000) | -6.056*10^-02*** | | |
| Time Dummy (≤1980) | - | | |
| Elevation | 1.22*10^-05*** | | |
| Slope | 4.216*10^-04*** | | |
| Disturbances | -4.796*10^-03** | | |
| Xeric Physiography | -2.073*10^-01*** | | |
| Hydric Physiography | -2.543*10^-01*** | | |
| Mesic Physiography | - | | |
| North-facing Aspect | 1.526*10^-02*** | | |
| Null Aspect | -3.070*10^-02*** | | |
| South-facing Aspect | - | | |
| Private Land Ownership | 2.117*10^-02*** | | |
| Latitude | 1.055*10^-01** | | |
| Longitude | 6.201*10^-03* | | |
| Stocking Density 1 | 7.210*10^-01*** | | |
| Stocking Density 2 | 8.134*10^-01*** | | |
| Stocking Density 3 | 5.643*10^-01*** | | |
| Stocking Density 4 | 2.078*10^-01*** | | |
| Stocking Density 5 | - | | |
| White/Red/Jack | 3.386*10^-01*** | 3.893*10^-01*** | |
| Spruce/Fir | -2.181*10^-01*** | -3.572*10^-01*** | |
| Slash/Longleaf | 1.411*10^-01*** | 1.926*10^-01*** | |
| Loblolly/Shortleaf | 3.349*10^-01*** | 4.720*10^-01*** | |
| Oak/Pine | 6.698*10^-02*** | 8.172*10^-02*** | |
| Oak/Gum/Cypress | 2.416*10^-01*** | 1.790*10^-01*** | |
| Elm/Ash/Cottonwood | 5.541*10^-02*** | -7.020*10^-02*** | |
| Maple/Beech/Birch | 6.596*10^-02*** | 1.024*10^-01*** | |
| Aspen/Birch | -3.734*10^-02*** | 1.167*10^-01*** | |
| Oak/Hickory | - | - | |
| Constant | -8.059*10^+00*** | -6.276*10^+00*** | 4.953*10^+00*** |
| County Fixed Effects | Yes | Yes | No |
| Observations | 1,282,876 | 1,282,876 | 1,282,876 |
| R-squared | 0.504 | 0.423 | 0.135 |

*Note:* The ten forest groups: Elm/Ash/Cottonwood, Oak/Pine, Oak/Hickory, Maple/Beech/Birch, White/Red/Jack Pine, Aspen/Birch, Spruce/Fir, Oak/Gum/Cypress, Loblolly/Shortleaf Pine, and Slash/Shortleaf Pine. ***p<0.01//**p<0.05//*p<0.10

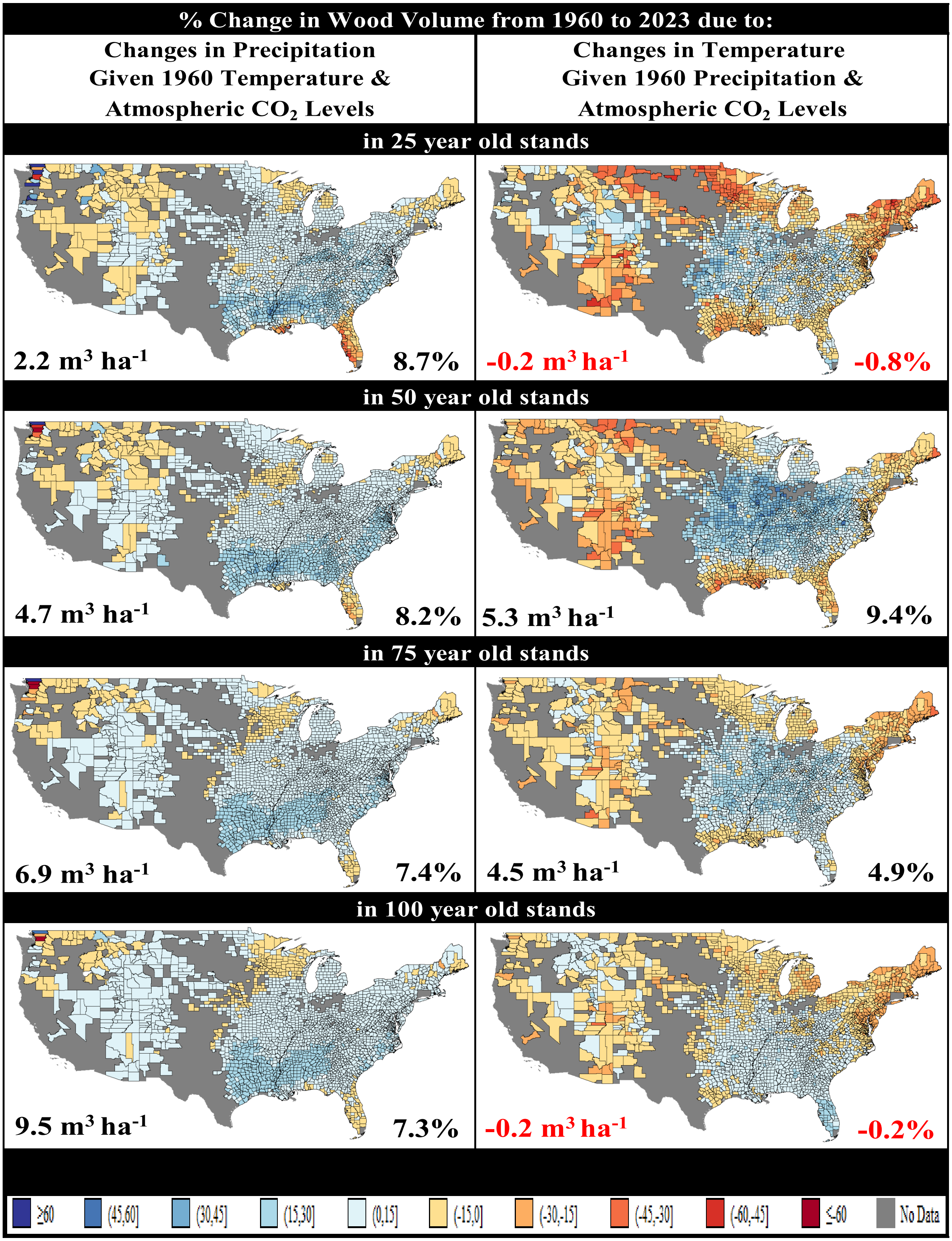


**Fig. S5. Estimated wood volume change from 1960 to 2023, by climate component, age, and county, based on observations of naturally regenerated stands aged 1 to 150 of all forest groups.** *Note:* The numerical values in the bottom left of each pane detail the mean county impact in cubic meters per hectare and the values in the bottom right of each pane detail the mean percentage change. The ten forest groups: Elm/Ash/Cottonwood, Oak/Pine, Oak/Hickory, Maple/Beech/Birch, White/Red/Jack Pine, Aspen/Birch, Spruce/Fir, Oak/Gum/Cypress, Loblolly/Shortleaf Pine, and Slash/Shortleaf Pine.

**Table S2. Regression model raw output by forest group, based on observations of naturally regenerated stands aged 1 to 150.**

| | Natural Log of Volume per Hectare | | | | |
|---|---|---|---|---|---|
| Variables | White/Red Jack | Slash/ Longleaf | Loblolly/ Shortleaf | Spruce/ Fir | Oak/ Pine |
| Natural Log of Lifetime $CO_2$ | 1.186*10^+00*** | 1.179*10^+00*** | 1.107*10^+00*** | 1.236*10^+00*** | 1.170*10^+00*** |
| 1/Age | -5.411*10^+00*** | -2.803*10^+00*** | -3.224*10^-01** | -6.197*10^+00*** | -2.331*10^+00*** |
| Mean Spring Temperature | 4.537*10^+00*** | 1.708*10^+01** | 1.097*10^-01 | -8.916*10^+00*** | -3.428*10^+00*** |
| Mean Spring Temperature Squared | -8.821*10^-02*** | -6.005*10^-01* | -3.069*10^-01*** | 1.722*10^-03 | -9.122*10^-03 |
| Mean Spring Temperature Cubed | 1.215*10^-03 | 9.691*10^-03* | 6.276*10^-03*** | -5.293*10^-03 | 8.093*10^-04** |
| Mean Summer Temperature | -2.536*10^+01*** | -9.623*10^+00 | 1.509*10^+01*** | -3.795*10^+01*** | 3.661*10^+00** |
| Mean Summer Temperature Squared | 1.557*10^+00*** | 3.948*10^-01 | -5.673*10^-01*** | 1.831*10^+00*** | -2.023*10^-02 |
| Mean Summer Temperature Cubed | -2.589*10^-02*** | -5.248*10^-03 | 7.266*10^-03*** | -3.148*10^-02** | -1.438*10^-04 |
| Mean Fall Temperature | -1.500*10^+01*** | -7.623*10^+00 | 3.563*10^+00** | 2.421*10^-01 | -3.449*10^-01 |
| Mean Fall Temperature Squared | -8.556*10^-02* | 4.554*10^-01* | 1.894*10^-01*** | 8.552*10^-02 | 7.318*10^-02*** |
| Mean Fall Temperature Cubed | 8.489*10^-03*** | -6.951*10^-03* | -4.762*10^-03*** | -4.616*10^-03 | -1.790*10^-03*** |
| Mean Winter Temperature | 3.647*10^+00*** | -5.533*10^+00*** | -1.135*10^+00** | 5.225*10^+00*** | -3.213*10^-01 |
| Mean Winter Temperature Squared | -5.529*10^-03 | -1.967*10^-02 | 2.794*10^-02*** | 1.217*10^-02 | 5.243*10^-03 |
| Mean Winter Temperature Cubed | 2.027*10^-03*** | 1.433*10^-03 | -2.007*10^-04 | 7.825*10^-04 | 4.608*10^-04*** |
| Mean Spring Precipitation | -6.845*10^-01*** | -2.186*10^-01*** | -4.402*10^-02*** | 1.675*10^-01 | -2.986*10^-02** |
| Mean Spring Precipitation Squared | 1.818*10^-03*** | 4.507*10^-04** | 3.472*10^-04*** | -2.699*10^-04 | -3.53*10^-05 |
| Mean Spring Precipitation Cubed | -3.08*10^-06*** | -1.13*10^-06** | -8.69*10^-07*** | 2.10*10^-06 | 8.04*10^-08 |
| Mean Summer Precipitation | 1.250*10^-01*** | -1.306*10^-01*** | 3.078*10^-02*** | -1.886*10^-01** | 9.531*10^-02*** |
| Mean Summer Precipitation Squared | -2.601*10^-03*** | 6.454*10^-04*** | 1.756*10^-04*** | 1.418*10^-03** | -3.266*10^-04*** |
| Mean Summer Precipitation Cubed | 8.80*10^-06*** | -1.19*10^-06*** | -5.48*10^-07*** | -5.04*10^-06*** | 5.95*10^-07*** |
| Mean Fall Precipitation | 3.626*10^-02 | 8.452*10^-02*** | -1.050*10^-02 | -4.389*10^-02 | -3.253*10^-02** |
| Mean Fall Precipitation Squared | 1.335*10^-03*** | 4.332*10^-04** | 2.042*10^-04*** | -4.730*10^-04* | 9.513*10^-04*** |
| Mean Fall Precipitation Cubed | -4.96*10^-06*** | -1.84*10^-06*** | -6.63*10^-07*** | 1.57*10^-06 | -3.57*10^-06*** |
| Mean Winter Precipitation | 2.366*10^-01*** | -3.170*10^-02 | 5.363*10^-02*** | -3.947*10^-01*** | 3.297*10^-02** |
| Mean Winter Precipitation Squared | -2.842*10^-04** | 3.424*10^-04* | -2.289*10^-04** | -3.305*10^-04 | -2.629*10^-04*** |
| Mean Winter Precipitation Cubed | 3.27*10^-08 | -1.48*10^-06** | 6.70*10^-07** | 2.92*10^-06** | 6.46*10^-07*** |
| Spring Temperature - Latitude Interaction | -7.534*10^-02*** | -1.702*10^-01** | 1.318*10^-01*** | 1.939*10^-01*** | 8.402*10^-02*** |
| Summer Temperature - Latitude Interaction | -1.196*10^-01*** | -3.174*10^-03 | -2.388*10^-03 | 6.388*10^-02* | -6.559*10^-02*** |
| Fall Temperature - Latitude Interaction | 3.230*10^-01*** | -6.151*10^-02 | -1.692*10^-01*** | -1.579*10^-02 | -1.504*10^-02 |
| Winter Temperature - Latitude Interaction | -1.046*10^-01*** | 1.699*10^-01*** | 2.120*10^-02* | -1.181*10^-01*** | 2.869*10^-03 |
| Spring Precipitation - Latitude Interaction | 1.034*10^-02*** | 5.158*10^-03*** | -1.380*10^-04 | -3.515*10^-03 | 9.986*10^-04*** |
| Summer Precipitation - Latitude Interaction | 3.045*10^-03*** | 6.349*10^-04 | -1.354*10^-03*** | 1.192*10^-03 | -1.265*10^-03*** |
| Fall Precipitation - Latitude Interaction | -3.473*10^-03*** | -3.373*10^-03*** | -1.660*10^-04 | 1.421*10^-03 | -1.176*10^-03*** |
| Winter Precipitation - Latitude Interaction | -4.462*10^-03*** | 2.651*10^-04 | -7.226*10^-04*** | 8.629*10^-03*** | 2.113*10^-04 |
| Site Class 1 | 4.389*10^-01*** | 6.485*10^-01*** | 2.049*10^-01*** | | 6.517*10^-02 |
| Site Class 2 | 7.558*10^-01*** | 4.215*10^-01*** | 8.219*10^-01*** | 4.181*10^-01 | 6.974*10^-01*** |
| Site Class 3 | 5.969*10^-01*** | 7.617*10^-01*** | 7.306*10^-01*** | 6.012*10^-01*** | 6.509*10^-01*** |
| Site Class 4 | 4.274*10^-01*** | 6.237*10^-01*** | 5.452*10^-01*** | 5.024*10^-01*** | 4.835*10^-01*** |
| Site Class 5 | 2.710*10^-01*** | 3.833*10^-01*** | 3.333*10^-01*** | 4.434*10^-01*** | 2.960*10^-01*** |
| Site Class 6 | - | - | - | - | - |
| Time Dummy (2001 to 2023) | 3.267*10^-01*** | -1.677*10^-01*** | -6.407*10^-02*** | 3.168*10^-01*** | -9.494*10^-02*** |
| Time Dummy (1981 to 2000) | 4.145*10^-02 | -3.188*10^-02 | -2.495*10^-02* | 1.996*10^-01*** | -7.688*10^-02*** |
| Time Dummy (≤1980) | - | - | - | - | - |
| Elevation | 1.27*10^-05 | -1.55*10^-05 | -3.67*10^-05** | -9.89*10^-05*** | 3.77*10^-05*** |
| Slope | 2.187*10^-03*** | 3.858*10^-03** | -1.582*10^-03*** | 2.209*10^-03*** | 2.268*10^-04 |
| Disturbances | 9.151*10^-04 | -6.940*10^-03 | 2.297*10^-02*** | -3.670*10^-04 | -4.018*10^-02*** |
| Xeric Physiography | -1.532*10^-01*** | -9.176*10^-02*** | -1.499*10^-01*** | -3.413*10^-01*** | -2.032*10^-01*** |
| Hydric Physiography | -4.020*10^-01*** | -2.967*10^-02* | -1.838*10^-01*** | -3.973*10^-01*** | 1.402*10^-02 |
| Mesic Physiography | - | - | - | - | - |
| North-facing Aspect | -4.662*10^-03 | 4.916*10^-02** | 1.499*10^-02** | 3.212*10^-02** | 1.868*10^-02*** |
| Null Aspect | 1.021*10^-02 | -4.598*10^-02** | 3.069*10^-02*** | -1.966*10^-01*** | 4.257*10^-03 |
| South-facing Aspect | - | - | - | - | - |
| Private Land Ownership | 3.101*10^-02*** | 7.284*10^-02*** | -4.751*10^-03 | 1.192*10^-01*** | -1.239*10^-02* |
| Latitude | -1.435*10^+00*** | 2.585*10^+00** | 1.010*10^+00*** | -3.400*10^+00*** | 7.904*10^-01*** |
| Longitude | -1.234*10^-01*** | 3.027*10^-02 | -1.189*10^-02 | 1.151*10^-01*** | -3.021*10^-02* |
| Stocking Density 1 | 1.152*10^+00*** | 1.731*10^+00*** | 1.193*10^+00*** | -4.729*10^-02 | 6.329*10^-01*** |
| Stocking Density 2 | 9.251*10^-01*** | 1.286*10^+00*** | 9.801*10^-01*** | 4.741*10^-01*** | 7.019*10^-01*** |
| Stocking Density 3 | 6.477*10^-01*** | 1.003*10^+00*** | 6.362*10^-01*** | 5.251*10^-01*** | 4.809*10^-01*** |
| Stocking Density 4 | 2.076*10^-01*** | 4.612*10^-01*** | 2.125*10^-01*** | 3.272*10^-01*** | 1.601*10^-01*** |
| Stocking Density 5 | - | - | - | - | - |
| Constant | 2.416*10^+02*** | -6.832*10^+00 | -1.877*10^+02*** | 3.803*10^+02*** | -4.919*10^+01*** |
| Observations | 29,796 | 22,271 | 104,997 | 64,867 | 97,667 |
| R-squared | 0.642 | 0.592 | 0.594 | 0.408 | 0.564 |

Note: All regressions employed county fixed effects. ***p<0.01//**p<0.05//*p<0.10

**Table S3. Regression model raw output by forest group, based on observations of naturally regenerated stands aged 1 to 150.**

| | Natural Log of Volume per Hectare | | | | |
|---|---|---|---|---|---|
| Variables | Oak/ Hickory | Oak/Gum/ Cypress | Elm/Ash Cottonwood | Maple/Beech/ Birch | Aspen/ Birch |
| Natural Log of Lifetime $CO_2$ | 1.185*10^+00*** | 1.202*10^+00*** | 1.247*10^+00*** | 1.147*10^+00*** | 1.425*10^+00*** |
| 1/Age | -3.828*10^+00*** | -2.554*10^+00*** | -4.652*10^+00*** | -4.759*10^+00*** | -6.457*10^+00*** |
| Mean Spring Temperature | -6.345*10^+00*** | -4.270*10^+00** | -2.984*10^+00*** | 1.017*10^+00** | 2.486*10^+00*** |
| Mean Spring Temperature Squared | 7.854*10^-02*** | 1.065*10^-01 | 9.394*10^-02*** | 5.573*10^-02*** | 1.279*10^-01*** |
| Mean Spring Temperature Cubed | -8.191*10^-04*** | -1.335*10^-03 | -2.213*10^-03*** | -3.998*10^-03*** | -1.059*10^-02*** |
| Mean Summer Temperature | -8.364*10^+00*** | 3.643*10^+01*** | -2.185*10^+00 | -6.410*10^+00*** | -3.993*10^+00*** |
| Mean Summer Temperature Squared | 3.285*10^-01*** | -1.619*10^+00*** | 5.490*10^-02 | 2.183*10^-01*** | 1.174*10^-02 |
| Mean Summer Temperature Cubed | -4.728*10^-03*** | 2.155*10^-02*** | -1.124*10^-03 | -3.214*10^-03*** | 5.152*10^-04 |
| Mean Fall Temperature | 6.875*10^+00*** | 7.302*10^+00*** | 3.565*10^+00*** | -4.827*10^-01 | 5.698*10^+00*** |
| Mean Fall Temperature Squared | -1.464*10^-01*** | -1.085*10^-01 | -1.377*10^-01*** | 6.741*10^-02*** | -1.161*10^-01*** |
| Mean Fall Temperature Cubed | 2.386*10^-03*** | 1.072*10^-03 | 2.934*10^-03*** | -1.870*10^-03** | 4.194*10^-03*** |
| Mean Winter Temperature | 2.382*10^-01 | -1.455*10^+00* | -2.576*10^-01 | -2.527*10^-01 | -1.723*10^+00*** |
| Mean Winter Temperature Squared | 2.099*10^-03 | 5.530*10^-03 | 1.033*10^-02*** | 3.657*10^-02*** | 5.526*10^-02*** |
| Mean Winter Temperature Cubed | 3.927*10^-04*** | 3.049*10^-04 | 6.697*10^-04*** | 2.299*10^-03*** | 2.129*10^-03*** |
| Mean Spring Precipitation | 2.459*10^-02*** | -1.558*10^-02 | 5.973*10^-02*** | 3.248*10^-01*** | -7.565*10^-02*** |
| Mean Spring Precipitation Squared | -2.307*10^-04*** | 6.226*10^-04*** | -2.364*10^-04** | -1.837*10^-03*** | 4.35*10^-05 |
| Mean Spring Precipitation Cubed | 5.05*10^-07*** | -1.64*10^-06*** | 4.34*10^-07 | 6.10*10^-06*** | -3.87*10^-07 |
| Mean Summer Precipitation | -2.577*10^-03 | 8.372*10^-02*** | -4.837*10^-02*** | 1.960*10^-03 | -4.134*10^-02** |
| Mean Summer Precipitation Squared | 3.09*10^-05 | -1.993*10^-04*** | 3.008*10^-04*** | -8.109*10^-04*** | 8.789*10^-04*** |
| Mean Summer Precipitation Cubed | -7.81*10^-08 | 2.86*10^-07*** | -7.01*10^-07*** | 2.28*10^-06*** | -2.74*10^-06*** |
| Mean Fall Precipitation | -1.719*10^-02*** | 5.796*10^-02*** | 2.266*10^-02* | 1.154*10^-01*** | 1.051*10^-01*** |
| Mean Fall Precipitation Squared | 2.114*10^-04*** | -1.829*10^-04*** | 1.767*10^-04** | -3.927*10^-04*** | -1.787*10^-04*** |
| Mean Fall Precipitation Cubed | -1.04*10^-06*** | 1.45*10^-07 | -6.68*10^-07*** | 1.26*10^-06*** | -1.46*10^-09 |
| Mean Winter Precipitation | 3.073*10^-02*** | 1.056*10^-01*** | 7.622*10^-02*** | -1.653*10^-01*** | -2.433*10^-02 |
| Mean Winter Precipitation Squared | 7.28*10^-05** | -1.634*10^-04 | 4.19*10^-05 | 5.728*10^-04*** | -8.05*10^-06 |
| Mean Winter Precipitation Cubed | -1.52*10^-07 | 4.03*10^-07 | -1.77*10^-07 | -1.62*10^-06*** | -4.62*10^-08 |
| Spring Temperature - Latitude Interaction | 1.256*10^-01*** | 4.803*10^-02 | 4.787*10^-02*** | -2.261*10^-02** | -5.976*10^-02*** |
| Summer Temperature - Latitude Interaction | 1.780*10^-02*** | 1.017*10^-01*** | 3.935*10^-02*** | 3.255*10^-02*** | 7.474*10^-02*** |
| Fall Temperature - Latitude Interaction | -1.136*10^-01*** | -1.255*10^-01*** | -4.736*10^-02** | -8.004*10^-03 | -1.062*10^-01*** |
| Winter Temperature - Latitude Interaction | -1.278*10^-02** | 3.670*10^-02* | -1.747*10^-03 | -7.994*10^-04 | 3.970*10^-02*** |
| Spring Precipitation - Latitude Interaction | 2.238*10^-04** | -1.870*10^-03*** | -7.396*10^-04*** | -3.638*10^-03*** | 1.631*10^-03*** |
| Summer Precipitation - Latitude Interaction | -1.821*10^-04** | -1.500*10^-03*** | 2.305*10^-04 | 2.092*10^-03*** | -9.651*10^-04** |
| Fall Precipitation - Latitude Interaction | 4.829*10^-04*** | -5.581*10^-04** | -9.369*10^-04*** | -1.657*10^-03*** | -1.616*10^-03*** |
| Winter Precipitation - Latitude Interaction | -1.086*10^-03*** | -2.574*10^-03*** | -2.080*10^-03*** | 2.644*10^-03*** | 3.346*10^-04 |
| Site Class 1 | 2.121*10^-01*** | 3.301*10^-01*** | 6.637*10^-01*** | 1.169*10^-01 | 5.520*10^-01*** |
| Site Class 2 | 4.410*10^-01*** | 3.814*10^-01*** | 4.645*10^-01*** | 3.715*10^-01*** | 6.389*10^-01*** |
| Site Class 3 | 4.382*10^-01*** | 3.869*10^-01*** | 4.316*10^-01*** | 1.599*10^-01*** | 6.044*10^-01*** |
| Site Class 4 | 3.398*10^-01*** | 3.288*10^-01*** | 3.496*10^-01*** | 1.653*10^-01*** | 4.564*10^-01*** |
| Site Class 5 | 1.746*10^-01*** | 2.313*10^-01*** | 2.252*10^-01*** | 1.071*10^-01*** | 2.205*10^-01*** |
| Site Class 6 | - | - | - | - | - |
| Time Dummy (2001 to 2023) | -1.688*10^-01*** | -1.964*10^-01*** | -1.195*10^-01*** | 5.757*10^-02*** | -3.301*10^-01*** |
| Time Dummy (1981 to 2000) | -1.357*10^-01*** | -1.003*10^-01*** | -5.962*10^-02** | -4.922*10^-02*** | 8.801*10^-02*** |
| Time Dummy (≤1980) | - | - | - | - | - |
| Elevation | 3.86*10^-05*** | 1.546*10^-04*** | -4.53*10^-05*** | -4.18*10^-05*** | 8.33*10^-05*** |
| Slope | 7.941*10^-04*** | -3.084*10^-03*** | -1.468*10^-03*** | 8.59*10^-05 | 1.530*10^-03*** |
| Disturbances | -2.271*10^-03 | -5.421*10^-02*** | 7.339*10^-03 | -1.261*10^-02*** | 2.985*10^-02*** |
| Xeric Physiography | -2.008*10^-01*** | -4.909*10^-01*** | -3.631*10^-01*** | -3.362*10^-01*** | -7.819*10^-02*** |
| Hydric Physiography | 5.076*10^-02*** | -2.617*10^-02*** | -1.895*10^-01*** | -1.863*10^-01*** | -4.017*10^-01*** |
| Mesic Physiography | - | - | - | - | - |
| North-facing Aspect | 1.993*10^-02*** | 4.086*10^-02** | 2.400*10^-02** | 3.249*10^-04 | 2.320*10^-02*** |
| Null Aspect | -5.300*10^-04 | -4.419*10^-02*** | -3.912*10^-02*** | -7.141*10^-02*** | -7.369*10^-02*** |
| South-facing Aspect | - | - | - | - | - |
| Private Land Ownership | 1.278*10^-02*** | -2.118*10^-03 | 1.517*10^-02** | -5.219*10^-02*** | 9.891*10^-03* |
| Latitude | -3.506*10^-01*** | -6.839*10^-01 | -4.787*10^-01* | -3.718*10^-01** | 1.639*10^-02 |
| Longitude | -1.936*10^-02*** | 7.741*10^-03 | -5.390*10^-02*** | -3.499*10^-03 | 3.165*10^-02*** |
| Stocking Density 1 | 7.525*10^-01*** | 1.041*10^+00*** | 7.872*10^-01*** | 7.531*10^-01*** | 4.847*10^-01*** |
| Stocking Density 2 | 7.606*10^-01*** | 9.429*10^-01*** | 7.526*10^-01*** | 7.810*10^-01*** | 9.033*10^-01*** |
| Stocking Density 3 | 5.152*10^-01*** | 6.415*10^-01*** | 5.212*10^-01*** | 4.900*10^-01*** | 7.477*10^-01*** |
| Stocking Density 4 | 1.958*10^-01*** | 2.764*10^-01*** | 1.772*10^-01*** | 1.341*10^-01*** | 3.460*10^-01*** |
| Stocking Density 5 | - | - | - | - | - |
| Constant | 6.112*10^+01*** | -3.092*10^+02*** | 8.741*10^+00 | 4.240*10^+01*** | -7.043*10^+00 |
| Observations | 471,966 | 88,241 | 101,220 | 162,130 | 110,632 |
| R-squared | 0.514 | 0.541 | 0.523 | 0.506 | 0.521 |

Note: All regressions employed county fixed effects. ***p<0.01//**p<0.05//*p<0.10

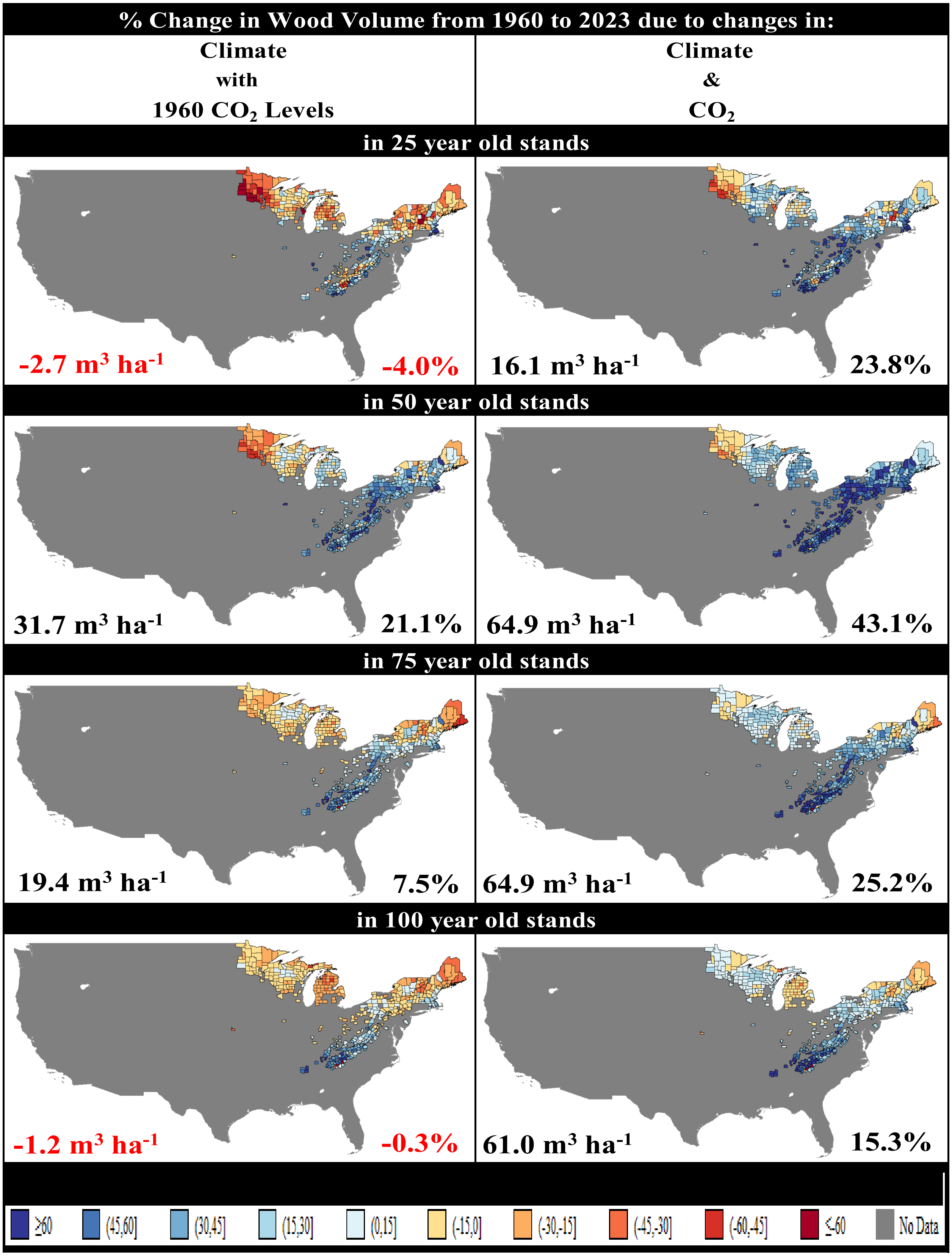


**Fig. S6. Estimated change in wood volume from 1960 to 2023, by county and age, based on observations of naturally regenerated White/Red/Jack stands aged 1 to 150.** *Note:* The numerical values in the bottom left of each pane detail the mean county impact in cubic meters per hectare and the values in the bottom right of each pane detail the mean percentage change.

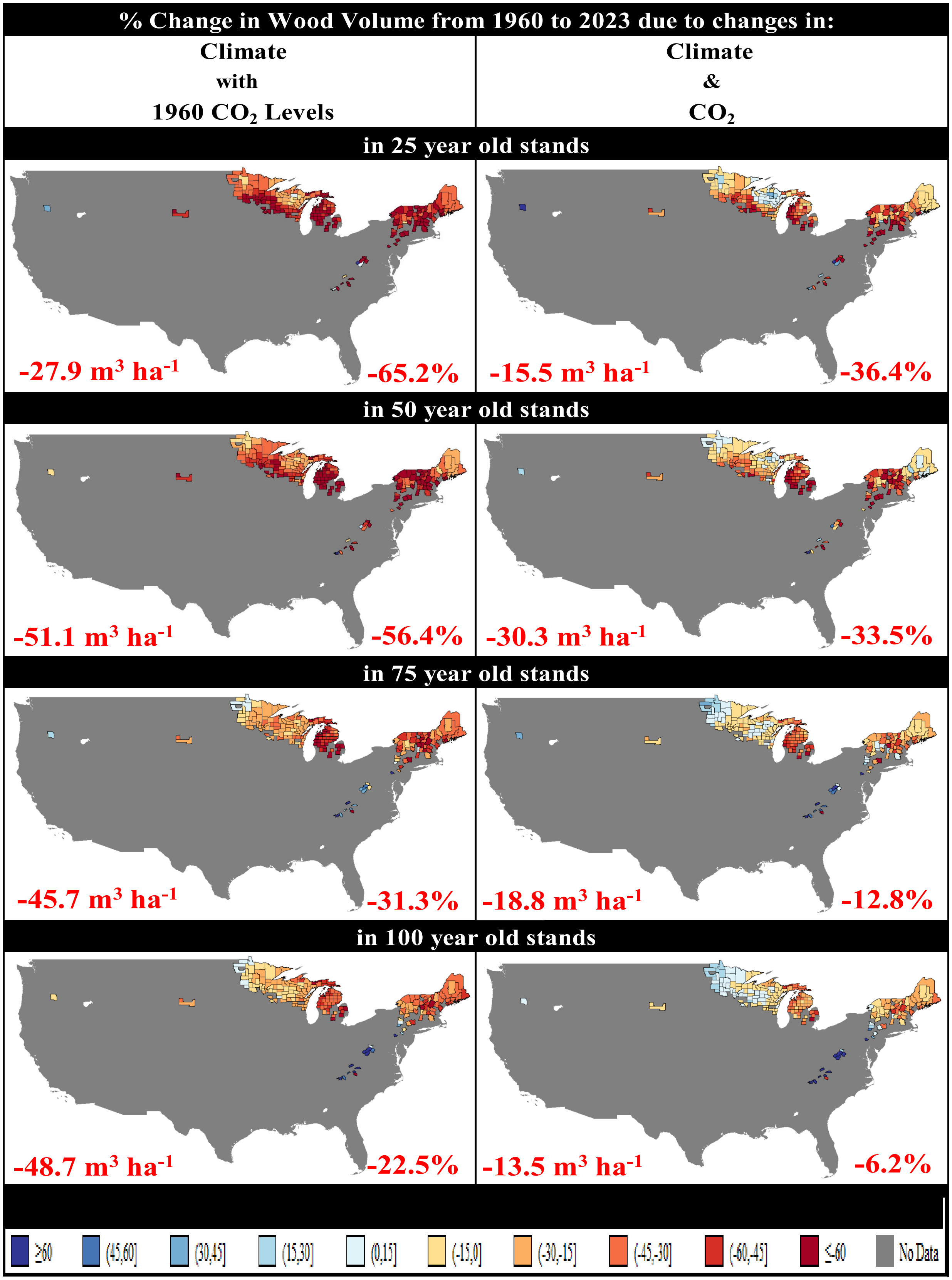


**Fig. S7. Estimated change in wood volume from 1960 to 2023, by county and age, based on observations of naturally regenerated Spruce/Fir stands aged 1 to 150.** *Note:* The numerical values in the bottom left of each pane detail the mean county impact in cubic meters per hectare and the values in the bottom right of each pane detail the mean percentage change.

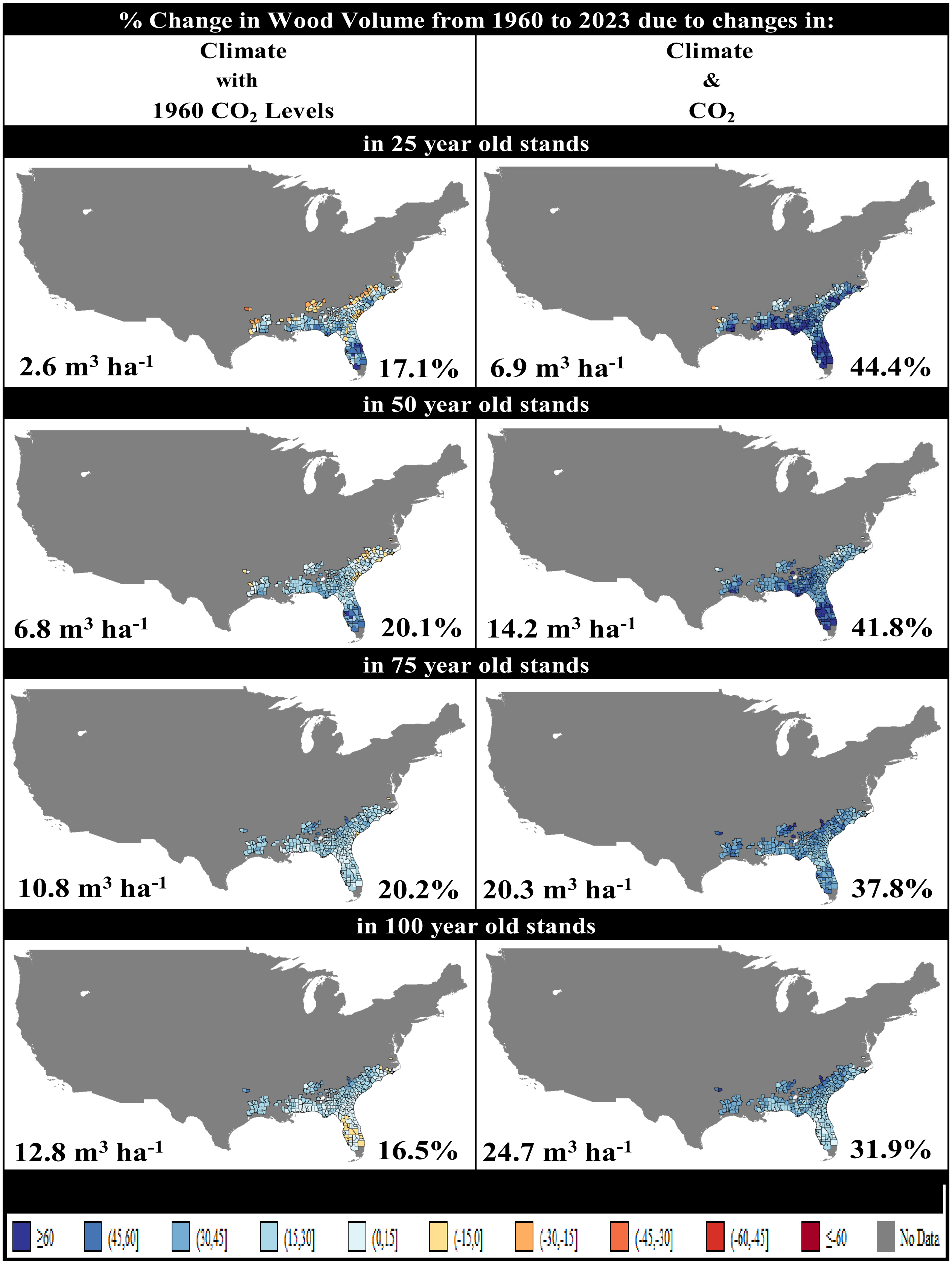


**Fig. S8. Estimated change in wood volume from 1960 to 2023, by county and age, based on observations of naturally regenerated Slash/Longleaf pine stands aged 1 to 150.** *Note:* The numerical values in the bottom left of each pane detail the mean county impact in cubic meters per hectare and the values in the bottom right of each pane detail the mean percentage change.

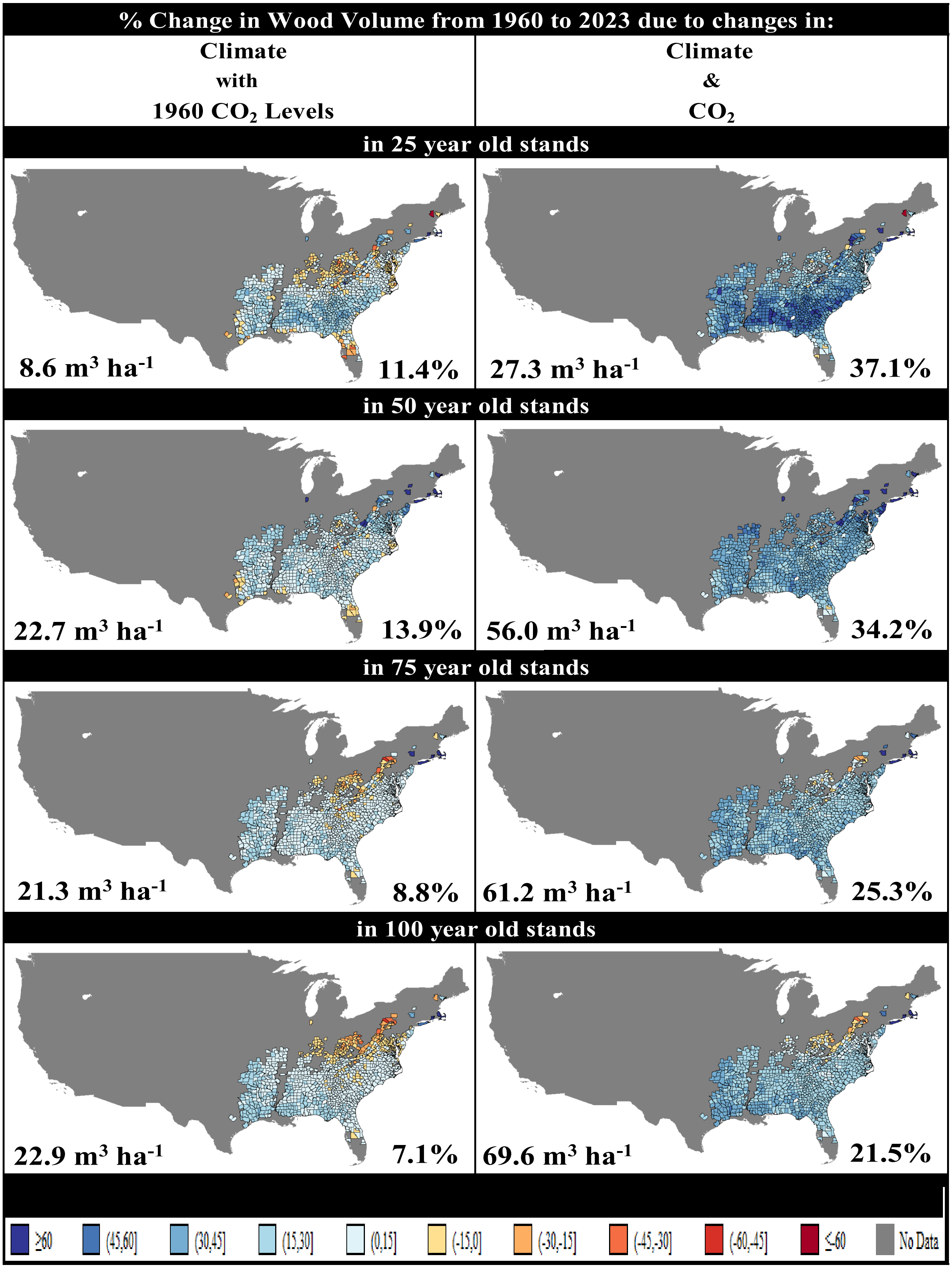


**Fig. S9. Estimated change in wood volume from 1960 to 2023, by county and age, based on observations of naturally regenerated Loblolly/Shortleaf pine stands aged 1 to 150.** *Note:* The numerical values in the bottom left of each pane detail the mean county impact in cubic meters per hectare and the values in the bottom right of each pane detail the mean percentage change.

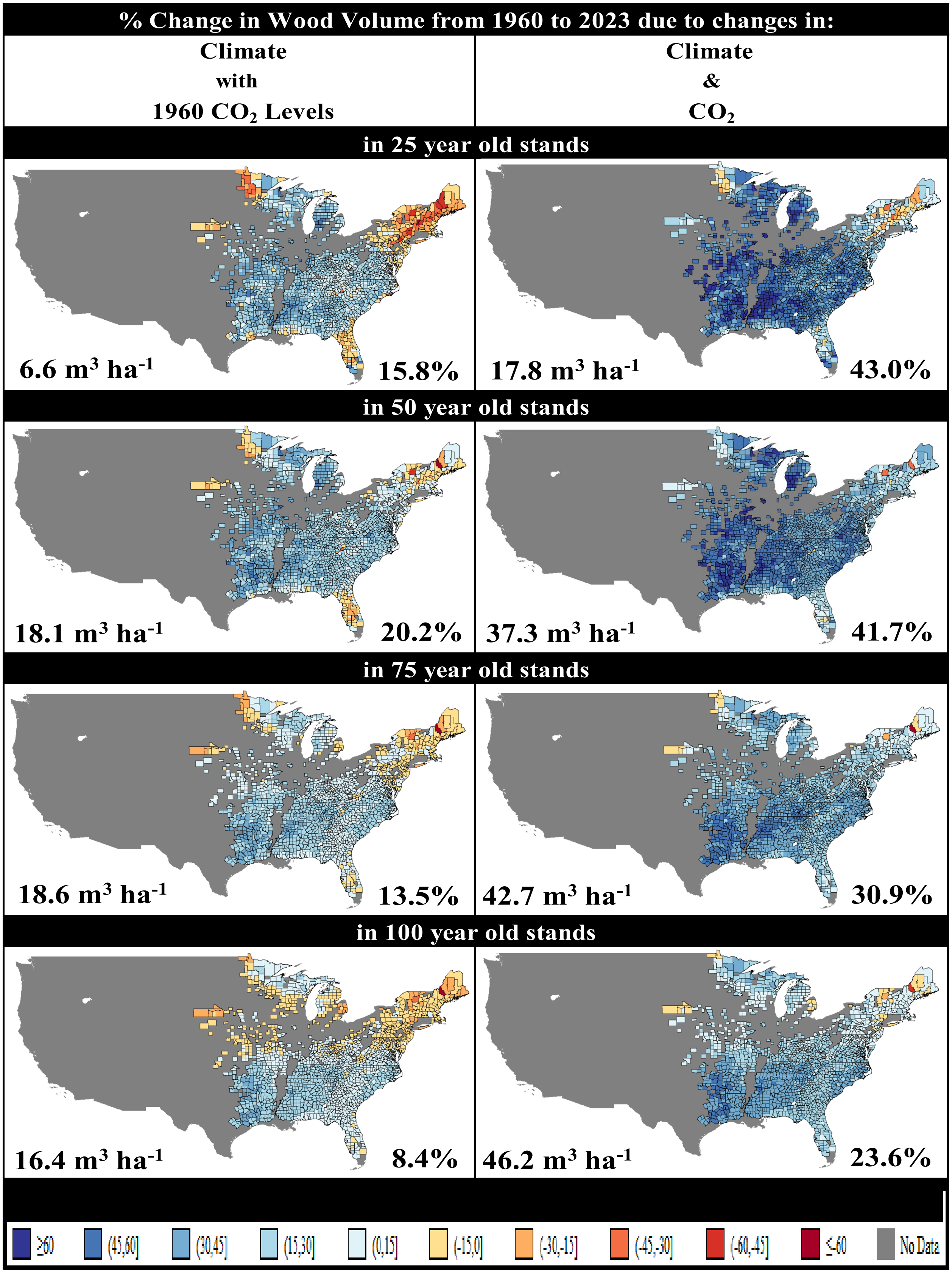


**Fig. S10. Estimated change in wood volume from 1960 to 2023, by county and age, based on observations of naturally regenerated Oak/Pine stands aged 1 to 150.** *Note:* The numerical values in the bottom left of each pane detail the mean county impact in cubic meters per hectare and the values in the bottom right of each pane detail the mean percentage change.

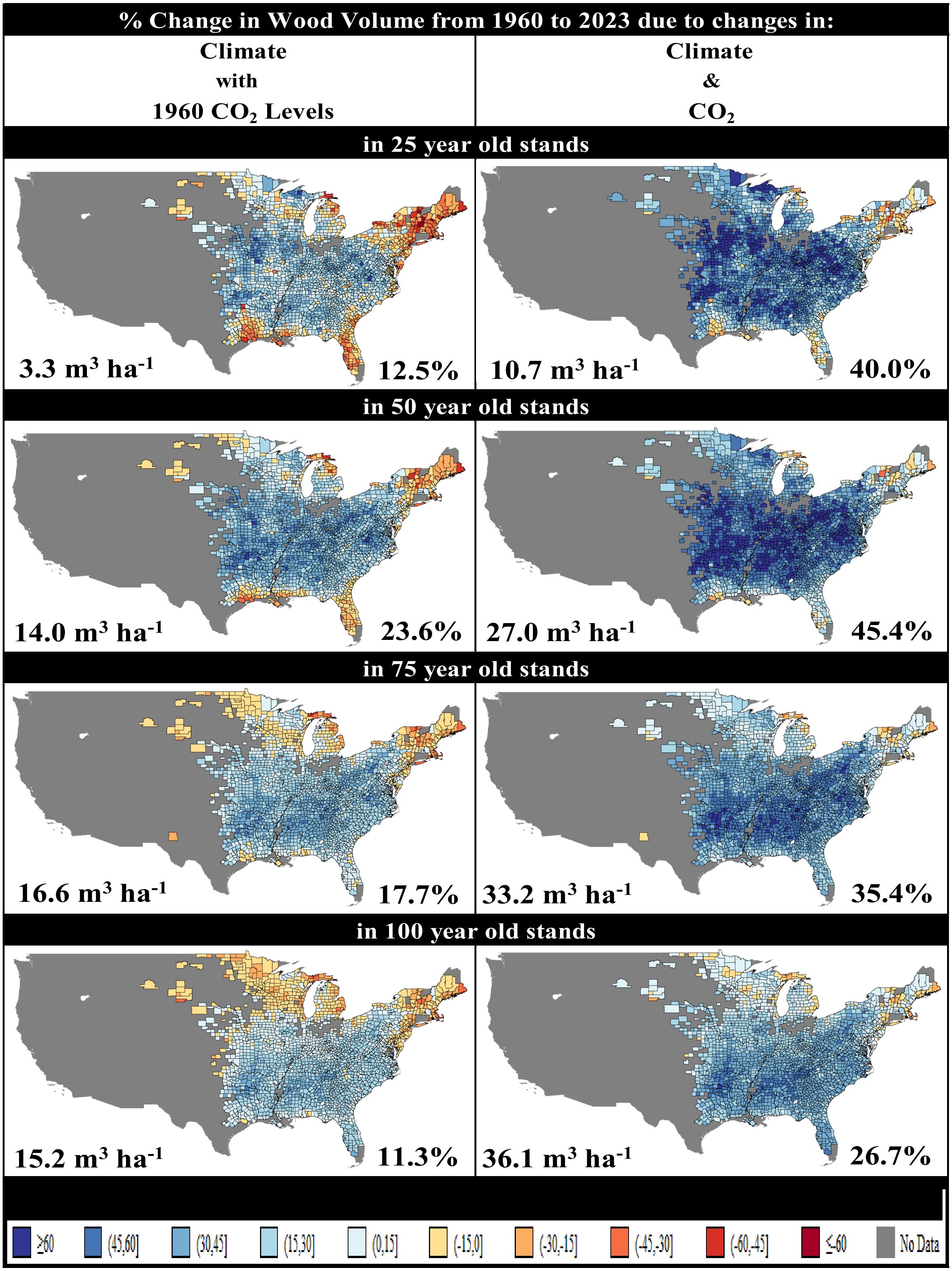


**Fig. S11. Estimated change in wood volume from 1960 to 2023, by county and age, based on observations of naturally regenerated Oak/Hickory stands aged 1 to 150.** *Note:* The numerical values in the bottom left of each pane detail the mean county impact in cubic meters per hectare and the values in the bottom right of each pane detail the mean percentage change.

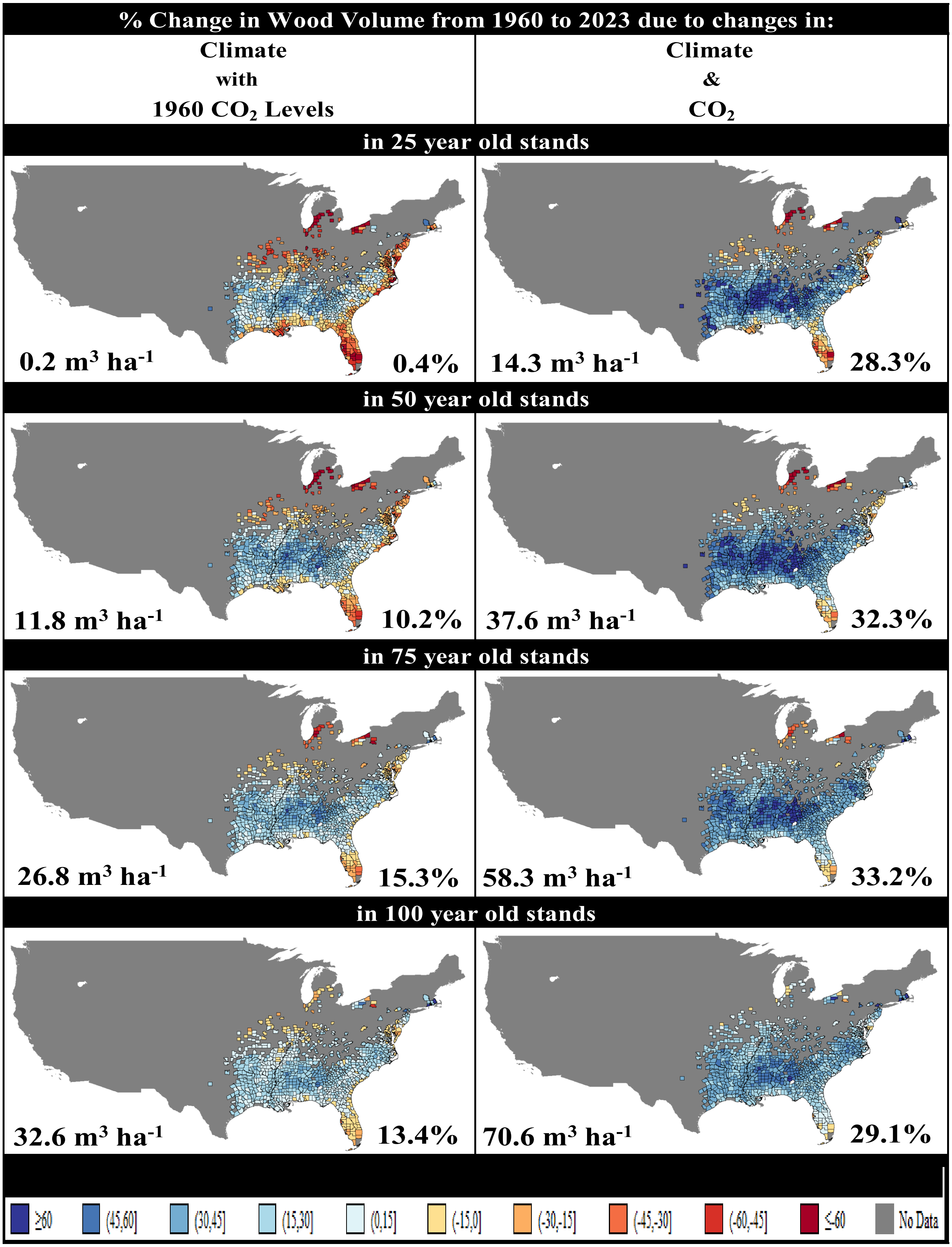


**Fig. S12. Estimated change in wood volume from 1960 to 2023, by county and age, based on observations of naturally regenerated Oak/Gum/Cypress stands aged 1 to 150.** *Note:* The numerical values in the bottom left of each pane detail the mean county impact in cubic meters per hectare and the values in the bottom right of each pane detail the mean percentage change.

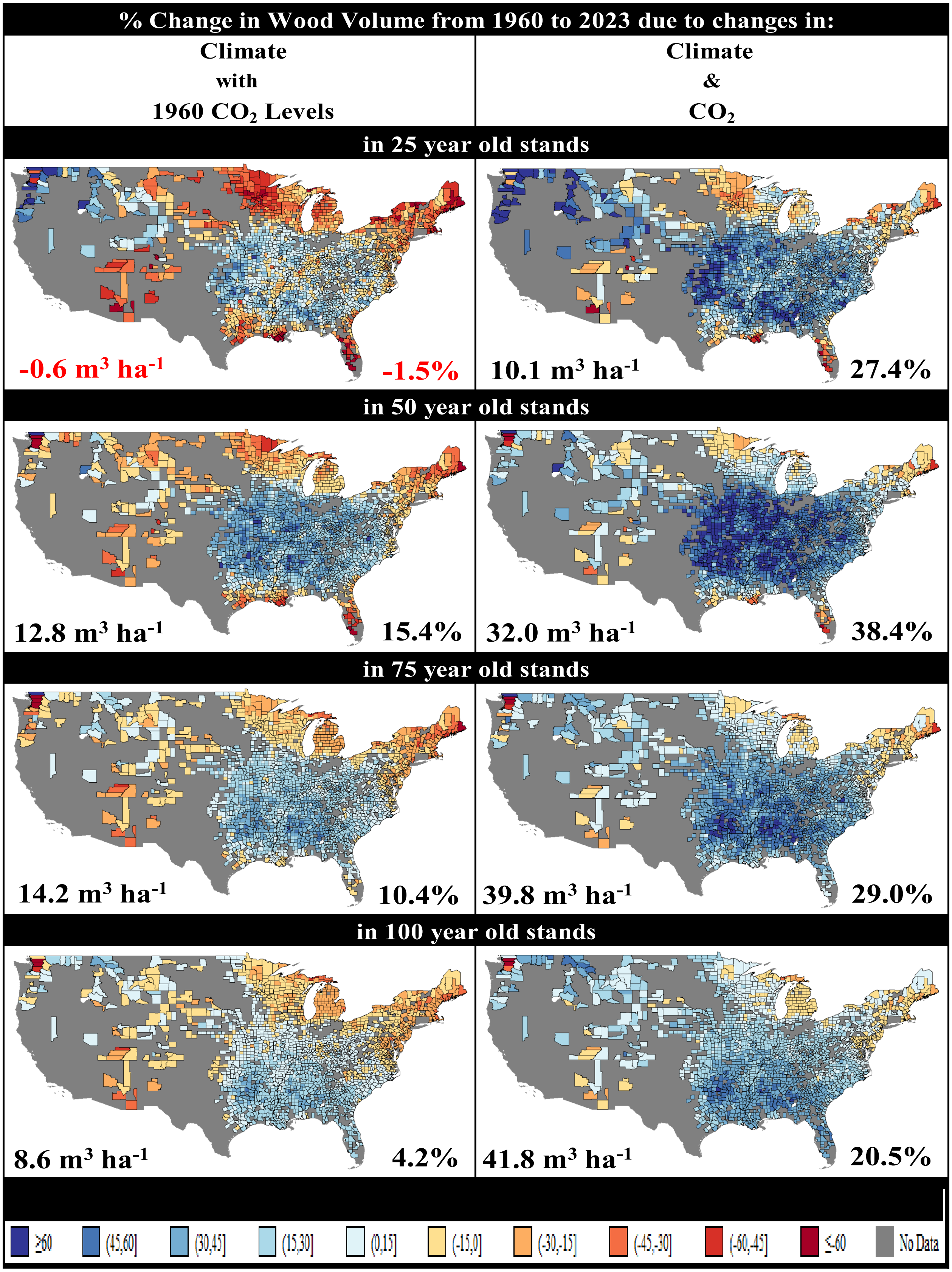


**Fig. S13. Estimated change in wood volume from 1960 to 2023, by county and age, based on observations of naturally regenerated Elm/Ash/Cottonwood stands aged 1 to 150.** *Note:* The numerical values in the bottom left of each pane detail the mean county impact in cubic meters per hectare and the values in the bottom right of each pane detail the mean percentage change.

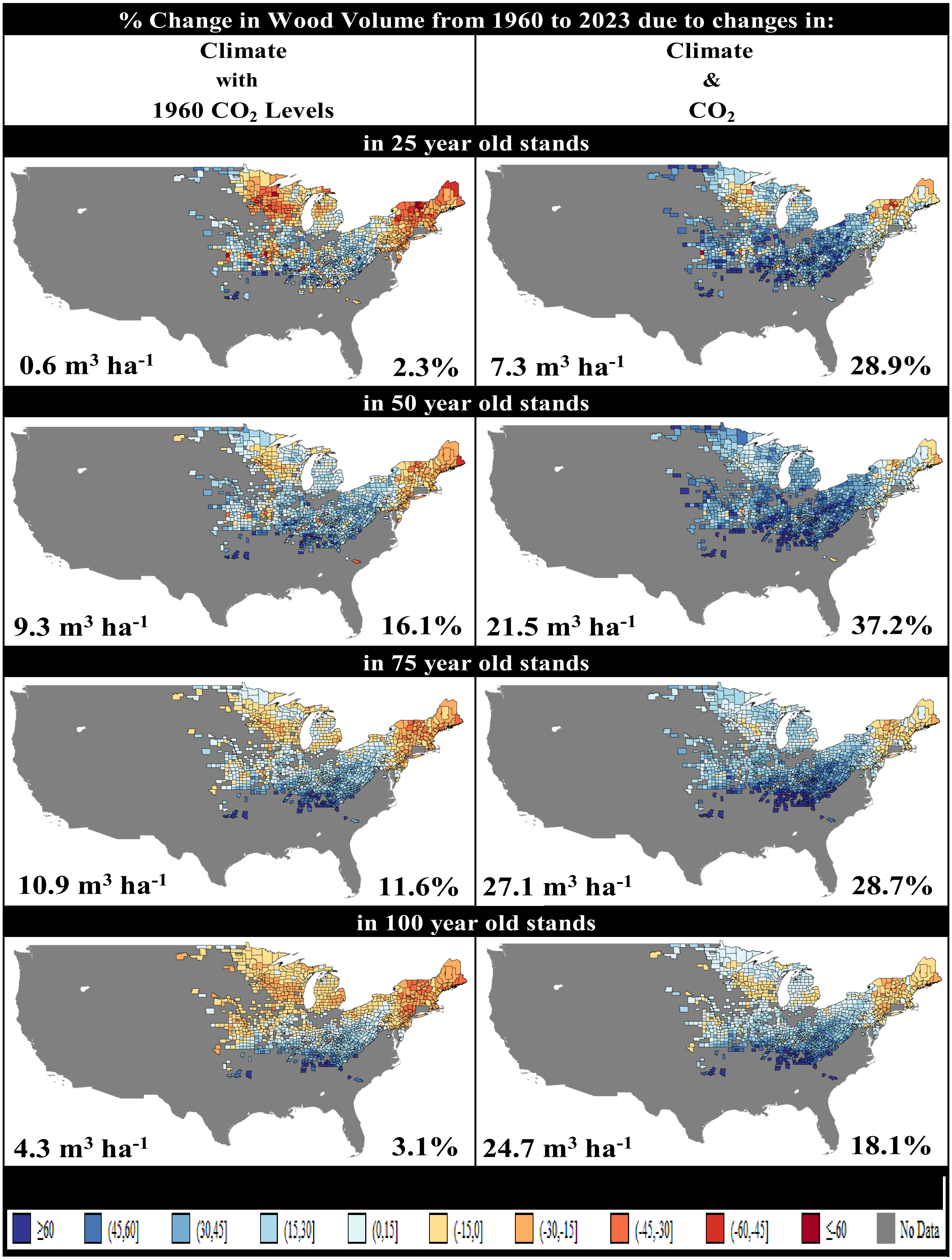


**Fig. S14. Estimated change in wood volume from 1960 to 2023, by county and age, based on observations of naturally regenerated Maple/Beech/Birch stands aged 1 to 150.** *Note:* The numerical values in the bottom left of each pane detail the mean county impact in cubic meters per hectare and the values in the bottom right of each pane detail the mean percentage change.

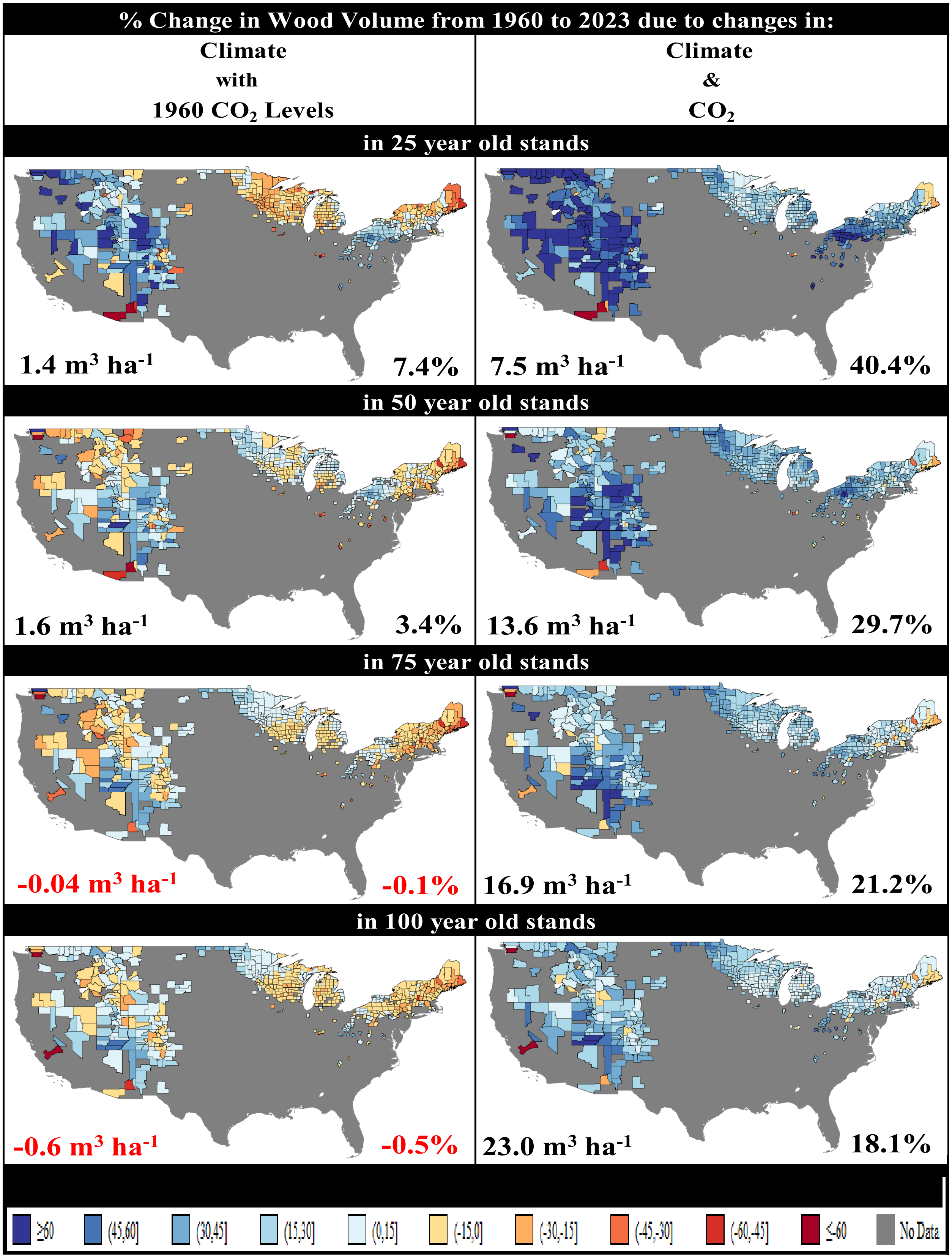


**Fig. S15. Estimated change in wood volume from 1960 to 2023, by county and age, based on observations of naturally regenerated Aspen/Birch stands aged 1 to 150.** *Note:* The numerical values in the bottom left of each pane detail the mean county impact in cubic meters per hectare and the values in the bottom right of each pane detail the mean percentage change.

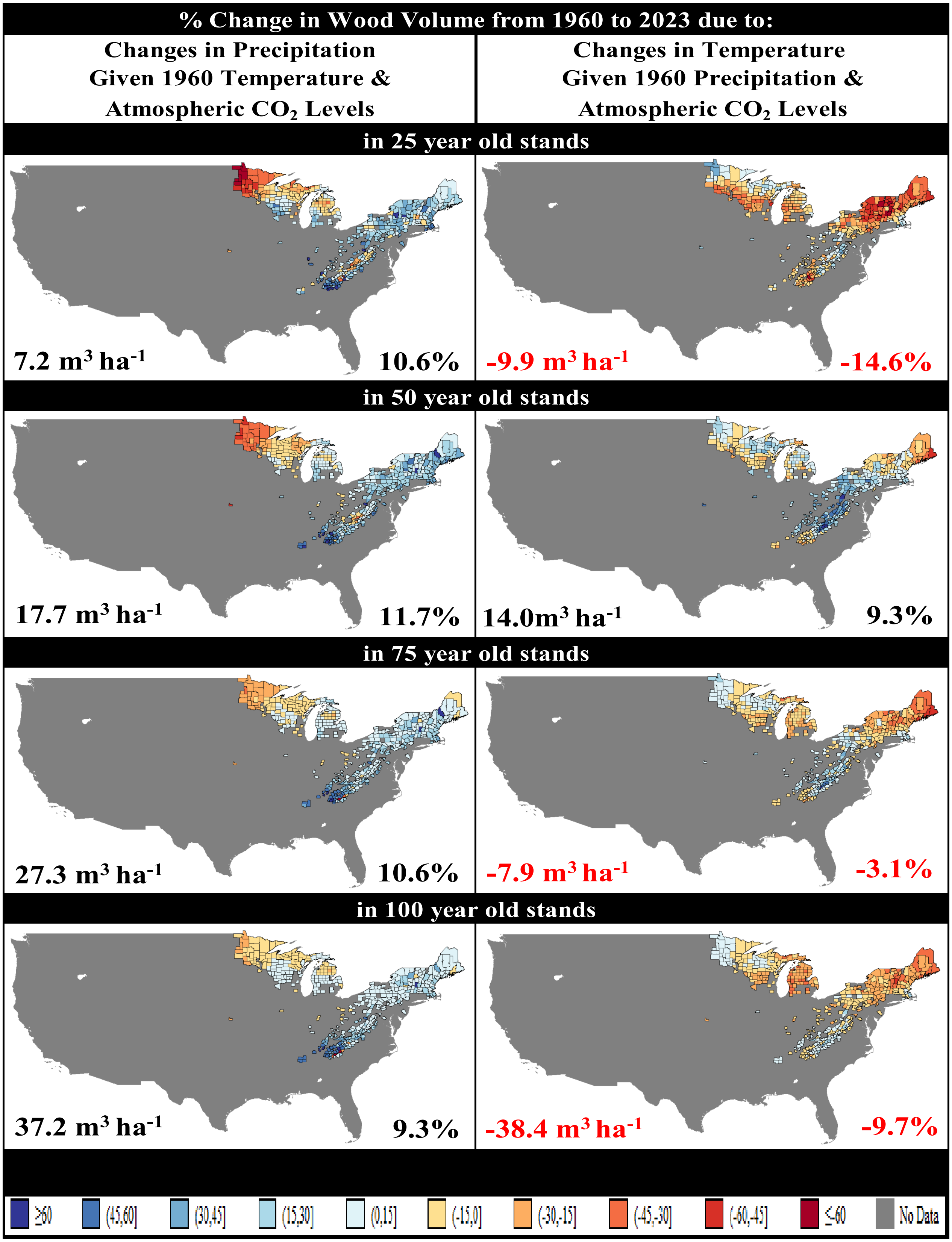


**Fig. S16. Estimated wood volume change from 1960 to 2023, by climate component, age, and county, based on observations of naturally regenerated White/Red/Jack stands aged 1 to 150.** *Note:* The numerical values in the bottom left of each pane detail the mean county impact in cubic meters per hectare and the values in the bottom right of each pane detail the mean percentage change.

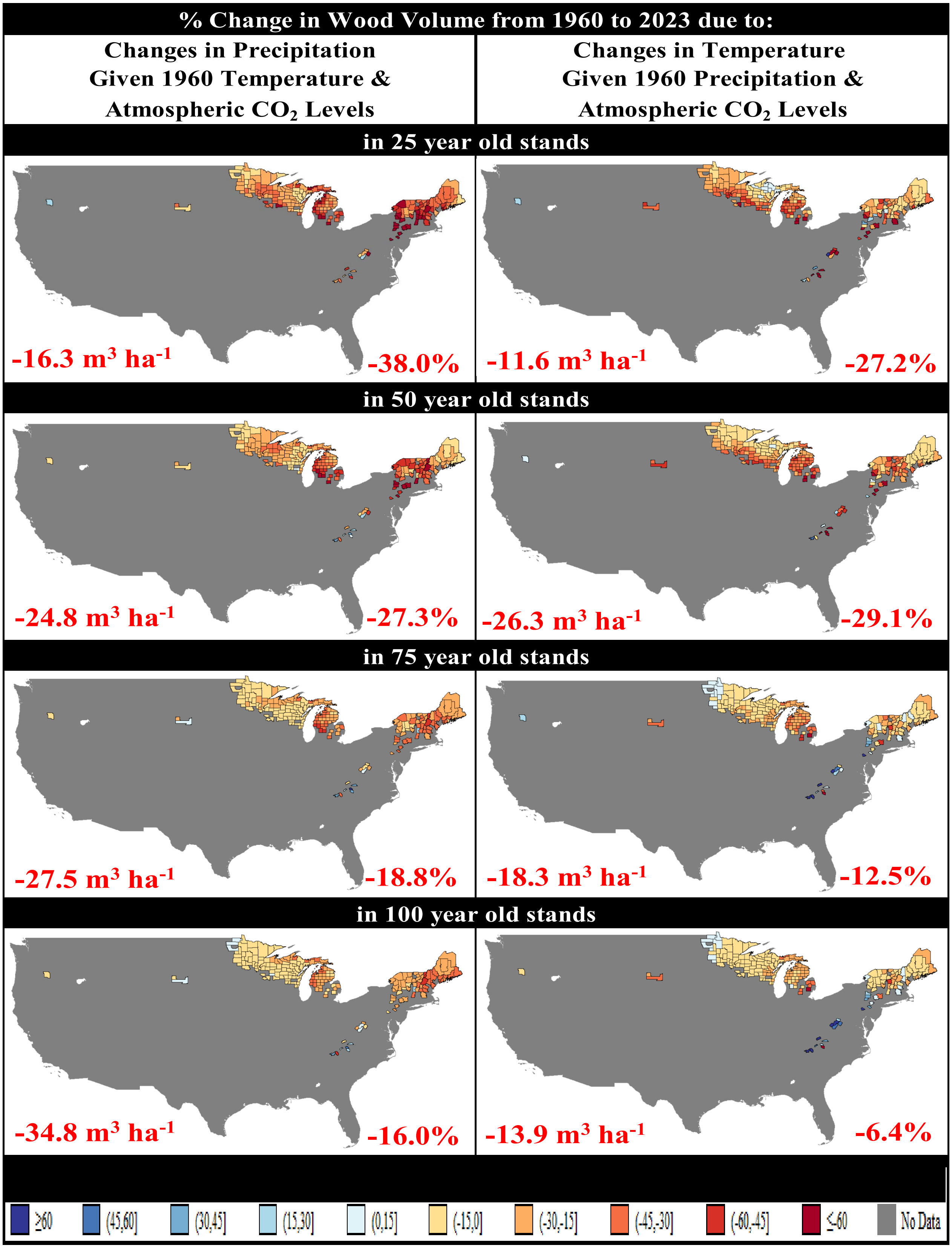


**Fig. S17. Estimated wood volume change from 1960 to 2023, by climate component, age, and county, based on observations of naturally regenerated Spruce/Fir stands aged 1 to 150.** *Note:* The numerical values in the bottom left of each pane detail the mean county impact in cubic meters per hectare and the values in the bottom right of each pane detail the mean percentage change.

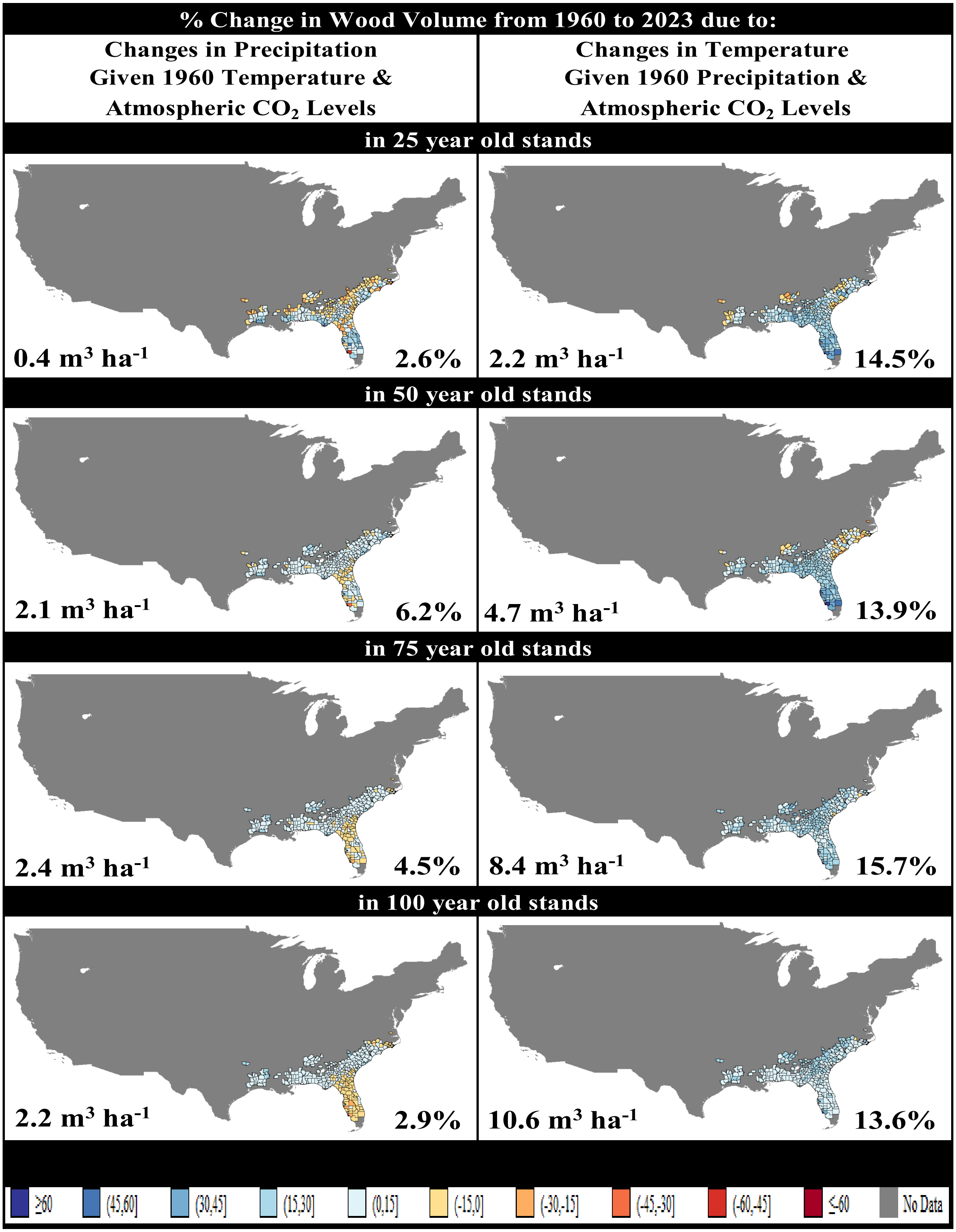


**Fig. S18. Estimated wood volume change from 1960 to 2023, by climate component, age, and county, based on observations of naturally regenerated Slash/Longleaf pine stands aged 1 to 150.** *Note:* The numerical values in the bottom left of each pane detail the mean county impact in cubic meters per hectare and the values in the bottom right of each pane detail the mean percentage change.

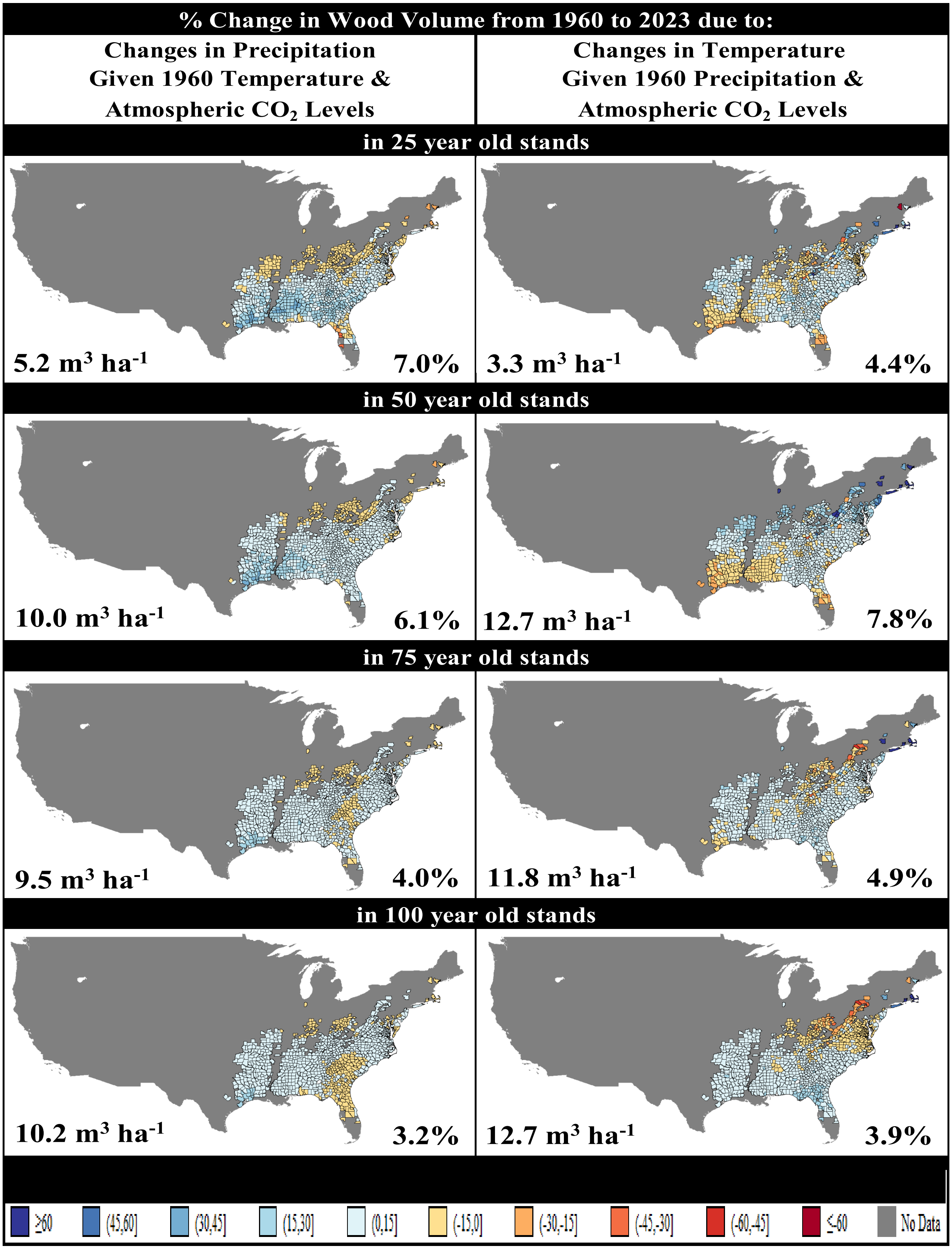


**Fig. S19. Estimated wood volume change from 1960 to 2023, by climate component, age, and county, based on observations of naturally regenerated Loblolly/Shortleaf stands aged 1 to 150.** *Note:* The numerical values in the bottom left of each pane detail the mean county impact in cubic meters per hectare and the values in the bottom right of each pane detail the mean percentage change.

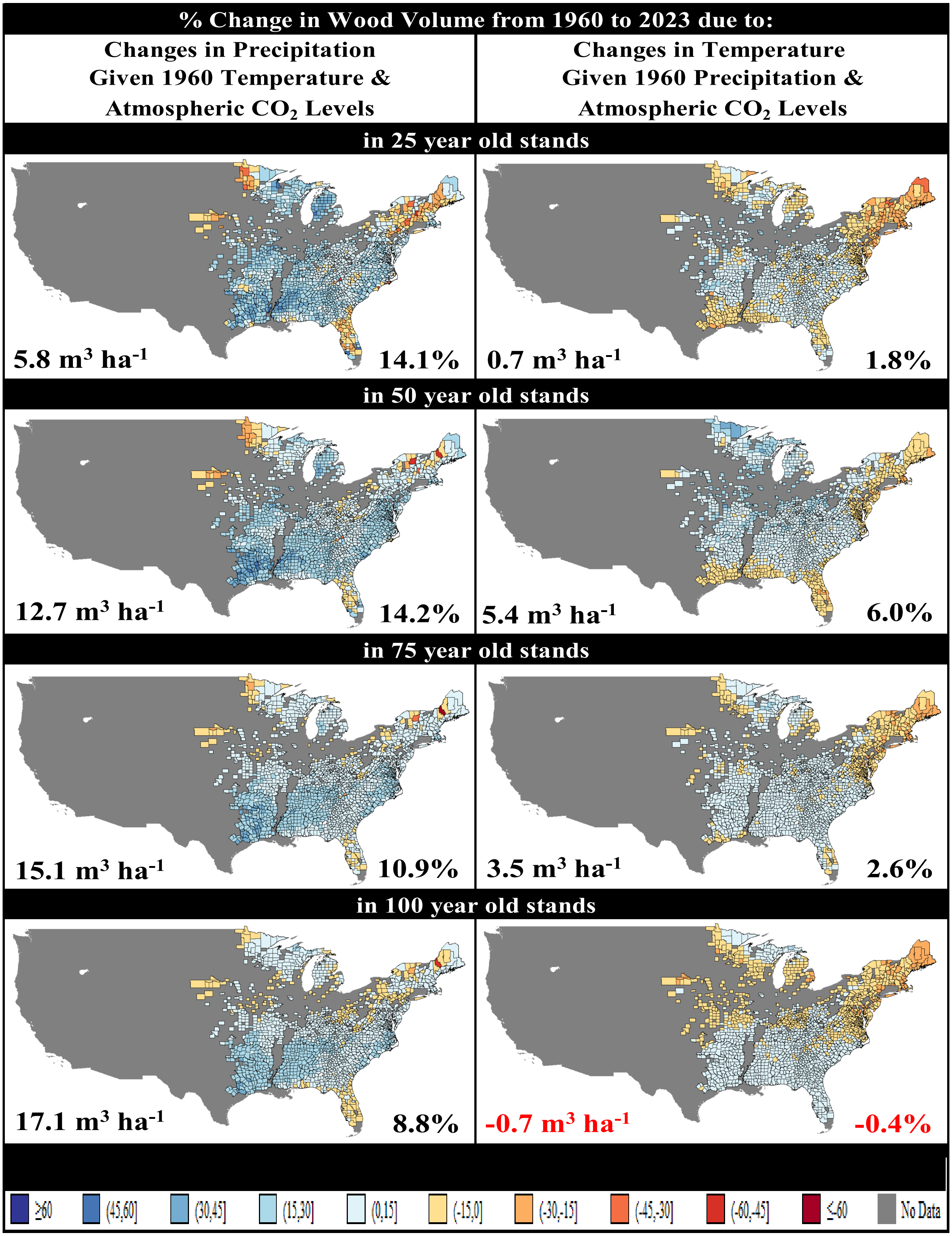


**Fig. S20. Estimated wood volume change from 1960 to 2023, by climate component, age, and county, based on observations of naturally regenerated Oak/Pine stands aged 1 to 150.** *Note:* The numerical values in the bottom left of each pane detail the mean county impact in cubic meters per hectare and the values in the bottom right of each pane detail the mean percentage change.

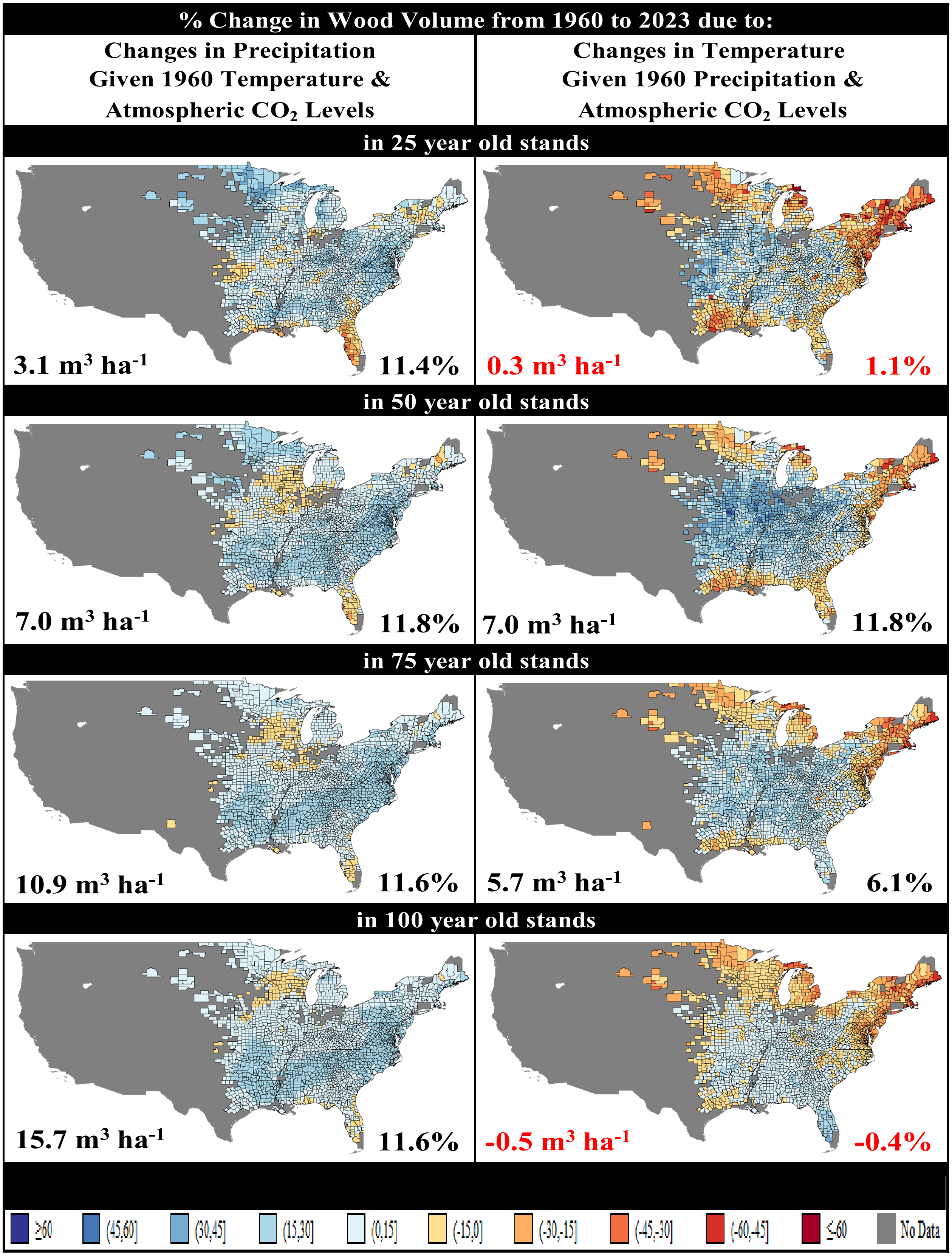


**Fig. S21. Estimated wood volume change from 1960 to 2023, by climate component, age, and county, based on observations of naturally regenerated Oak/Hickory stands aged 1 to 150.** *Note:* The numerical values in the bottom left of each pane detail the mean county impact in cubic meters per hectare and the values in the bottom right of each pane detail the mean percentage change.

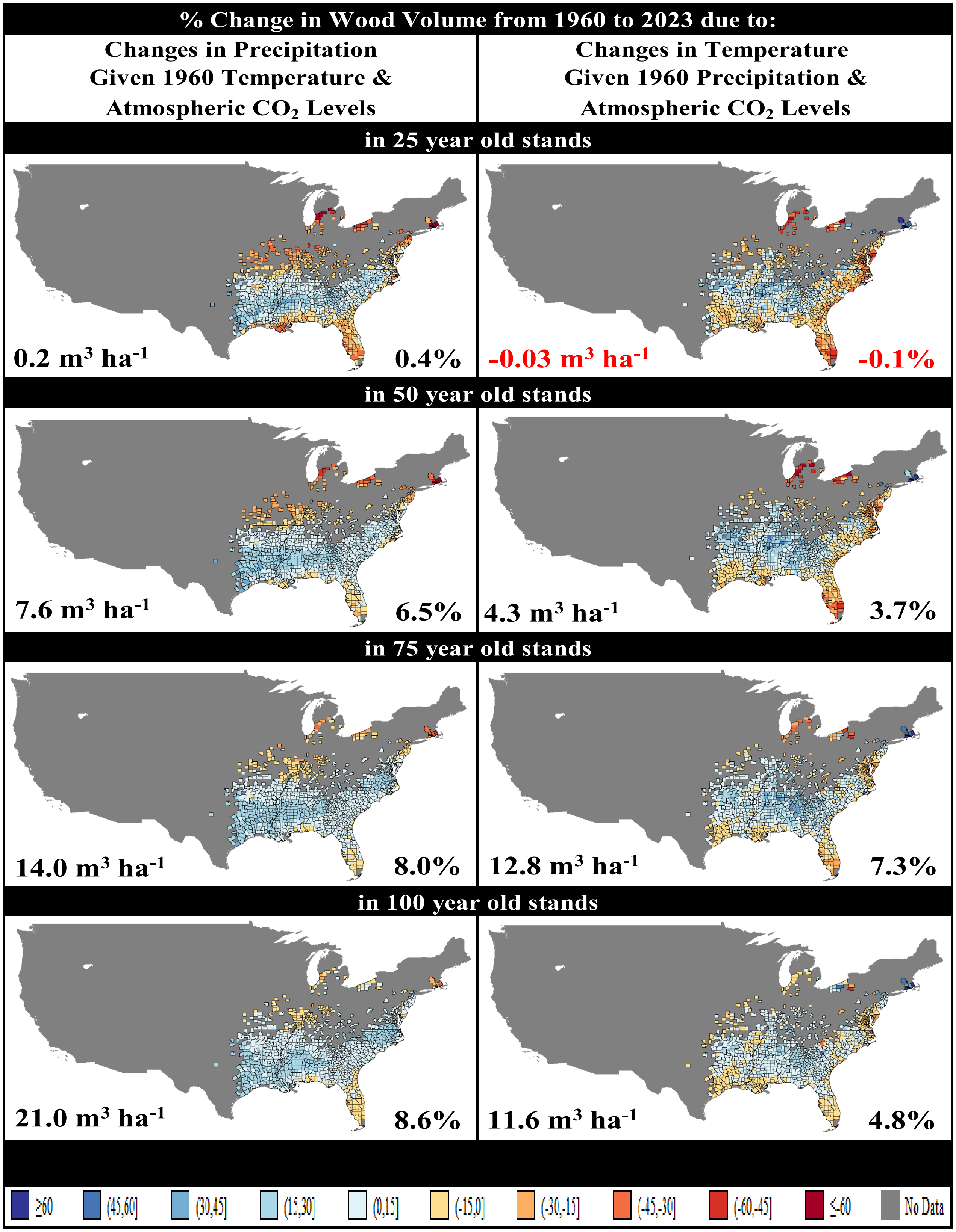


**Fig. S22. Estimated wood volume change from 1960 to 2023, by climate component, age, and county, based on observations of naturally regenerated Oak/Gum/Cypress stands aged 1 to 150.** *Note:* The numerical values in the bottom left of each pane detail the mean county impact in cubic meters per hectare and the values in the bottom right of each pane detail the mean percentage change.

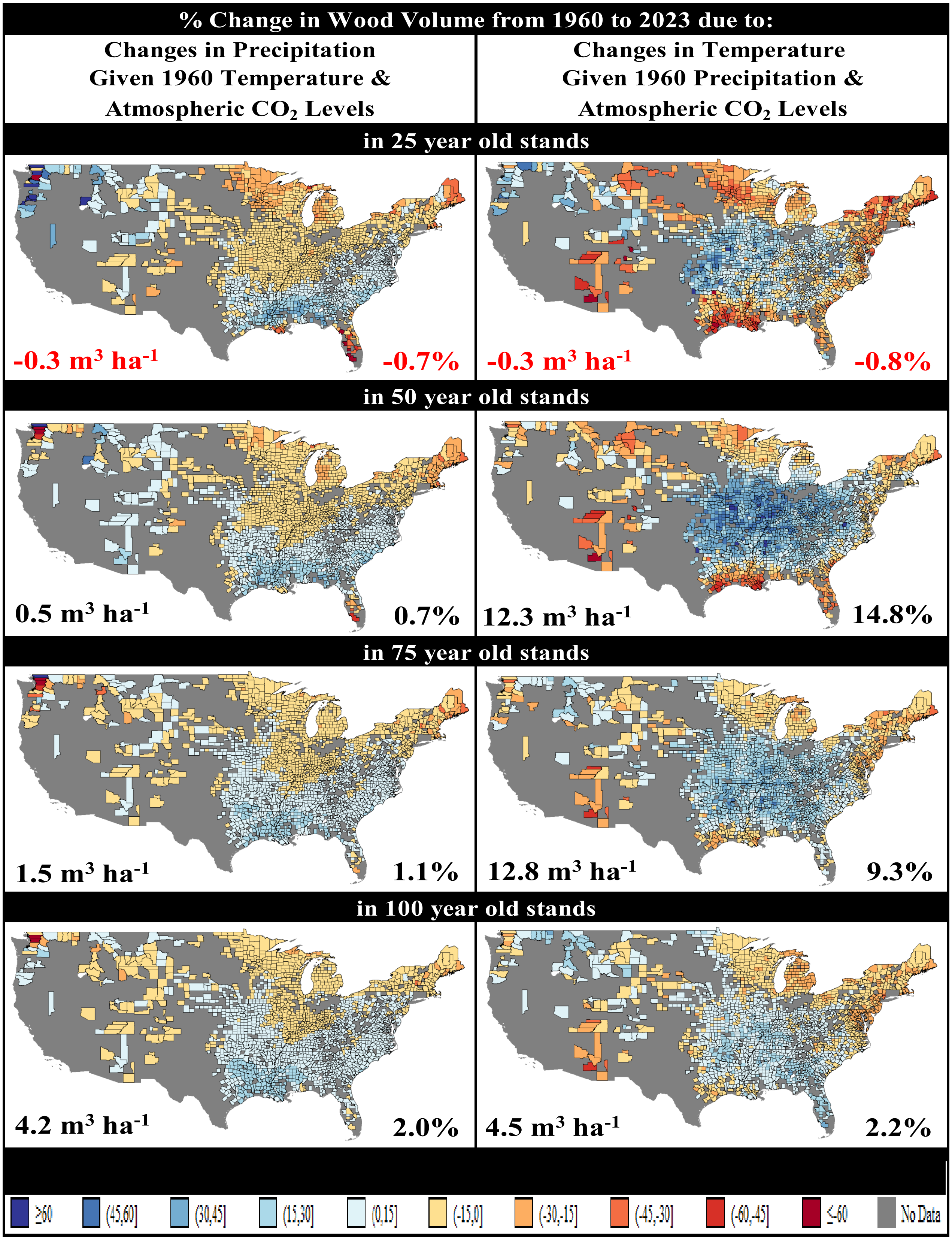


**Fig. S23. Estimated wood volume change from 1960 to 2023, by climate component, age, and county, based on observations of naturally regenerated Elm/Ash/Cottonwood stands aged 1-150.** *Note:* The numerical values in the bottom left of each pane detail the mean county impact in cubic meters per hectare and the values in the bottom right of each pane detail the mean percentage change.

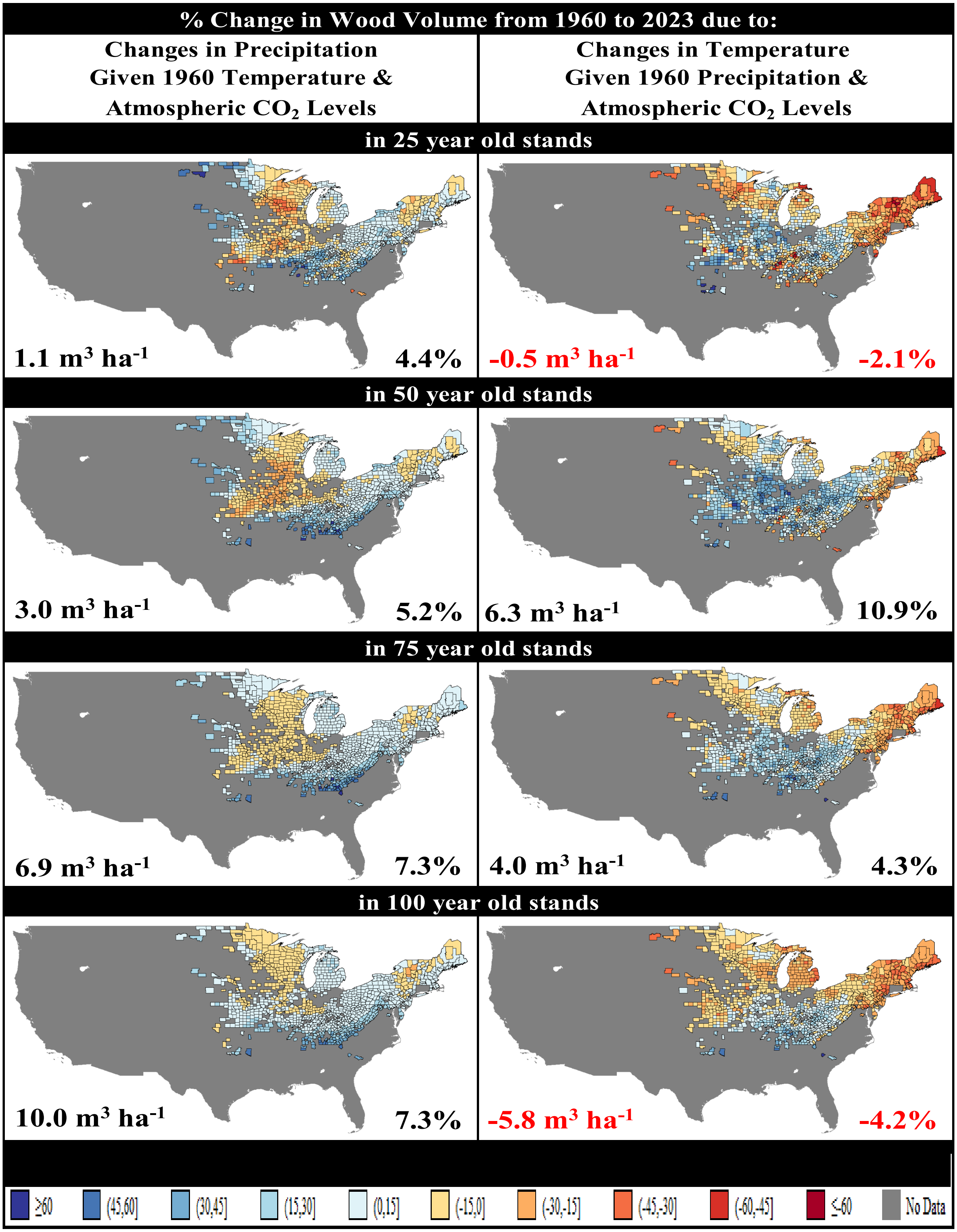


**Fig. S24. Estimated wood volume change from 1960 to 2023, by climate component, age, and county, based on observations of naturally regenerated Maple/Beech/Birch stands aged 1 to 150.** *Note:* The numerical values in the bottom left of each pane detail the mean county impact in cubic meters per hectare and the values in the bottom right of each pane detail the mean percentage change.

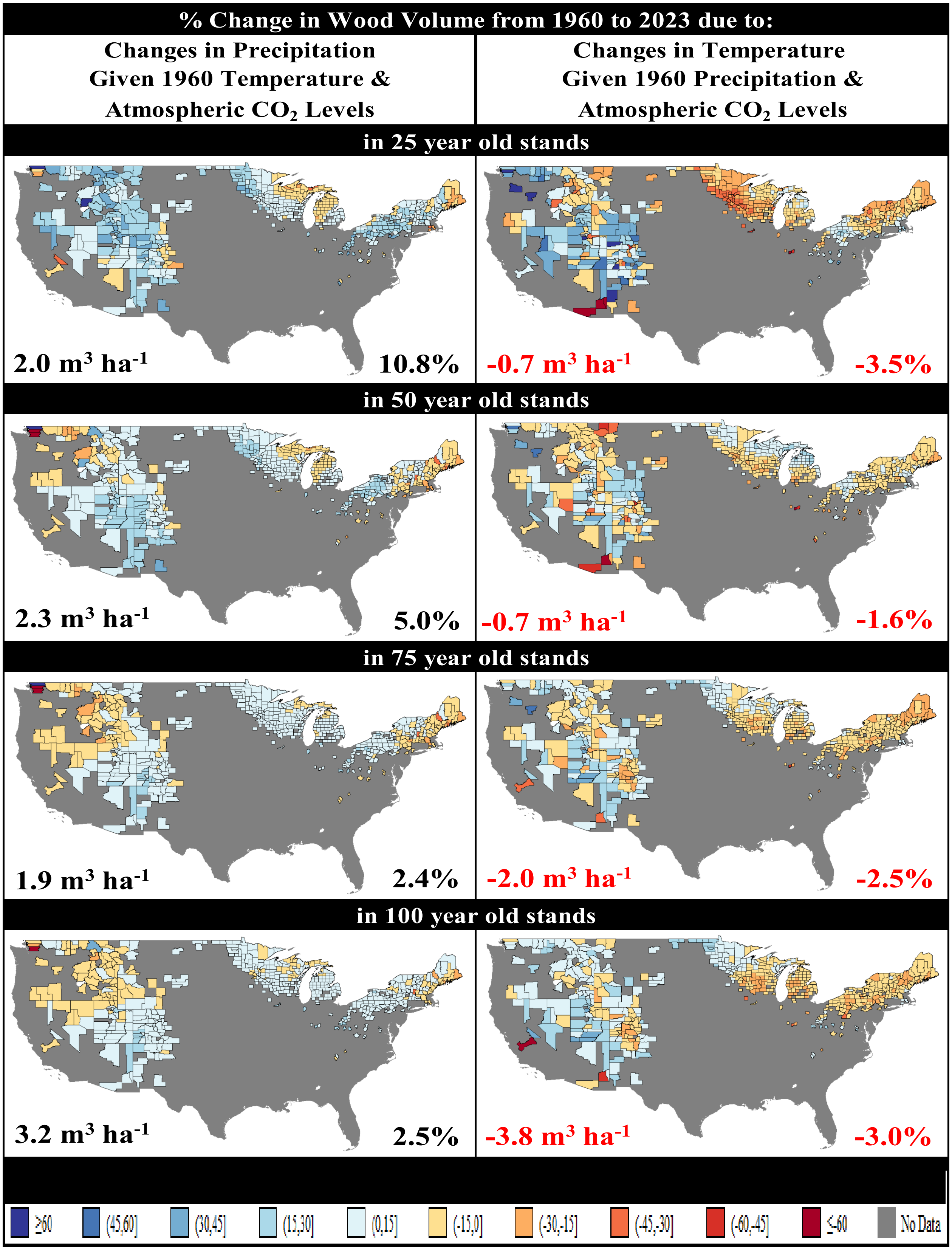


**Fig. S25. Estimated wood volume change from 1960 to 2023, by climate component, age, and county, based on observations of naturally regenerated Aspen/Birch stands aged 1 to 150.** *Note:* The numerical values in the bottom left of each pane detail the mean county impact in cubic meters per hectare and the values in the bottom right of each pane detail the mean percentage change.

**Table S4. Regression model raw output by forest group with interaction between seasonal temperature and precipitation and forest age as a robustness check. Data uses observations of naturally regenerated stands only, aged 1 to 150.**

| | Natural Log of Volume per Hectare | | | |
|---|---|---|---|---|
| Variables | Spruce/Fir | Oak/Pine | Oak/Hickory | Oak/Gum/Cypress |
| Natural Log of Lifetime $CO_2$ | 1.249*10^+00*** | 1.181*10^+00*** | 1.183*10^+00*** | 1.168*10^+00*** |
| 1/Age | -9.766*10^+00*** | 2.226*10^+01*** | 6.121*10^+00*** | -2.630*10^+01*** |
| Mean Spring Temperature | 2.754*10^-01 | 6.482*10^-01*** | 2.738*10^-01*** | -3.475*10^+00*** |
| Mean Spring Temperature Squared | 4.271*10^-02 | -4.613*10^-02*** | -4.410*10^-03 | 1.657*10^-01** |
| Mean Spring Temperature Cubed | -1.453*10^-02** | 8.133*10^-04** | -3.229*10^-04* | -2.706*10^-03* |
| Mean Summer Temperature | -3.821*10^+01*** | -1.261*10^+00 | -7.018*10^+00*** | 3.144*10^+01*** |
| Mean Summer Temperature Squared | 2.013*10^+00*** | 2.983*10^-02 | 2.990*10^-01*** | -1.223*10^+00*** |
| Mean Summer Temperature Cubed | -3.496*10^-02*** | -4.96*10^-05 | -4.332*10^-03*** | 1.551*10^-02*** |
| Mean Fall Temperature | 1.881*10^-01 | -7.533*10^-01*** | 7.408*10^-01*** | -1.607*10^+00 |
| Mean Fall Temperature Squared | -2.610*10^-02 | 5.227*10^-02*** | -7.630*10^-02*** | 8.130*10^-02 |
| Mean Fall Temperature Cubed | 1.105*10^-03 | -1.145*10^-03*** | 2.308*10^-03*** | -1.093*10^-03 |
| Mean Winter Temperature | 8.153*10^-02 | -2.180*10^-01*** | -2.772*10^-01*** | 1.441*10^-01* |
| Mean Winter Temperature Squared | 2.217*10^-02 | 4.541*10^-03*** | 7.812*10^-03*** | -1.294*10^-02 |
| Mean Winter Temperature Cubed | 4.293*10^-04 | 4.814*10^-04*** | 2.231*10^-04*** | 2.376*10^-04 |
| Mean Spring Precipitation | -5.598*10^-02* | -1.587*10^-02 | 2.022*10^-02*** | -1.028*10^-01*** |
| Mean Spring Precipitation Squared | 4.044*10^-04 | 9.12*10^-05 | -1.594*10^-04*** | 8.690*10^-04*** |
| Mean Spring Precipitation Cubed | -4.34*10^-07 | -2.58*10^-07 | 3.70*10^-07** | -2.45*10^-06*** |
| Mean Summer Precipitation | -1.854*10^-01*** | 3.426*10^-02*** | -1.080*10^-02** | 2.658*10^-02*** |
| Mean Summer Precipitation Squared | 1.945*10^-03*** | -2.265*10^-04*** | 3.24*10^-05 | -1.779*10^-04*** |
| Mean Summer Precipitation Cubed | -6.82*10^-06*** | 4.51*10^-07*** | -7.09*10^-08 | 3.32*10^-07*** |
| Mean Fall Precipitation | 7.729*10^-02*** | -5.242*10^-02*** | 3.306*10^-02*** | 5.988*10^-02*** |
| Mean Fall Precipitation Squared | -1.107*10^-03*** | 7.374*10^-04*** | -1.258*10^-04*** | -3.799*10^-04*** |
| Mean Fall Precipitation Cubed | 3.89*10^-06*** | -2.77*10^-06*** | 1.60*10^-07 | 8.25*10^-07*** |
| Mean Winter Precipitation | 1.102*10^-02 | 3.921*10^-02*** | -1.587*10^-02*** | 3.748*10^-02** |
| Mean Winter Precipitation Squared | -2.853*10^-04 | -2.368*10^-04*** | 1.167*10^-04*** | -2.947*10^-04* |
| Mean Winter Precipitation Cubed | 2.00*10^-06 | 5.58*10^-07*** | -1.77*10^-07* | 8.45*10^-07* |
| Spring Temperature-Age Interaction | 1.794*10^-01 | 2.481*10^-01* | -2.222*10^-01*** | -3.687*10^-01** |
| Summer Temperature-Age Interaction | 6.136*10^-01*** | -1.203*10^+00*** | 2.824*10^-01*** | 1.556*10^+00*** |
| Fall Temperature-Age Interaction | -2.072*10^-01 | 4.441*10^-01*** | -4.483*10^-01*** | 6.916*10^-02 |
| Winter Temperature-Age Interaction | -3.853*10^-01*** | 4.971*10^-02 | 1.414*10^-01*** | -6.082*10^-01*** |
| Spring Precipitation-Age Interaction | 6.670*10^-02*** | 3.721*10^-02*** | 9.185*10^-03*** | 2.834*10^-02*** |
| Summer Precipitation-Age Interaction | 4.815*10^-04 | -1.449*10^-03 | 1.218*10^-02*** | 5.67*10^-05 |
| Fall Precipitation-Age Interaction | -2.634*10^-02*** | -3.125*10^-02*** | -2.489*10^-02*** | -1.480*10^-02*** |
| Winter Precipitation-Age Interaction | -8.277*10^-03 | -1.874*10^-02*** | -6.766*10^-04 | -4.068*10^-02*** |
| Site Class 1 | | 6.274*10^-02 | 2.095*10^-01*** | 3.246*10^-01*** |
| Site Class 2 | 3.881*10^-01 | 6.917*10^-01*** | 4.392*10^-01*** | 3.787*10^-01*** |
| Site Class 3 | 5.966*10^-01*** | 6.475*10^-01*** | 4.374*10^-01*** | 3.850*10^-01*** |
| Site Class 4 | 5.024*10^-01*** | 4.819*10^-01*** | 3.394*10^-01*** | 3.297*10^-01*** |
| Site Class 5 | 4.448*10^-01*** | 2.945*10^-01*** | 1.745*10^-01*** | 2.317*10^-01*** |
| Site Class 6 | - | - | - | - |
| Time Dummy (2001 to 2023) | 3.084*10^-01*** | -9.459*10^-02*** | -1.700*10^-01*** | -2.210*10^-01*** |
| Time Dummy (1981 to 2000) | 1.878*10^-01*** | -6.900*10^-02*** | -1.390*10^-01*** | -1.121*10^-01*** |
| Time Dummy (≤1980) | - | - | - | - |
| Elevation | -1.071*10^-04*** | 4.24*10^-05*** | 4.74*10^-05*** | 1.295*10^-04*** |
| Slope | 2.161*10^-03*** | 3.011*10^-04 | 8.225*10^-04*** | -3.329*10^-03*** |
| Disturbances | -1.170*10^-03 | -4.227*10^-02*** | -1.646*10^-03 | -5.680*10^-02*** |
| Xeric Physiography | -3.515*10^-01*** | -2.019*10^-01*** | -2.014*10^-01*** | -5.292*10^-01*** |
| Hydric Physiography | -3.963*10^-01*** | 1.518*10^-02 | 4.980*10^-02*** | -2.766*10^-02*** |
| Mesic Physiography | - | - | - | - |
| North-facing Aspect | 3.086*10^-02** | 1.888*10^-02*** | 1.982*10^-02*** | 3.706*10^-02** |
| Null Aspect | -2.004*10^-01*** | 5.629*10^-03 | 1.639*10^-03 | -5.118*10^-02*** |
| South-facing Aspect | - | - | - | - |
| Private Land Ownership | 1.197*10^-01*** | -8.403*10^-03 | 1.463*10^-02*** | -3.340*10^-04 |
| Latitude | -6.502*10^-02*** | 3.290*10^-02* | -3.663*10^-03 | 1.194*10^-01*** |
| Longitude | 1.170*10^-01*** | -2.798*10^-02* | -1.730*10^-02** | 6.780*10^-03 |
| Stocking Density 1 | -3.962*10^-02 | 6.356*10^-01*** | 7.532*10^-01*** | 1.039*10^+00*** |
| Stocking Density 2 | 4.790*10^-01*** | 7.044*10^-01*** | 7.605*10^-01*** | 9.408*10^-01*** |
| Stocking Density 3 | 5.278*10^-01*** | 4.834*10^-01*** | 5.138*10^-01*** | 6.386*10^-01*** |
| Stocking Density 4 | 3.295*10^-01*** | 1.613*10^-01*** | 1.947*10^-01*** | 2.733*10^-01*** |
| Stocking Density 5 | - | - | - | - |
| Constant | 2.466*10^+02*** | 3.358*10^+00 | 4.402*10^+01*** | -2.417*10^+02*** |
| Observations | 64,867 | 97,667 | 471,966 | 88,241 |
| R-squared | 0.407 | 0.564 | 0.515 | 0.543 |

Note: All regressions employed county fixed effects. ***p<0.01//**p<0.05//*p<0.10

**Table S5. Regression model raw output by forest group with interaction between seasonal temperature and precipitation and forest age as a robustness check. Data uses observations of naturally regenerated stands only, aged 1 to 150.**

| | Natural Log of Volume per Hectare | | |
|---|---|---|---|
| Variables | Elm/Ash/Cottonwood | Maple/Beech/Birch | Aspen/Birch |
| Natural Log of Lifetime $CO_2$ | 1.245*10^+00*** | 1.126*10^+00*** | 1.425*10^+00*** |
| 1/Age | -6.722*10^+00*** | -2.068*10^+00* | 1.057*10^+01*** |
| Mean Spring Temperature | -4.421*10^-01*** | 8.821*10^-02 | -2.706*10^-01*** |
| Mean Spring Temperature Squared | 6.772*10^-02*** | 3.797*10^-02*** | 1.163*10^-01*** |
| Mean Spring Temperature Cubed | -2.296*10^-03*** | -2.917*10^-03*** | -6.678*10^-03*** |
| Mean Summer Temperature | 5.831*10^-01 | -7.909*10^+00*** | -1.813*10^+00** |
| Mean Summer Temperature Squared | 1.307*10^-02 | 3.769*10^-01*** | 1.242*10^-01*** |
| Mean Summer Temperature Cubed | -7.574*10^-04 | -6.095*10^-03*** | -2.457*10^-03*** |
| Mean Fall Temperature | 1.369*10^+00*** | -5.456*10^-02 | 9.643*10^-01*** |
| Mean Fall Temperature Squared | -1.327*10^-01*** | -1.792*10^-02 | -1.606*10^-01*** |
| Mean Fall Temperature Cubed | 3.450*10^-03*** | 1.354*10^-03* | 7.932*10^-03*** |
| Mean Winter Temperature | -3.291*10^-01*** | -3.902*10^-01*** | -3.068*10^-01*** |
| Mean Winter Temperature Squared | 1.381*10^-02*** | 2.118*10^-02*** | 1.966*10^-02*** |
| Mean Winter Temperature Cubed | 6.680*10^-04*** | 1.509*10^-03*** | 1.070*10^-03*** |
| Mean Spring Precipitation | -1.605*10^-02 | 1.496*10^-01*** | 5.171*10^-03 |
| Mean Spring Precipitation Squared | 1.665*10^-04* | -1.823*10^-03*** | -5.52*10^-05 |
| Mean Spring Precipitation Cubed | -6.61*10^-07** | 6.80*10^-06*** | -3.62*10^-09 |
| Mean Summer Precipitation | -3.479*10^-02*** | 7.801*10^-02*** | -3.877*10^-02*** |
| Mean Summer Precipitation Squared | 2.621*10^-04*** | -5.955*10^-04*** | 3.663*10^-04*** |
| Mean Summer Precipitation Cubed | -6.24*10^-07*** | 1.31*10^-06** | -9.03*10^-07*** |
| Mean Fall Precipitation | -2.725*10^-02*** | 1.213*10^-02 | 2.078*10^-02*** |
| Mean Fall Precipitation Squared | 2.608*10^-04*** | -8.12*10^-05 | -4.64*10^-05 |
| Mean Fall Precipitation Cubed | -5.85*10^-07*** | 3.17*10^-07 | -2.50*10^-07* |
| Mean Winter Precipitation | -2.869*10^-02*** | -3.933*10^-02*** | -6.924*10^-03** |
| Mean Winter Precipitation Squared | 2.486*10^-04*** | 4.756*10^-04*** | -5.88*10^-06 |
| Mean Winter Precipitation Cubed | -5.92*10^-07*** | -1.41*10^-06*** | -1.16*10^-07 |
| Spring Temperature-Age Interaction | -2.977*10^-01*** | -1.645*10^-02 | -4.516*10^-01*** |
| Summer Temperature-Age Interaction | 8.652*10^-01*** | 4.865*10^-01*** | 2.718*10^-01*** |
| Fall Temperature-Age Interaction | -5.427*10^-01*** | -4.131*10^-01*** | -3.321*10^-01*** |
| Winter Temperature-Age Interaction | -3.300*10^-02 | -4.656*10^-02 | 3.119*10^-01*** |
| Spring Precipitation-Age Interaction | 1.281*10^-02*** | -1.038*10^-02** | -1.383*10^-03 |
| Summer Precipitation-Age Interaction | 1.062*10^-02*** | 2.109*10^-02*** | 2.967*10^-03 |
| Fall Precipitation-Age Interaction | -2.312*10^-02*** | 2.171*10^-03 | -2.795*10^-02*** |
| Winter Precipitation-Age Interaction | 1.432*10^-02*** | -2.362*10^-02*** | -1.509*10^-02*** |
| Site Class 1 | 6.449*10^-01*** | 1.145*10^-01 | 5.376*10^-01*** |
| Site Class 2 | 4.592*10^-01*** | 3.691*10^-01*** | 6.349*10^-01*** |
| Site Class 3 | 4.289*10^-01*** | 1.598*10^-01*** | 5.972*10^-01*** |
| Site Class 4 | 3.443*10^-01*** | 1.627*10^-01*** | 4.504*10^-01*** |
| Site Class 5 | 2.213*10^-01*** | 1.053*10^-01*** | 2.185*10^-01*** |
| Site Class 6 | - | - | - |
| Time Dummy (2001 to 2023) | -1.230*10^-01*** | 6.061*10^-02*** | -3.686*10^-01*** |
| Time Dummy (1981 to 2000) | -5.800*10^-02** | -4.935*10^-02*** | 6.532*10^-02*** |
| Time Dummy (≤1980) | - | - | - |
| Elevation | -2.90*10^-05** | -4.10*10^-05*** | 8.98*10^-05*** |
| Slope | -1.544*10^-03*** | 1.97*10^-05 | 1.526*10^-03*** |
| Disturbances | 7.071*10^-03 | -1.317*10^-02*** | 1.885*10^-02* |
| Xeric Physiography | -3.636*10^-01*** | -3.341*10^-01*** | -9.054*10^-02*** |
| Hydric Physiography | -1.870*10^-01*** | -1.915*10^-01*** | -4.028*10^-01*** |
| Mesic Physiography | - | - | - |
| North-facing Aspect | 2.530*10^-02** | 1.795*10^-04 | 2.288*10^-02*** |
| Null Aspect | -3.655*10^-02*** | -7.025*10^-02*** | -7.291*10^-02*** |
| South-facing Aspect | - | - | - |
| Private Land Ownership | 1.603*10^-02** | -5.304*10^-02*** | 1.047*10^-02* |
| Latitude | 3.761*10^-02** | 4.912*10^-03 | -2.466*10^-02** |
| Longitude | -5.767*10^-02*** | -6.962*10^-03 | 2.709*10^-02*** |
| Stocking Density 1 | 7.827*10^-01*** | 7.442*10^-01*** | 4.779*10^-01*** |
| Stocking Density 2 | 7.498*10^-01*** | 7.705*10^-01*** | 8.925*10^-01*** |
| Stocking Density 3 | 5.185*10^-01*** | 4.787*10^-01*** | 7.346*10^-01*** |
| Stocking Density 4 | 1.729*10^-01*** | 1.243*10^-01*** | 3.316*10^-01*** |
| Stocking Density 5 | - | - | - |
| Constant | -2.160*10^+01*** | 4.541*10^+01*** | -3.950*10^+00 |
| Observations | 101,220 | 162,130 | 110,632 |
| R-squared | 0.525 | 0.506 | 0.522 |

Note: All regressions employed county fixed effects. ***p<0.01//**p<0.05//*p<0.10

**Table S6. Regression model raw output by forest group with interaction between seasonal temperature and precipitation and forest age as a robustness check. Data uses observations of naturally regenerated stands only, aged 1 to 150.**

| | **Natural Log of Volume per Hectare** | | |
|---|---|---|---|
| Variables | White/Red/Jack | Slash/Longleaf | Loblolly/Shortleaf |
| Natural Log of Lifetime $CO_2$ | 1.220*10^+00*** | 1.236*10^+00*** | 1.091*10^+00*** |
| 1/Age | -5.952*10^+01*** | 1.365*10^+01 | 2.567*10^+01*** |
| Mean Spring Temperature | 1.641*10^+00*** | 1.351*10^+01** | 4.244*10^+00*** |
| Mean Spring Temperature Squared | -1.401*10^-01*** | -7.477*10^-01** | -2.328*10^-01*** |
| Mean Spring Temperature Cubed | 3.813*10^-03*** | 1.333*10^-02*** | 3.701*10^-03*** |
| Mean Summer Temperature | -2.751*10^+01*** | -6.138*10^+01 | 1.198*10^+01*** |
| Mean Summer Temperature Squared | 1.263*10^+00*** | 2.224*10^+00 | -4.528*10^-01*** |
| Mean Summer Temperature Cubed | -1.875*10^-02*** | -2.683*10^-02 | 5.875*10^-03*** |
| Mean Fall Temperature | 2.000*10^+00*** | -8.389*10^+00* | -2.285*10^+00** |
| Mean Fall Temperature Squared | -2.066*10^-01*** | 3.752*10^-01 | 1.133*10^-01* |
| Mean Fall Temperature Cubed | 5.432*10^-03*** | -5.181*10^-03 | -1.864*10^-03 |
| Mean Winter Temperature | -4.433*10^-01*** | 8.716*10^-01* | -3.533*10^-01*** |
| Mean Winter Temperature Squared | 4.002*10^-02*** | -7.862*10^-02** | 2.853*10^-02*** |
| Mean Winter Temperature Cubed | 2.388*10^-03*** | 1.710*10^-03 | -3.843*10^-04 |
| Mean Spring Precipitation | -1.283*10^-01*** | -1.299*10^-01*** | -2.496*10^-02** |
| Mean Spring Precipitation Squared | 1.183*10^-03*** | 1.035*10^-03*** | 1.307*10^-04 |
| Mean Spring Precipitation Cubed | -3.09*10^-06*** | -2.80*10^-06*** | -2.66*10^-07 |
| Mean Summer Precipitation | 1.628*10^-01*** | -1.163*10^-01*** | 2.027*10^-03 |
| Mean Summer Precipitation Squared | -1.544*10^-03*** | 6.814*10^-04*** | 3.95*10^-05 |
| Mean Summer Precipitation Cubed | 4.96*10^-06*** | -1.28*10^-06*** | -9.97*10^-08 |
| Mean Fall Precipitation | -1.399*10^-01*** | -3.270*10^-02 | -4.809*10^-03 |
| Mean Fall Precipitation Squared | 1.360*10^-03*** | 5.028*10^-04** | 7.26*10^-05 |
| Mean Fall Precipitation Cubed | -4.15*10^-06*** | -1.82*10^-06** | -1.67*10^-07 |
| Mean Winter Precipitation | 1.604*10^-02* | -2.364*10^-02 | 6.253*10^-02*** |
| Mean Winter Precipitation Squared | -1.834*10^-04 | 2.944*10^-04 | -5.344*10^-04*** |
| Mean Winter Precipitation Cubed | 2.60*10^-07 | -1.21*10^-06** | 1.63*10^-06*** |
| Spring Temperature-Age Interaction | -1.808*10^+00*** | -5.390*10^-01 | 9.087*10^-01*** |
| Summer Temperature-Age Interaction | 3.034*10^+00*** | -1.602*10^-01 | -1.829*10^+00*** |
| Fall Temperature-Age Interaction | -4.374*10^-01 | -5.227*10^-01 | 1.177*10^+00*** |
| Winter Temperature-Age Interaction | -3.658*10^-01*** | 9.831*10^-01*** | -7.143*10^-01*** |
| Spring Precipitation-Age Interaction | 7.945*10^-02*** | 5.485*10^-02*** | 1.801*10^-02*** |
| Summer Precipitation-Age Interaction | 1.113*10^-01*** | 6.377*10^-03 | -5.949*10^-02*** |
| Fall Precipitation-Age Interaction | 2.959*10^-02* | -2.348*10^-02*** | -1.283*10^-02*** |
| Winter Precipitation-Age Interaction | 2.369*10^-02 | -6.122*10^-03 | -2.181*10^-02*** |
| Site Class 1 | 4.386*10^-01*** | 6.512*10^-01*** | 2.065*10^-01*** |
| Site Class 2 | 7.108*10^-01*** | 4.600*10^-01*** | 8.222*10^-01*** |
| Site Class 3 | 5.950*10^-01*** | 7.681*10^-01*** | 7.319*10^-01*** |
| Site Class 4 | 4.301*10^-01*** | 6.219*10^-01*** | 5.458*10^-01*** |
| Site Class 5 | 2.694*10^-01*** | 3.821*10^-01*** | 3.313*10^-01*** |
| Site Class 6 | - | - | - |
| Time Dummy (2001 to 2023) | 2.418*10^-01*** | -2.213*10^-01*** | -4.779*10^-02*** |
| Time Dummy (1981 to 2000) | -7.025*10^-02** | -5.227*10^-02** | -7.111*10^-03 |
| Time Dummy (≤1980) | - | - | - |
| Elevation | 2.19*10^-05 | -2.91*10^-05 | -3.30*10^-05** |
| Slope | 2.035*10^-03*** | 4.528*10^-03*** | -1.392*10^-03*** |
| Disturbances | -4.369*10^-04 | -1.033*10^-02 | 2.260*10^-02*** |
| Xeric Physiography | -1.632*10^-01*** | -9.208*10^-02*** | -1.485*10^-01*** |
| Hydric Physiography | -3.940*10^-01*** | -2.766*10^-02* | -1.856*10^-01*** |
| Mesic Physiography | - | - | - |
| North-facing Aspect | -6.222*10^-03 | 4.935*10^-02** | 1.438*10^-02** |
| Null Aspect | 8.931*10^-03 | -4.202*10^-02** | 2.917*10^-02*** |
| South-facing Aspect | - | - | - |
| Private Land Ownership | 3.108*10^-02*** | 7.129*10^-02*** | -4.627*10^-03 |
| Latitude | -2.209*10^-01*** | 2.903*10^-01*** | 5.699*10^-02*** |
| Longitude | -1.234*10^-01*** | 2.024*10^-02 | -1.745*10^-02 |
| Stocking Density 1 | 1.130*10^+00*** | 1.726*10^+00*** | 1.189*10^+00*** |
| Stocking Density 2 | 9.050*10^-01*** | 1.286*10^+00*** | 9.746*10^-01*** |
| Stocking Density 3 | 6.238*10^-01*** | 1.005*10^+00*** | 6.319*10^-01*** |
| Stocking Density 4 | 1.867*10^-01*** | 4.630*10^-01*** | 2.073*10^-01*** |
| Stocking Density 5 | - | - | - |
| Constant | 1.764*10^+02*** | 5.383*10^+02 | -1.269*10^+02*** |
| Observations | 29,796 | 22,271 | 104,997 |
| R-squared | 0.643 | 0.591 | 0.596 |

Note: All regressions employed county fixed effects. ***p<0.01//**p<0.05//*p<0.10

**Table S7. Estimated change in wood volume from 1960 to 2023 due to changes in $CO_2$ and climate, by forest group and age and based on observations of naturally regenerated stands aged 1 to 150, using regression results from Tables S4-S6.**

| | | | $CO_2$ | | | | Climate | | | | Both | | | | Sample |
|---|---|---|---|---|---|---|---|---|---|---|---|---|---|---|---|
| | | Age (years) | 25 | 50 | 75 | 100 | 25 | 50 | 75 | 100 | 25 | 50 | 75 | 100 | # |
| Oak/ Hickory | Naturally Regenerated | Δ (%) | 27.4 | 21.8 | 17.7 | 15.4 | 14.6 | 26.6 | 20.1 | 12.4 | 42.0 | 48.3 | 37.8 | 27.8 | 471,966 |
| | | Δ (m3/ha) | 6.9 | 12.1 | 15.7 | 19.9 | 3.7 | 14.8 | 17.9 | 16.1 | 10.6 | 26.9 | 33.6 | 24.8 | |
| | | Significance | *** | | | | *** | | | | | | | | |
| Oak/ Gum/ Cypress | Naturally Regenerated | Δ (%) | 27.1 | 21.5 | 17.4 | 15.2 | 6.4 | 15.5 | 18.7 | 14.8 | 33.5 | 37.0 | 36.1 | 30.0 | 88,241 |
| | | Δ (m3/ha) | 12.3 | 22.9 | 28.5 | 34.5 | 2.9 | 16.5 | 30.6 | 33.6 | 15.3 | 39.4 | 59.1 | 49.1 | |
| | | Significance | *** | | | | *** | | | | | | | | |
| Oak/ Pine | Naturally Regenerated | Δ (%) | 27.4 | 21.7 | 17.6 | 15.4 | 17.3 | 21.0 | 15.8 | 12.5 | 44.7 | 42.7 | 33.5 | 27.9 | 97,667 |
| | | Δ (m3/ha) | 11.9 | 20.5 | 25.7 | 31.7 | 7.5 | 19.8 | 23.1 | 25.8 | 19.4 | 40.2 | 48.9 | 40.7 | |
| | | Significance | *** | | | | *** | | | | | | | | |
| Elm/ Ash/ Cottonwood | Naturally Regenerated | Δ (%) | 28.9 | 22.9 | 18.6 | 16.2 | -7.7 | 7.0 | 2.5 | -3.0 | 21.1 | 29.9 | 21.1 | 13.2 | 101,220 |
| | | Δ (m3/ha) | 10.5 | 18.3 | 24.7 | 31.9 | -2.8 | 5.6 | 3.3 | -6.0 | 7.7 | 23.9 | 28.0 | 17.5 | |
| | | Significance | *** | | | | *** | | | | | | | | |
| Maple/ Beech/ Birch | Naturally Regenerated | Δ (%) | 26.1 | 20.7 | 16.8 | 14.7 | -10.2 | 3.7 | -4.5 | -12.1 | 15.9 | 24.4 | 12.4 | 2.6 | 162,130 |
| | | Δ (m3/ha) | 21.8 | 39.6 | 54.1 | 69.3 | -8.5 | 7.1 | -14.3 | -57.2 | 13.2 | 46.7 | 39.8 | 8.3 | |
| | | Significance | *** | | | | *** | | | | | | | | |
| Aspen/ Birch | Naturally Regenerated | Δ (%) | 33.0 | 26.2 | 21.3 | 18.6 | -0.5 | 11.2 | 5.9 | 1.3 | 32.5 | 37.5 | 27.1 | 19.9 | 110,632 |
| | | Δ (m3/ha) | 20.2 | 39.2 | 54.8 | 74.9 | -0.3 | 16.8 | 15.1 | 5.3 | 19.9 | 56.0 | 69.9 | 51.1 | |
| | | Significance | *** | | | | *** | | | | | | | | |
| Spruce/ Fir | Naturally Regenerated | Δ (%) | 28.9 | 23.0 | 18.6 | 16.3 | -37.4 | -30.4 | -25.9 | -24.1 | -8.4 | -7.4 | -7.3 | -7.9 | 64,867 |
| | | Δ (m3/ha) | 17.3 | 30.8 | 40.5 | 53.1 | -22.3 | -40.8 | -56.3 | -78.9 | -5.0 | -10.0 | -15.8 | -17.1 | |
| | | Significance | *** | | | | *** | | | | | | | | |
| White/ Red/ Jack Pine | Naturally Regenerated | Δ (%) | 28.3 | 22.5 | 18.2 | 15.9 | -13.8 | 4.7 | -7.5 | -13.3 | 14.4 | 27.1 | 10.7 | 2.5 | 29,796 |
| | | Δ (m3/ha) | 5.9 | 10.0 | 13.8 | 18.3 | -2.9 | 2.1 | -5.7 | -15.4 | 3.0 | 12.1 | 8.1 | 1.9 | |
| | | Significance | *** | | | | *** | | | | | | | | |
| Loblolly/ Shortleaf Pine | Naturally Regenerated | Δ (%) | 25.3 | 20.1 | 16.3 | 14.2 | 17.0 | 11.9 | 9.3 | 8.4 | 42.3 | 32.0 | 25.6 | 22.7 | 104,997 |
| | | Δ (m3/ha) | 21.3 | 36.4 | 44.0 | 51.5 | 14.3 | 21.6 | 25.1 | 30.6 | 35.6 | 58.0 | 69.1 | 61.2 | |
| | | Significance | *** | | | | *** | | | | | | | | |
| Slash/ Longleaf Pine | Naturally Regenerated | Δ (%) | 28.7 | 22.7 | 18.5 | 16.1 | 28.7 | 30.6 | 18.7 | 11.3 | 57.3 | 53.4 | 37.2 | 27.4 | 22,271 |
| | | Δ (m3/ha) | 2.5 | 4.4 | 5.9 | 7.5 | 2.5 | 5.9 | 5.9 | 5.3 | 5.1 | 10.3 | 11.8 | 8.7 | |
| | | Significance | *** | | | | *** | | | | | | | | |

*Note:* The impact of the changes in $CO_2$ and climate are a calculated difference between the expected volume in 1960 and 2023, given the age-specific lifetime exposures in each year. ***p<0.01.

**Table S8. Regression model raw output by pine forest group, based on observations of all stands aged 1 to 50.**

| All Stands | Natural Log of Volume per Hectare | | |
|---|---|---|---|
| Variables | White/Red/Jack | Slash/Longleaf | Loblolly/Shortleaf |
| Natural Log of Lifetime $CO_2$ | 1.682*10^+00*** | 1.888*10^+00*** | 1.862*10^+00*** |
| 1/Age | -9.721*10^+00** | 1.912*10^+01*** | 3.295*10^+00*** |
| Management | 3.789*10^-01*** | 4.667*10^-01*** | 3.662*10^-01*** |
| Management-Age Interaction | -5.018*10^+00*** | -5.459*10^+00*** | -3.562*10^+00*** |
| Mean Spring Temperature | 7.050*10^-01*** | 5.322*10^+00 | 7.164*10^-01 |
| Mean Spring Temperature Squared | -2.827*10^-02 | -2.852*10^-01 | -2.870*10^-02 |
| Mean Spring Temperature Cubed | -6.184*10^-04 | 4.902*10^-03 | 2.037*10^-04 |
| Mean Summer Temperature | -2.118*10^+01*** | -6.235*10^+01 | 5.392*10^+00 |
| Mean Summer Temperature Squared | 9.880*10^-01*** | 2.317*10^+00 | -1.827*10^-01 |
| Mean Summer Temperature Cubed | -1.503*10^-02*** | -2.879*10^-02 | 2.063*10^-03 |
| Mean Fall Temperature | -7.394*10^-02 | 3.040*10^+00 | -5.202*10^+00*** |
| Mean Fall Temperature Squared | -5.381*10^-02 | -1.517*10^-01 | 2.696*10^-01*** |
| Mean Fall Temperature Cubed | 2.622*10^-03 | 2.808*10^-03 | -4.512*10^-03*** |
| Mean Winter Temperature | -1.942*10^-01*** | -5.427*10^-01 | 1.104*10^-01** |
| Mean Winter Temperature Squared | 7.727*10^-03 | 5.478*10^-02 | 1.465*10^-03 |
| Mean Winter Temperature Cubed | 1.126*10^-03*** | -1.407*10^-03 | 3.433*10^-04 |
| Mean Spring Precipitation | -6.142*10^-02*** | -2.866*10^-03 | -1.621*10^-02*** |
| Mean Spring Precipitation Squared | 3.917*10^-04** | -1.27*10^-05 | 1.299*10^-04*** |
| Mean Spring Precipitation Cubed | -5.94*10^-07 | 5.21*10^-08 | -3.65*10^-07*** |
| Mean Summer Precipitation | 1.268*10^-01** | -8.878*10^-04 | 1.123*10^-02*** |
| Mean Summer Precipitation Squared | -1.227*10^-03** | 5.45*10^-05 | -1.44*10^-05 |
| Mean Summer Precipitation Cubed | 4.03*10^-06** | -1.43*10^-07 | -3.42*10^-08 |
| Mean Fall Precipitation | -2.239*10^-01*** | 1.207*10^-02 | 2.612*10^-02*** |
| Mean Fall Precipitation Squared | 2.504*10^-03*** | -6.62*10^-05 | -1.676*10^-04*** |
| Mean Fall Precipitation Cubed | -8.79*10^-06*** | 6.55*10^-08 | 2.73*10^-07*** |
| Mean Winter Precipitation | 5.288*10^-02*** | 1.796*10^-02 | 3.211*10^-02*** |
| Mean Winter Precipitation Squared | -6.389*10^-04*** | -7.24*10^-05 | -1.771*10^-04*** |
| Mean Winter Precipitation Cubed | 2.25*10^-06*** | -1.34*10^-07 | 3.61*10^-07*** |
| Spring Temperature-1/Age Interaction | -7.143*10^-01*** | -2.305*10^-02 | -3.284*10^-01*** |
| Summer Temperature-1/Age Interaction | 7.969*10^-01*** | -5.497*10^-01*** | 3.246*10^-01*** |
| Fall Temperature-1/Age Interaction | 6.011*10^-01*** | -2.125*10^-01** | -1.422*10^-01*** |
| Winter Temperature-1/Age Interaction | -2.789*10^-01*** | 5.224*10^-01*** | 1.788*10^-01*** |
| Spring Precipitation-1/Age Interaction | 4.107*10^-02*** | 1.673*10^-02*** | 7.926*10^-03*** |
| Summer Precipitation-1/Age Interaction | 1.421*10^-02 | 5.585*10^-03*** | 1.169*10^-02*** |
| Fall Precipitation-1/Age Interaction | -4.843*10^-03 | 6.080*10^-03*** | 4.056*10^-03*** |
| Winter Precipitation-1/Age Interaction | -2.982*10^-02** | 1.770*10^-02*** | 1.678*10^-03** |
| Site Class 1 | 6.500*10^-01*** | 5.639*10^-01*** | 2.601*10^-01*** |
| Site Class 2 | 8.008*10^-01*** | 5.418*10^-01*** | 8.579*10^-01*** |
| Site Class 3 | 8.117*10^-01*** | 8.063*10^-01*** | 6.780*10^-01*** |
| Site Class 4 | 6.140*10^-01*** | 6.533*10^-01*** | 5.268*10^-01*** |
| Site Class 5 | 4.025*10^-01*** | 4.269*10^-01*** | 3.323*10^-01*** |
| Site Class 6 | - | - | - |
| Time Dummy (2001 to 2023) | -1.107*10^-01** | -9.624*10^-02*** | -1.005*10^-01*** |
| Time Dummy (1981 to 2000) | -2.061*10^-01*** | -9.414*10^-02*** | -9.932*10^-02*** |
| Time Dummy (≤1980) | - | - | - |
| Elevation | -7.54*10^-05** | -1.909*10^-04* | -1.228*10^-04*** |
| Slope | 4.520*10^-03*** | 3.540*10^-03* | -1.455*10^-03*** |
| Disturbances | 3.658*10^-02 | 7.561*10^-02*** | 2.906*10^-02*** |
| Interventions | -5.248*10^-02** | 3.655*10^-04 | 2.092*10^-02*** |
| Xeric Physiography | -1.970*10^-01*** | -2.099*10^-01*** | -2.116*10^-01*** |
| Hydric Physiography | -7.762*10^-01*** | -1.603*10^-01*** | -2.129*10^-01*** |
| Mesic Physiography | - | - | - |
| North-facing Aspect | -4.572*10^-03 | 9.765*10^-02*** | 1.691*10^-02*** |
| Null Aspect | 1.983*10^-02 | 7.421*10^-02*** | 2.919*10^-02*** |
| South-facing Aspect | - | - | - |
| Private Land Ownership | 7.018*10^-02*** | 5.679*10^-02*** | 4.600*10^-04 |
| Latitude | -2.763*10^-01*** | 3.102*10^-01*** | 6.672*10^-02*** |
| Longitude | -1.356*10^-01*** | 1.382*10^-02 | -3.137*10^-02** |
| Stocking Density 1 | 7.333*10^-01*** | 1.558*10^+00*** | 9.926*10^-01*** |
| Stocking Density 2 | 6.703*10^-01*** | 1.210*10^+00*** | 7.306*10^-01*** |
| Stocking Density 3 | 5.160*10^-01*** | 8.839*10^-01*** | 4.078*10^-01*** |
| Stocking Density 4 | 1.444*10^-01*** | 4.094*10^-01*** | 5.550*10^-02*** |
| Stocking Density 5 | - | - | - |
| Constant | 1.384*10^+02*** | 4.832*10^+02 | -4.857*10^+01* |
| Observations | 23,450 | 38,906 | 189,878 |
| R-squared | 0.586 | 0.540 | 0.536 |

Note: Both regressions employed county fixed effects. ***p<0.01//**p<0.05//*p<0.10

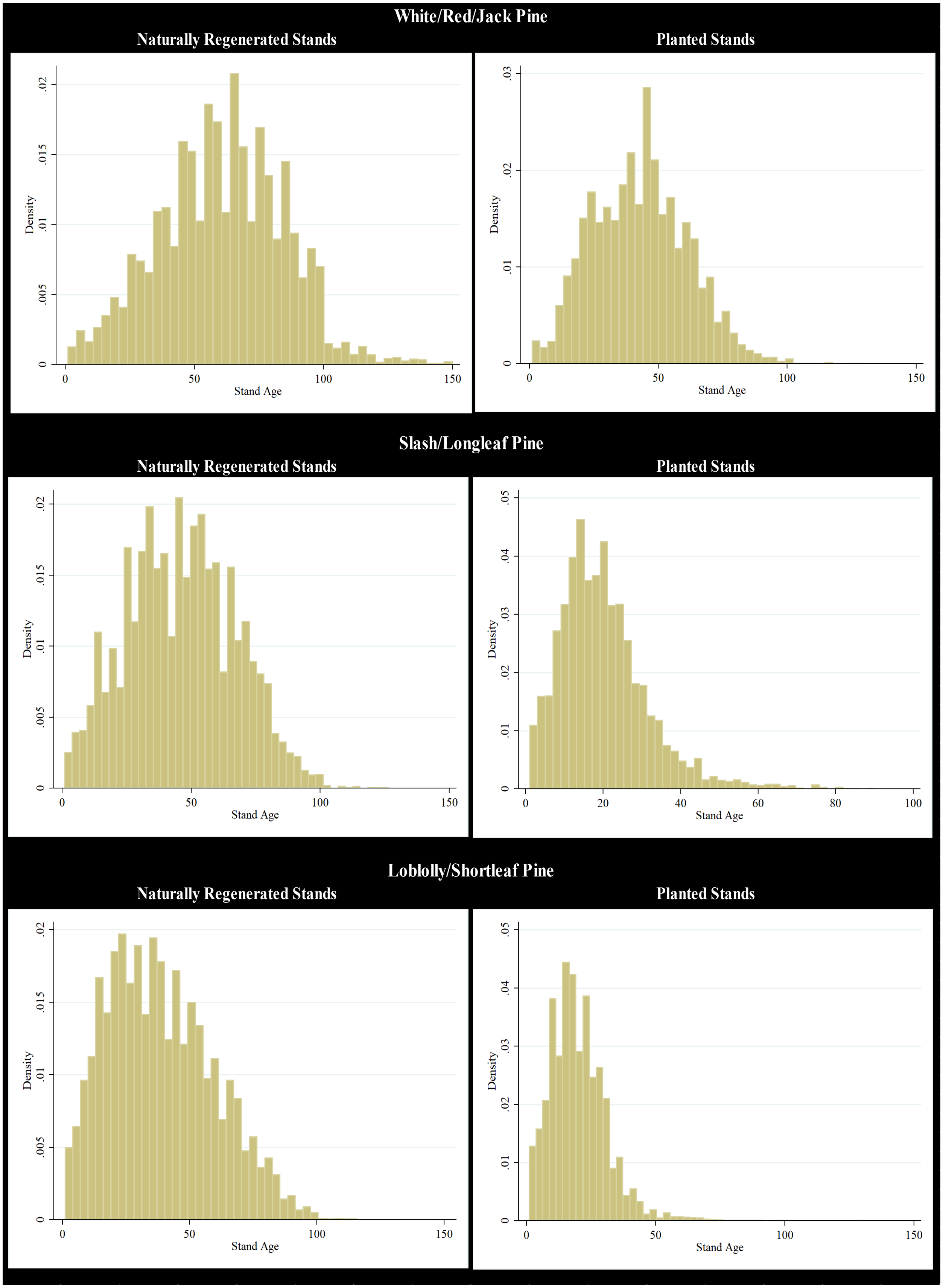


**Fig. S26. Histograms of naturally regenerated and planted plots by age and forest group.**

**Table S9. Estimated difference in wood volume between naturally regenerated and planted stands in 2023, by age and pine forest group, based on all observations aged 1 to 50.**

| | Age (years) | 10 | 20 | 25 | 30 | 40 | Obs. |
|---|---|---|---|---|---|---|---|
| Loblolly/ Shortleaf | Δ (%) | 1.0 | 18.8 | 22.4 | 24.8 | 27.7 | 189,878 |
| | 99% CI | (-3.4, 5.4) | (15.7, 21.9) | (19.5, 25.2) | (22.0, 27.5) | (25.2, 30.2) | |
| | Δ ($m^3$/ha) | 0.6 | 23.0 | 31.5 | 47.1 | 98.6 | |
| | 99% CI | (-2.0, 3.1) | (19.2, 26.8) | (27.5, 35.5) | (41.9, 52.4) | (89.6, 107.4) | |
| Slash/ Longleaf | Δ (%) | -7.9 | 19.4 | 24.8 | 28.5 | 33.0 | 38,906 |
| | 99% CI | (-18.2, 2.3) | (12.0, 26.7) | (18.1, 31.6) | (22.1, 34.8) | (27.2, 38.9) | |
| | Δ ($m^3$/ha) | -2.4 | 11.6 | 19.4 | 31.5 | 57.6 | |
| | 99% CI | (-5.6, 0.7) | (7.2, 16.1) | (14.1, 24.7) | (24.4, 38.5) | (47.5, 67.9) | |
| White/Red/Jack | Δ (%) | -12.3 | 12.8 | 17.8 | 21.2 | 25.3 | 23,450 |
| | 99% CI | (-25.0, 0.4) | (4.2, 21.4) | (10.0, 25.7) | (13.9, 28.5) | (18.7, 32.0) | |
| | Δ ($m^3$/ha) | -8.9 | 16.3 | 31.7 | 34.0 | 77.3 | |
| | 99% CI | (-18.0, 0.3) | (5.4, 27.3) | (17.8, 45.7) | (22.3, 45.8) | (57.0, 97.5) | |

**Table S10. Volume and area in 1974 of stands aged 1 to 100 years in 12 southern states, by forest group and regeneration method.**

| 1974 | Oak/ Pine | Oak/ Hickory | Oak/Gum/ Cypress | Elm/Ash/ Cottonwood | Maple/Beech/ /Birch | White/Red/ Jack | | Slash/ Longleaf | | Loblolly/ Shortleaf | |
|---|---|---|---|---|---|---|---|---|---|---|---|
| | Naturally Regenerated | Naturally Regenerated | Naturally Regenerated | Naturally Regenerated | Naturally Regenerated | Naturally Regenerated | Planted | Naturally Regenerated | Planted | Naturally Regenerated | Planted |
| **Age Class** | **Volume (cubic meters)** | | | | | | | | | | |
| 0-10 years | 13,155,899 | 21,711,681 | 4,224,446 | 1,938,688 | 0 | 215,238 | 0 | 2,237,492 | 9,822,651 | 25,720,432 | 12,039,481 |
| 11-20 years | 63,933,047 | 62,747,664 | 13,635,027 | 3,770,621 | 118,954 | 141,815 | 774,252 | 16,913,813 | 42,586,110 | 152,202,939 | 50,392,529 |
| 21-30 years | 151,795,627 | 146,836,661 | 46,922,621 | 15,645,331 | 471,205 | 1,037,725 | 716,543 | 38,879,181 | 17,118,507 | 350,019,857 | 37,630,286 |
| 31-40 years | 189,753,172 | 284,330,266 | 121,801,991 | 29,203,844 | 2,615,819 | 4,246,055 | 684,591 | 57,276,543 | 9,174,874 | 367,503,333 | 17,820,808 |
| 41-50 years | 134,175,247 | 297,211,840 | 149,680,650 | 37,024,000 | 2,432,547 | 3,297,613 | 0 | 45,500,240 | 1,113,442 | 241,086,298 | 3,640,642 |
| 51-60 years | 79,059,027 | 239,744,472 | 138,845,262 | 31,988,413 | 4,983,692 | 3,007,647 | 0 | 17,843,193 | 465,572 | 129,445,161 | 823,585 |
| 61-70 years | 35,553,760 | 150,421,721 | 86,804,866 | 15,207,416 | 4,299,841 | 210,655 | 0 | 6,972,923 | 0 | 59,518,745 | 36,453 |
| 71-80 years | 15,145,527 | 69,510,180 | 45,341,423 | 10,605,679 | 1,401,649 | 930,028 | 0 | 1,423,969 | 0 | 24,593,818 | 0 |
| 81-90 years | 11,550,162 | 60,375,422 | 29,069,290 | 2,230,154 | 2,211,300 | 340,162 | 0 | 532,062 | 0 | 10,255,946 | 0 |
| 91-100 years | 107,722,924 | 167,029,798 | 269,493,066 | 26,332,287 | 4,008,991 | 1,375,716 | 0 | 0 | 0 | 0 | 0 |
| **Total** | **801,844,392** | **1,499,919,704** | **905,818,641** | **173,946,433** | **22,543,999** | **14,802,656** | **2,175,385** | **187,579,418** | **80,281,155** | **1,360,346,529** | **122,383,784** |
| **Age Class** | **Area (hectares)** | | | | | | | | | | |
| 0-10 years | 869,672 | 1,941,818 | 322,777 | 135,516 | 0 | 11,131 | 0 | 156,437 | 1,368,875 | 1,512,415 | 1,169,821 |
| 11-20 years | 1,857,850 | 2,529,079 | 556,656 | 165,874 | 7,732 | 3,571 | 8,640 | 500,569 | 955,236 | 3,214,732 | 825,799 |
| 21-30 years | 2,533,480 | 3,420,650 | 943,286 | 294,149 | 9,720 | 11,396 | 4,896 | 598,175 | 188,578 | 3,870,288 | 371,958 |
| 31-40 years | 2,121,862 | 4,370,527 | 1,633,083 | 425,659 | 34,418 | 32,345 | 3,063 | 687,089 | 77,421 | 3,093,427 | 133,759 |
| 41-50 years | 1,320,682 | 3,527,141 | 1,553,844 | 432,974 | 25,058 | 19,890 | 0 | 448,089 | 13,250 | 1,789,093 | 32,893 |
| 51-60 years | 772,522 | 2,449,568 | 1,155,060 | 280,913 | 42,677 | 20,873 | 0 | 179,265 | 5,664 | 885,852 | 12,778 |
| 61-70 years | 301,038 | 1,367,734 | 582,220 | 113,786 | 28,386 | 1,594 | 0 | 69,785 | 1,377 | 420,895 | 1,683 |
| 71-80 years | 130,270 | 595,185 | 257,922 | 68,224 | 10,725 | 4,066 | 0 | 15,177 | 0 | 154,711 | 0 |
| 81-90 years | 91,259 | 488,315 | 153,067 | 15,945 | 13,565 | 3,943 | 0 | 4,982 | 0 | 69,787 | 0 |
| 91-100 years | 1,546,750 | 2,176,159 | 2,447,733 | 243,892 | 26,314 | 8,524 | 0 | 0 | 0 | 0 | 0 |
| **Total** | **11,545,386** | **22,866,175** | **9,605,648** | **2,176,932** | **198,595** | **117,335** | **16,599** | **2,659,569** | **2,610,400** | **15,011,200** | **2,548,691** |

Data approximate 1974 stocking levels, using USFS-FIA evaluations closest to 1974 (range: 1968 to 1980), in 12 states: AL, AR, FL, GA, LA, MS, NC, OK, SC, TN, TX, & VA. Volume is the net merchantable bole volume of growing-stock trees (at least 12.5 cm diameter at breast height), in cubic meters, on timberland. Area is the area of timberland in hectares.

**Table S11. Volume and area in 2005 of stands aged 1 to 100 years in 12 southern states, by forest group and regeneration method.**

| | 2005 | | | | | | | | | | |
|---|---|---|---|---|---|---|---|---|---|---|---|
| | **Oak/ Pine** | **Oak/ Hickory** | **Oak/Gum/ Cypress** | **Elm/Ash/ Cottonwood** | **Maple/Beech/ /Birch** | **White/Red/ Jack** | | **Slash/ Longleaf** | | **Loblolly/ Shortleaf** | |
| | Naturally Regenerated | Naturally Regenerated | Naturally Regenerated | Naturally Regenerated | Naturally Regenerated | Naturally Regenerated | Planted | Naturally Regenerated | Planted | Naturally Regenerated | Planted |
| **Age Class** | **Volume (cubic meters)** | | | | | | | | | | |
| 0-10 years | 14,241,758 | 49,639,277 | 10,926,052 | 3,688,966 | 369,008 | 5,293 | 0 | 2,880,376 | 5,107,581 | 22,471,354 | 32,910,307 |
| 11-20 years | 39,967,066 | 75,497,564 | 21,202,118 | 9,775,720 | 113,637 | 730,333 | 468,282 | 6,793,020 | 71,201,196 | 95,058,386 | 357,890,333 |
| 21-30 years | 56,131,892 | 114,386,689 | 45,660,643 | 15,688,355 | 365,760 | 1,831,698 | 2,210,129 | 17,415,975 | 65,078,868 | 171,951,756 | 375,545,333 |
| 31-40 years | 88,917,523 | 210,708,357 | 89,220,310 | 35,566,958 | 1,661,758 | 5,244,816 | 908,850 | 29,310,500 | 24,554,437 | 210,090,519 | 116,003,291 |
| 41-50 years | 138,073,672 | 389,916,018 | 168,902,615 | 50,567,527 | 4,130,452 | 5,080,839 | 526,126 | 48,436,369 | 14,743,724 | 295,675,829 | 34,058,721 |
| 51-60 years | 147,999,063 | 536,705,139 | 229,654,274 | 61,571,920 | 6,940,261 | 5,097,038 | 0 | 49,940,797 | 4,282,803 | 236,549,010 | 15,449,907 |
| 61-70 years | 109,878,539 | 573,490,528 | 194,051,066 | 42,716,056 | 5,993,196 | 5,364,035 | 188,840 | 40,592,170 | 4,534,861 | 179,613,544 | 4,946,902 |
| 71-80 years | 52,943,670 | 418,497,093 | 136,343,876 | 28,017,610 | 8,728,283 | 6,183,979 | 0 | 19,670,558 | 1,603,386 | 79,434,487 | 2,129,369 |
| 81-90 years | 31,604,158 | 216,898,228 | 86,406,452 | 10,688,493 | 2,949,561 | 1,408,255 | 0 | 6,907,902 | 643,303 | 28,288,082 | 1,128,592 |
| 91-100 years | 12,895,685 | 105,510,731 | 44,025,893 | 5,813,409 | 1,396,836 | 580,658 | 0 | 1,852,807 | 0 | 9,296,209 | 0 |
| **Total** | **692,653,027** | **2,691,249,624** | **1,026,393,300** | **264,095,014** | **32,648,752** | **31,526,943** | **4,302,227** | **223,800,474** | **191,750,158** | **1,328,429,176** | **940,062,756** |
| **Age Class** | **Area (hectares)** | | | | | | | | | | |
| 0-10 years | 843,131 | 3,605,860 | 757,540 | 390,106 | 10,426 | 611 | 0 | 116,538 | 1,042,820 | 1,044,290 | 3,704,902 |
| 11-20 years | 1,014,062 | 2,443,285 | 649,311 | 377,993 | 12,302 | 9,196 | 7,804 | 193,121 | 1,074,246 | 1,613,140 | 4,651,829 |
| 21-30 years | 810,923 | 1,926,061 | 709,805 | 297,948 | 6,752 | 11,941 | 11,914 | 238,140 | 608,903 | 1,496,891 | 2,921,064 |
| 31-40 years | 945,859 | 2,539,679 | 906,648 | 443,003 | 18,014 | 21,375 | 4,921 | 317,756 | 184,657 | 1,373,160 | 658,509 |
| 41-50 years | 1,105,967 | 3,566,042 | 1,308,359 | 497,429 | 30,001 | 21,123 | 2,616 | 391,933 | 102,151 | 1,611,743 | 181,228 |
| 51-60 years | 1,039,789 | 4,100,520 | 1,487,051 | 486,477 | 50,718 | 18,486 | 0 | 342,794 | 29,403 | 1,173,569 | 71,720 |
| 61-70 years | 707,565 | 3,837,637 | 1,110,650 | 342,467 | 47,116 | 19,605 | 1,044 | 285,195 | 24,275 | 834,289 | 27,666 |
| 71-80 years | 322,816 | 2,582,327 | 708,042 | 179,738 | 46,750 | 20,380 | 0 | 145,604 | 13,195 | 349,495 | 10,029 |
| 81-90 years | 182,112 | 1,347,048 | 415,789 | 76,649 | 14,689 | 4,269 | 0 | 51,420 | 4,014 | 114,221 | 4,800 |
| 91-100 years | 69,415 | 593,395 | 199,701 | 34,954 | 9,146 | 2,785 | 0 | 17,634 | 0 | 39,912 | 0 |
| **Total** | **7,041,639** | **26,541,854** | **8,252,896** | **3,126,765** | **245,913** | **129,770** | **28,298** | **2,100,135** | **3,083,664** | **9,650,711** | **12,231,745** |

Data approximate 2005 stocking levels, using USFS-FIA evaluations closest to 2005 (range: 2004 to 2010), in 12 states: AL, AR, FL, GA, LA, MS, NC, OK, SC, TN, TX, & VA. Volume is the net merchantable bole volume of growing-stock trees (at least 12.5 cm diameter at breast height), in cubic meters, on timberland. Area is the area of timberland in hectares.

**Table S12. Volume and area in 2022 of stands aged 1 to 100 years in 12 southern states, by forest group and regeneration method.**

| 2022 | Oak/ Pine | Oak/ Hickory | Oak/Gum/ Cypress | Elm/Ash/ Cottonwood | Maple/Beech/ /Birch | White/Red/ Jack | | Slash/ Longleaf | | Loblolly/ Shortleaf | |
|---|---|---|---|---|---|---|---|---|---|---|---|
| | Naturally Regenerated | Naturally Regenerated | Naturally Regenerated | Naturally Regenerated | Naturally Regenerated | Naturally Regenerated | Planted | Naturally Regenerated | Planted | Naturally Regenerated | Planted |
| **Age Class** | **Volume (cubic meters)** | | | | | | | | | | |
| 0-10 years | 9,288,809 | 23,024,001 | 4,112,310 | 1,818,060 | 257,030 | 13,180 | 0 | 1,502,875 | 5,536,433 | 20,565,927 | 59,074,870 |
| 11-20 years | 34,503,797 | 52,312,038 | 18,854,514 | 12,757,973 | 206,830 | 280,719 | 0 | 5,014,637 | 56,441,578 | 127,068,641 | 468,428,329 |
| 21-30 years | 82,166,807 | 127,946,507 | 50,488,171 | 20,128,729 | 124,877 | 2,083,196 | 2,370,832 | 25,891,588 | 77,061,879 | 323,090,996 | 695,619,875 |
| 31-40 years | 100,053,022 | 170,037,705 | 79,667,033 | 24,910,731 | 512,897 | 4,937,177 | 1,582,870 | 28,436,003 | 47,234,184 | 377,454,733 | 436,954,625 |
| 41-50 years | 101,987,376 | 243,769,655 | 106,458,615 | 31,588,158 | 992,625 | 5,702,555 | 1,484,053 | 36,078,356 | 16,166,006 | 273,223,868 | 126,084,221 |
| 51-60 years | 139,621,503 | 357,540,238 | 156,325,181 | 51,500,252 | 4,133,291 | 6,267,261 | 325,599 | 43,215,682 | 7,647,530 | 246,129,186 | 19,590,375 |
| 61-70 years | 160,513,859 | 562,256,716 | 218,898,289 | 60,547,945 | 8,117,458 | 7,030,982 | 207,832 | 55,765,395 | 5,101,147 | 231,622,382 | 15,815,681 |
| 71-80 years | 122,474,583 | 630,237,941 | 244,764,203 | 50,616,046 | 7,192,673 | 4,855,612 | 0 | 41,327,855 | 240,603 | 139,742,099 | 4,117,164 |
| 81-90 years | 79,043,016 | 525,703,124 | 164,934,236 | 23,034,423 | 9,603,794 | 7,394,527 | 0 | 27,318,350 | 342,439 | 92,581,422 | 2,715,092 |
| 91-100 years | 38,202,502 | 319,919,920 | 84,874,677 | 10,632,272 | 6,734,925 | 4,439,569 | 0 | 12,076,531 | 0 | 29,228,370 | 238,026 |
| **Total** | **867,855,273** | **3,012,747,846** | **1,129,377,228** | **287,534,588** | **37,876,400** | **43,004,779** | **5,971,186** | **276,627,273** | **215,771,800** | **1,860,707,624** | **1,828,638,257** |
| **Age Class** | **Area (hectares)** | | | | | | | | | | |
| 0-10 years | 545,656 | 2,062,846 | 389,266 | 255,861 | 6,409 | 1,934 | 0 | 93,562 | 687,130 | 838,522 | 3,712,936 |
| 11-20 years | 658,671 | 1,523,807 | 591,223 | 359,462 | 7,763 | 1,454 | 0 | 125,740 | 771,483 | 1,424,952 | 4,058,659 |
| 21-30 years | 920,512 | 1,774,404 | 735,650 | 349,590 | 6,993 | 16,384 | 9,600 | 275,101 | 641,619 | 1,989,989 | 3,886,460 |
| 31-40 years | 840,827 | 1,777,031 | 809,288 | 373,199 | 7,477 | 20,255 | 5,787 | 278,623 | 311,786 | 1,804,732 | 1,971,632 |
| 41-50 years | 773,805 | 2,095,311 | 946,882 | 399,219 | 11,148 | 26,552 | 5,479 | 315,502 | 118,064 | 1,250,857 | 462,370 |
| 51-60 years | 961,140 | 2,741,746 | 1,101,494 | 485,568 | 29,909 | 20,738 | 1,488 | 312,526 | 49,116 | 1,091,185 | 76,094 |
| 61-70 years | 927,762 | 3,595,284 | 1,298,810 | 443,272 | 50,347 | 24,823 | 571 | 329,359 | 24,227 | 908,682 | 51,219 |
| 71-80 years | 644,432 | 3,625,351 | 1,234,337 | 369,303 | 43,561 | 15,647 | 0 | 255,855 | 1,600 | 562,087 | 14,422 |
| 81-90 years | 396,861 | 2,702,562 | 758,137 | 139,162 | 44,963 | 21,182 | 0 | 156,150 | 1,883 | 346,335 | 5,338 |
| 91-100 years | 171,869 | 1,492,422 | 380,765 | 50,914 | 40,984 | 10,254 | 0 | 62,683 | 0 | 117,256 | 2,245 |
| **Total** | **6,841,535** | **23,390,765** | **8,245,852** | **3,225,549** | **249,555** | **159,223** | **22,924** | **2,205,102** | **2,606,908** | **10,334,597** | **14,241,375** |

Data approximate 2022 stocking levels, using USFS-FIA evaluations closest to 2022 (range: 2020 to 2022), in 12 states: AL, AR, FL, GA, LA, MS, NC, OK, SC, TN, TX, & VA. Volume is the net merchantable bole volume of growing-stock trees (at least 12.5 cm diameter at breast height), in cubic meters, on timberland. Area is the area of timberland in hectares.

**Table S13. Regression model raw output using regional aggregation of forest groups.**

| | Natural Log of Volume per Hectare | | |
|---|---|---|---|
| Variables | South | North | West |
| Natural Log of Lifetime $CO_2$ | 1.299*10^+00*** | 1.332*10^+00*** | 1.053*10^+00*** |
| 1/Age | 3.116*10^+00*** | -1.912*10^-01 | 3.662*10^+00*** |
| Management | 2.749*10^-01*** | 3.004*10^-01*** | 1.784*10^-01*** |
| Management-Age Interaction | -1.593*10^+00*** | -1.636*10^+00*** | -6.717*10^+00*** |
| Mean Spring Temperature | 4.160*10^-01*** | 3.629*10^-01*** | -1.602*10^-01*** |
| Mean Spring Temperature Squared | -2.155*10^-02*** | -1.185*10^-02*** | 2.472*10^-02*** |
| Mean Spring Temperature Cubed | 1.739*10^-04*** | -2.241*10^-04*** | -2.928*10^-04 |
| Mean Summer Temperature | -2.526*10^+00*** | -1.711*10^+00*** | 3.255*10^+00*** |
| Mean Summer Temperature Squared | 1.166*10^-01*** | 1.122*10^-01*** | -1.936*10^-01*** |
| Mean Summer Temperature Cubed | -1.766*10^-03*** | -2.047*10^-03*** | 3.683*10^-03*** |
| Mean Fall Temperature | 1.247*10^-01*** | 5.821*10^-01*** | 8.254*10^-01*** |
| Mean Fall Temperature Squared | -3.677*10^-02*** | -7.990*10^-02*** | -1.681*10^-02 |
| Mean Fall Temperature Cubed | 1.372*10^-03*** | 2.600*10^-03*** | -9.446*10^-04* |
| Mean Winter Temperature | -2.464*10^-01*** | -3.218*10^-01*** | -3.366*10^-01*** |
| Mean Winter Temperature Squared | 1.204*10^-02*** | 1.321*10^-02*** | 3.367*10^-02*** |
| Mean Winter Temperature Cubed | 3.437*10^-04*** | 5.916*10^-04*** | 2.459*10^-03*** |
| Mean Spring Precipitation | -9.187*10^-03*** | -1.079*10^-02*** | -3.089*10^-02*** |
| Mean Spring Precipitation Squared | 8.10*10^-05*** | 7.54*10^-05*** | 1.223*10^-04*** |
| Mean Spring Precipitation Cubed | -2.57*10^-07*** | -2.10*10^-07*** | -1.56*10^-07*** |
| Mean Summer Precipitation | 5.656*10^-03*** | 1.821*10^-02*** | 4.217*10^-02*** |
| Mean Summer Precipitation Squared | -1.76*10^-05** | -8.88*10^-05*** | -2.689*10^-04*** |
| Mean Summer Precipitation Cubed | -1.67*10^-08 | 1.13*10^-07*** | 5.64*10^-07 |
| Mean Fall Precipitation | 2.636*10^-02*** | -1.002*10^-02*** | -6.100*10^-03*** |
| Mean Fall Precipitation Squared | -1.319*10^-04*** | 1.269*10^-04*** | -2.11*10^-05* |
| Mean Fall Precipitation Cubed | 2.31*10^-07*** | -2.91*10^-07*** | 2.36*10^-08 |
| Mean Winter Precipitation | -5.887*10^-03*** | -2.027*10^-02*** | 2.499*10^-02*** |
| Mean Winter Precipitation Squared | 9.78*10^-05*** | 1.941*10^-04*** | -5.10*10^-05*** |
| Mean Winter Precipitation Cubed | -2.18*10^-07*** | -3.82*10^-07*** | 4.02*10^-08*** |
| Spring Temperature -Age Interaction | -2.959*10^-01*** | -1.956*10^-01*** | -7.490*10^-01*** |
| Summer Temperature -Age Interaction | 2.571*10^-01*** | 7.589*10^-01*** | 8.731*10^-01*** |
| Fall Temperature -Age Interaction | -2.502*10^-01*** | -8.506*10^-01*** | -3.215*10^-01*** |
| Winter Temperature -Age Interaction | 9.098*10^-02*** | 2.170*10^-01*** | 1.451*10^-01** |
| Spring Precipitation -Age Interaction | 1.222*10^-02*** | 1.119*10^-02*** | 5.701*10^-03*** |
| Summer Precipitation -Age Interaction | 1.780*10^-02*** | 1.494*10^-02*** | -8.040*10^-02*** |
| Fall Precipitation -Age Interaction | -9.975*10^-03*** | -1.658*10^-02*** | 2.794*10^-02*** |
| Winter Precipitation -Age Interaction | 1.455*10^-03*** | -4.213*10^-03*** | -1.617*10^-02*** |
| Site Class 1 | 2.882*10^-01*** | 3.563*10^-01*** | 6.654*10^-01*** |
| Site Class 2 | 6.031*10^-01*** | 6.607*10^-01*** | 9.158*10^-01*** |
| Site Class 3 | 5.001*10^-01*** | 5.496*10^-01*** | 7.517*10^-01*** |
| Site Class 4 | 3.608*10^-01*** | 4.177*10^-01*** | 5.787*10^-01*** |
| Site Class 5 | 1.970*10^-01*** | 2.522*10^-01*** | 3.256*10^-01*** |
| Site Class 6 | - | - | - |
| Time Dummy (2001 to 2023) | -8.997*10^-02*** | -1.212*10^-01*** | |
| Time Dummy (1981 to 2000) | -1.056*10^-01*** | -9.445*10^-02*** | |
| Time Dummy (≤1980) | - | - | |
| Time Dummy (2017 to 2023) | | | -1.157*10^-02*** |
| Time Dummy (2011 to 2016) | | | 2.297*10^-02 |
| Time Dummy (≤2010) | | | - |
| Elevation | 2.10*10^-05*** | -2.80*10^-05*** | -1.98*10^-05*** |
| Slope | 7.51*10^-05* | 2.534*10^-04*** | -4.660*10^-04*** |
| Disturbances | -3.877*10^-03*** | -1.048*10^-02*** | 1.626*10^-01*** |
| Interventions | 2.598*10^-02*** | -7.240*10^-03*** | -1.340*10^-01*** |
| Xeric Physiography | -2.286*10^-01*** | -2.202*10^-01*** | -1.254*10^-01*** |
| Hydric Physiography | -1.493*10^-01*** | -4.101*10^-01*** | -5.089*10^-02* |
| Mesic Physiography | - | - | - |
| North-facing Aspect | 1.529*10^-02*** | 1.347*10^-02*** | -1.220*10^-02*** |
| Null Aspect | -1.024*10^-02*** | -6.039*10^-02*** | -4.789*10^-02*** |
| South-facing Aspect | - | - | - |
| Private Land Ownership | 9.843*10^-04 | 1.597*10^-02*** | -1.661*10^-01*** |
| Latitude | 7.451*10^-03*** | -1.946*10^-02*** | -1.049*10^-02* |
| Longitude | -1.690*10^-02*** | 3.400*10^-02*** | -5.212*10^-02*** |
| Stocking Density 1 | 1.023*10^+00*** | 7.067*10^-01*** | 2.115*10^+00*** |
| Stocking Density 2 | 8.698*10^-01*** | 8.124*10^-01*** | 1.923*10^+00*** |
| Stocking Density 3 | 5.721*10^-01*** | 5.857*10^-01*** | 1.618*10^+00*** |
| Stocking Density 4 | 2.040*10^-01*** | 2.456*10^-01*** | 1.082*10^+00*** |
| Stocking Density 5 | - | - | - |
| White/Red/Jack pine | 3.217*10^-01*** | 3.233*10^-01*** | |
| Spruce/Fir | | -1.881*10^-01*** | |
| Loblolly/Shortleaf pine | 3.120*10^-01*** | 3.409*10^-01*** | |
| Oak/Pine | 8.269*10^-02*** | 9.173*10^-02*** | |
| Oak/Gum/Cypress | 1.791*10^-01*** | 2.723*10^-01*** | |
| Elm/Ash/Cottonwood | -1.929*10^-02*** | 4.243*10^-02*** | |
| Maple/Beech/Birch | 8.908*10^-02*** | 6.066*10^-02*** | |
| Aspen/Birch | | 2.820*10^-02*** | |
| Slash/Longleaf pine | 1.557*10^-01*** | | |
| Oak/Hickory | - | - | |
| Ponderosa pine | | | -1.867*10^-01*** |
| Fir/Spruce | | | -2.416*10^-01*** |
| Lodgepole pine | | | -1.272*10^-01*** |
| Douglas-Fir | | | - |
| Constant | 6.541*10^+00*** | -1.044*10^+00 | -3.682*10^+01*** |
| Observations | 2,887,222 | 4,238,139 | 140,853 |
| R-squared | 0.498 | 0.479 | 0.637 |

Note: The Southern aggregation included all observations aged 1 to 150 years of: Elm/Ash/Cottonwood, Oak/Pine, Oak/Hickory, Maple/Beech/Birch, White/Red/Jack Pine, Spruce/Fir, Oak/Gum/Cypress, Loblolly/Shortleaf Pine, and Slash/Shortleaf Pine. The Northern aggregation included all observations aged 1 to 150 years of: Elm/Ash/Cottonwood, Oak/Pine, Oak/Hickory, Maple/Beech/Birch, White/Red/Jack Pine, Aspen/Birch,, Spruce/Fir, Oak/Gum/Cypress, and Loblolly/Shortleaf Pine. The Western aggregation included all observations aged 1 to 200 years of: Douglas-Fir, Fir/Spruce, Ponderosa Pine, and Lodgepole Pine. All regressions employed county fixed effects. ***p<0.01//**p<0.05//*p<0.10

**Table S14. Regression model raw output by forest group and for all stands aged 1 to 150 years.**

| All Stands | Natural Log of Volume per Hectare | | |
|---|---|---|---|
| Variables | White/Red/Jack | Slash/Longleaf | Loblolly/Shortleaf |
| Natural Log of Lifetime $CO_2$ | 1.294*10^+00*** | 1.582*10^+00*** | 1.530*10^+00*** |
| 1/Age | -8.797*10^+00*** | 9.502*10^+00*** | 2.307*10^+00*** |
| Management | 2.521*10^-01*** | 4.365*10^-01*** | 2.977*10^-01*** |
| Management-Age Interaction | -3.311*10^+00*** | -4.419*10^+00*** | -2.004*10^+00*** |
| Mean Spring Temperature | 6.125*10^-01*** | 3.038*10^+00 | 2.343*10^+00*** |
| Mean Spring Temperature Squared | -1.884*10^-02 | -1.757*10^-01 | -1.464*10^-01*** |
| Mean Spring Temperature Cubed | -9.389*10^-04 | 3.043*10^-03 | 2.670*10^-03*** |
| Mean Summer Temperature | -1.269*10^+01*** | 1.147*10^+01 | 8.791*10^+00*** |
| Mean Summer Temperature Squared | 5.583*10^-01*** | -4.337*10^-01 | -2.844*10^-01*** |
| Mean Summer Temperature Cubed | -7.756*10^-03*** | 5.419*10^-03 | 3.068*10^-03** |
| Mean Fall Temperature | 7.577*10^-01*** | 8.316*10^+00*** | -7.029*10^+00*** |
| Mean Fall Temperature Squared | -1.433*10^-01*** | -4.236*10^-01*** | 3.651*10^-01*** |
| Mean Fall Temperature Cubed | 5.166*10^-03*** | 7.552*10^-03*** | -6.092*10^-03*** |
| Mean Winter Temperature | -3.415*10^-01*** | -1.114*10^+00*** | -1.099*10^-01** |
| Mean Winter Temperature Squared | 6.424*10^-03 | 9.327*10^-02*** | 2.111*10^-02*** |
| Mean Winter Temperature Cubed | 1.023*10^-03*** | -2.447*10^-03*** | -4.524*10^-04* |
| Mean Spring Precipitation | -1.270*10^-01*** | -1.033*10^-02 | -1.710*10^-02*** |
| Mean Spring Precipitation Squared | 1.063*10^-03*** | 6.20*10^-05 | 1.383*10^-04*** |
| Mean Spring Precipitation Cubed | -2.66*10^-06*** | -2.03*10^-07 | -4.07*10^-07*** |
| Mean Summer Precipitation | 2.557*10^-01*** | -9.036*10^-03 | 1.410*10^-02*** |
| Mean Summer Precipitation Squared | -2.363*10^-03*** | 8.92*10^-05* | -3.38*10^-05* |
| Mean Summer Precipitation Cubed | 7.43*10^-06*** | -1.94*10^-07** | 1.42*10^-08 |
| Mean Fall Precipitation | -1.606*10^-01*** | 2.586*10^-02*** | 3.950*10^-02*** |
| Mean Fall Precipitation Squared | 1.766*10^-03*** | -1.326*10^-04 | -2.360*10^-04*** |
| Mean Fall Precipitation Cubed | -5.86*10^-06*** | 1.81*10^-07 | 4.32*10^-07*** |
| Mean Winter Precipitation | 4.978*10^-02*** | 1.819*10^-02** | 3.140*10^-02*** |
| Mean Winter Precipitation Squared | -5.272*10^-04*** | -6.76*10^-05 | -1.468*10^-04*** |
| Mean Winter Precipitation Cubed | 1.75*10^-06*** | -8.14*10^-08 | 3.00*10^-07*** |
| Spring Temperature-1/Age Interaction | -3.724*10^-01*** | 1.696*10^-01* | -6.442*10^-02** |
| Summer Temperature-1/Age Interaction | 4.931*10^-01*** | -2.885*10^-01** | 1.876*10^-01*** |
| Fall Temperature-1/Age Interaction | 8.585*10^-01*** | -4.510*10^-01*** | -2.365*10^-01*** |
| Winter Temperature-1/Age Interaction | -3.707*10^-01*** | 6.100*10^-01*** | 1.780*10^-01*** |
| Spring Precipitation-1/Age Interaction | 8.085*10^-02*** | 1.944*10^-02*** | 1.017*10^-02*** |
| Summer Precipitation-1/Age Interaction | -2.313*10^-02*** | 6.193*10^-03*** | 9.326*10^-03*** |
| Fall Precipitation-1/Age Interaction | -3.806*10^-02*** | 4.349*10^-03** | -2.486*10^-03*** |
| Winter Precipitation-1/Age Interaction | -3.567*10^-02*** | 1.716*10^-02*** | -2.372*10^-03*** |
| Site Class 1 | 5.711*10^-01*** | 5.283*10^-01*** | 3.115*10^-01*** |
| Site Class 2 | 6.360*10^-01*** | 4.706*10^-01*** | 8.745*10^-01*** |
| Site Class 3 | 6.519*10^-01*** | 7.895*10^-01*** | 6.921*10^-01*** |
| Site Class 4 | 4.633*10^-01*** | 6.438*10^-01*** | 5.387*10^-01*** |
| Site Class 5 | 2.900*10^-01*** | 4.056*10^-01*** | 3.359*10^-01*** |
| Site Class 6 | - | - | - |
| Time Dummy (2001 to 2023) | 1.046*10^-01*** | -1.764*10^-01*** | -1.647*10^-01*** |
| Time Dummy (1981 to 2000) | -1.144*10^-01*** | -1.335*10^-01*** | -1.562*10^-01*** |
| Time Dummy (≤1980) | - | - | - |
| Elevation | -6.79*10^-06 | -2.424*10^-04*** | -8.59*10^-05*** |
| Slope | 2.725*10^-03*** | 2.221*10^-03 | -1.835*10^-03*** |
| Disturbances | -2.598*10^-02** | 4.416*10^-02*** | 3.370*10^-02*** |
| Interventions | -6.226*10^-02*** | -2.286*10^-02** | 3.929*10^-03 |
| Xeric Physiography | -1.717*10^-01*** | -1.734*10^-01*** | -1.959*10^-01*** |
| Hydric Physiography | -4.046*10^-01*** | -1.011*10^-01*** | -2.534*10^-01*** |
| Mesic Physiography | - | - | - |
| North-facing Aspect | 7.529*10^-03 | 8.196*10^-02*** | 1.410*10^-02*** |
| Null Aspect | 4.926*10^-03 | 6.092*10^-03 | 2.473*10^-02*** |
| South-facing Aspect | - | - | - |
| Private Land Ownership | 6.363*10^-02*** | 9.591*10^-02*** | 5.741*10^-02*** |
| Latitude | -2.526*10^-01*** | 3.041*10^-01*** | 6.213*10^-02*** |
| Longitude | -7.462*10^-02*** | 7.154*10^-03 | -3.315*10^-02*** |
| Stocking Density 1 | 1.164*10^+00*** | 1.599*10^+00*** | 1.046*10^+00*** |
| Stocking Density 2 | 9.223*10^-01*** | 1.224*10^+00*** | 7.750*10^-01*** |
| Stocking Density 3 | 6.474*10^-01*** | 9.049*10^-01*** | 4.553*10^-01*** |
| Stocking Density 4 | 2.144*10^-01*** | 4.199*10^-01*** | 9.281*10^-02*** |
| Stocking Density 5 | - | - | - |
| Constant | 8.400*10^+01*** | -1.909*10^+02 | -7.760*10^+01*** |
| Observations | 51,526 | 52,262 | 238,216 |
| R-squared | 0.621 | 0.545 | 0.532 |

Note: All regressions employed county fixed effects. ***p<0.01//**p<0.05//*p<0.10

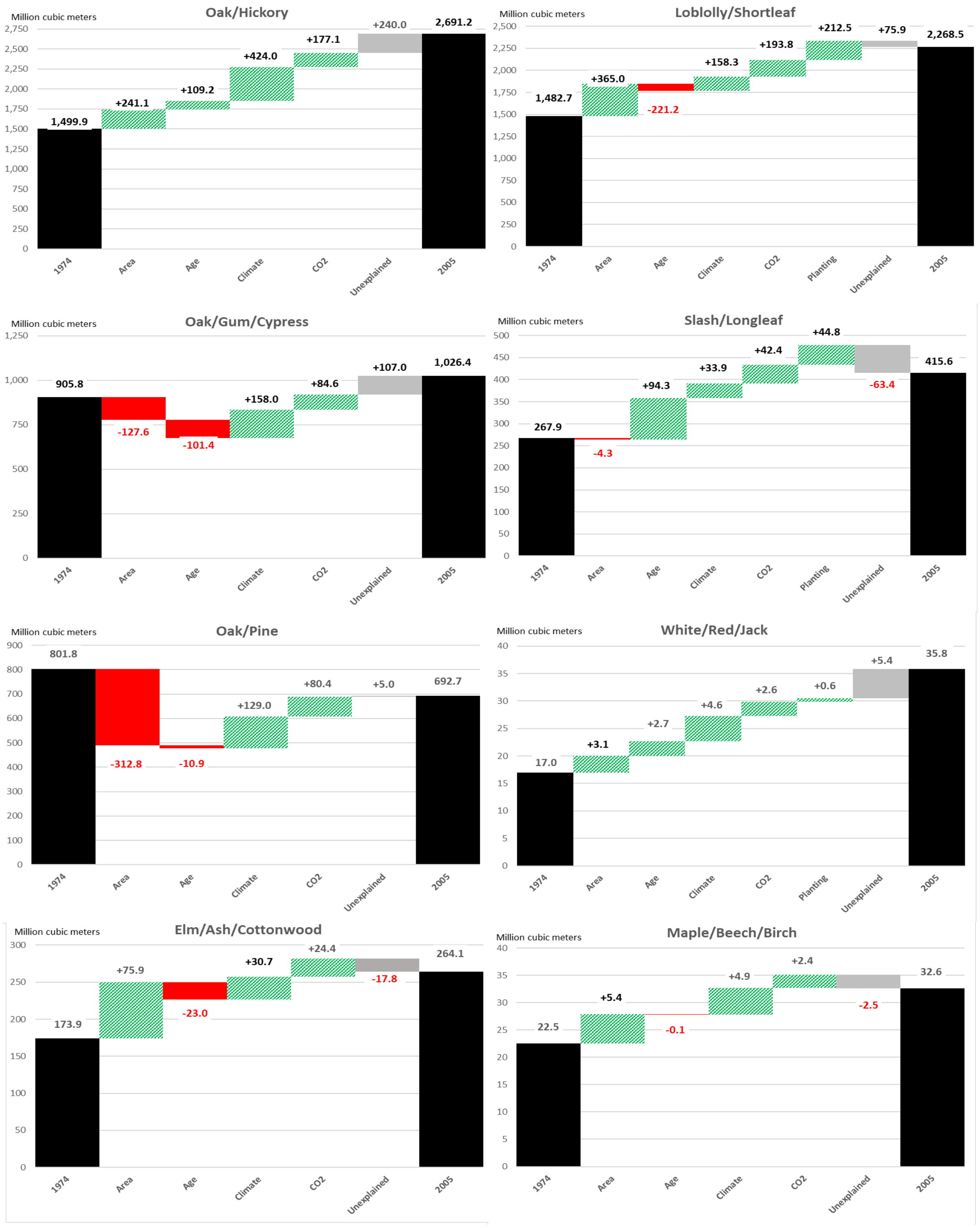


**Fig. S27. Estimated impact in 12 southern states of key drivers (area, age, climate, $CO_2$ and planting) on change in wood volume in stands aged 1 to 100 years from 1974 to 2005.** States are AL, AR, FL, GA, LA, MS, NC, OK, SC, TN, TX, & VA. Using the USFS-FIA evaluations closest to 1974 (range: 1968 to 1980) and 2005 (range: 2004 to 2010), the area column shows the impact of the change in the range of each forest group across this period. The age column shows the impact of changes in age composition from 1974 to 2005. The $CO_2$ column shows in the same way the impact of elevated $CO_2$. The planting column details the gain derived from planting on stands that were managed in 2005. The unexplained column shows the gap between our estimates and the actual change that the USFS recorded as happening across these years.

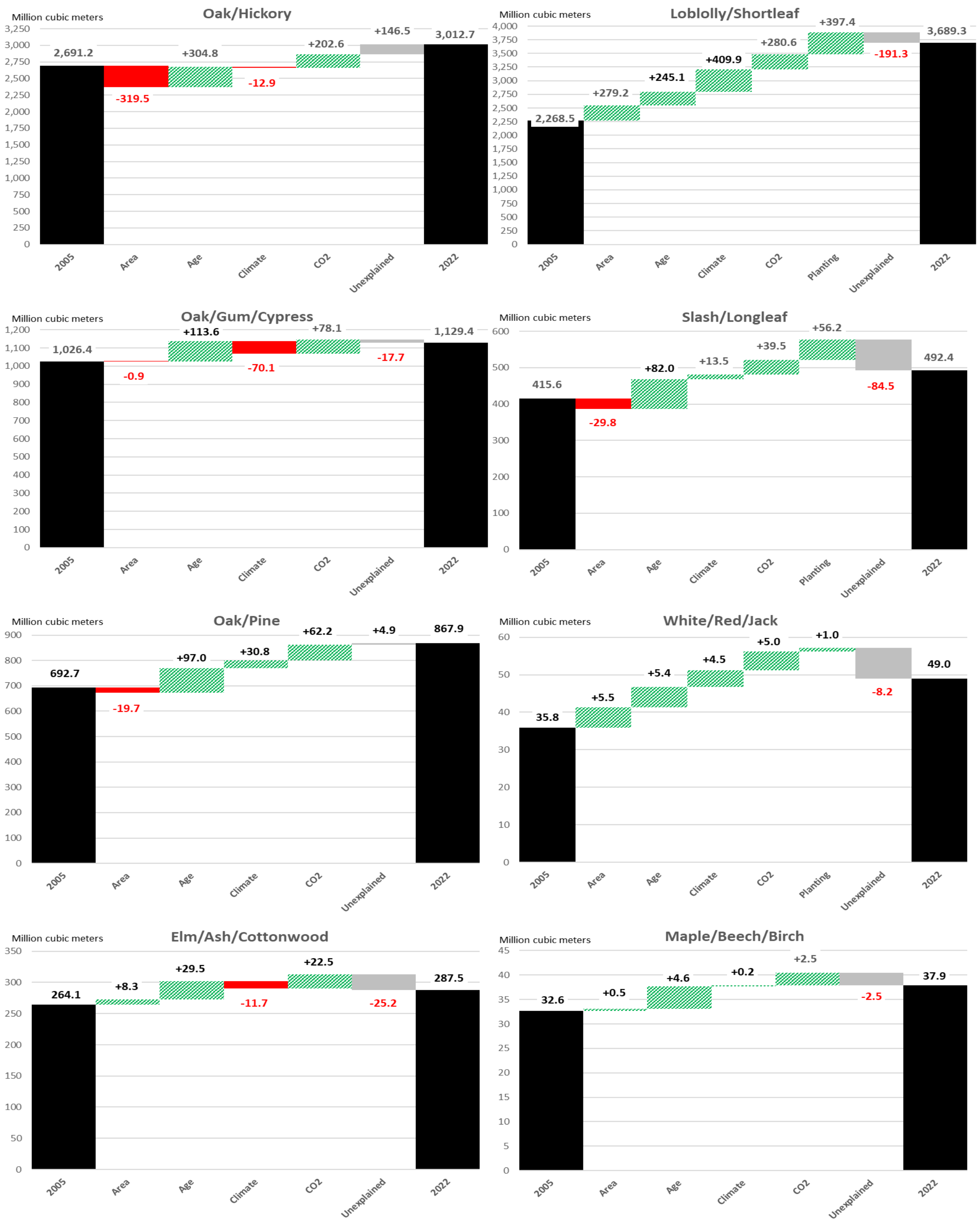


**Fig. S28. Estimated impact in 12 southern states of key drivers (area, age, climate, $CO_2$ and planting) on change in wood volume in stands aged 1 to 100 years from 2005 to 2022.** States are AL, AR, FL, GA, LA, MS, NC, OK, SC, TN, TX, & VA. Using the USFS-FIA evaluations closest to 2005 (range: 2004 to 2010) and 2022 (range: 2020 to 2022), the area column shows the impact of the change in the range of each forest group across this period. The age column shows the impact of changes in age composition from 2005 to 2022. The $CO_2$ column shows in the same way the impact of elevated $CO_2$. The planting column details the gain derived from planting on stands that were managed in 2022. The unexplained column shows the gap between our estimates and the actual change that the USFS recorded as happening across these years.

**Table S15. Volume and area in 2005 of stands aged 1 to 100 years in 21 northern states, by forest group and regeneration method.**

| | 2005 | | | | | | | | | | |
|---|---|---|---|---|---|---|---|---|---|---|---|
| | **Oak/ Pine** | **Oak/ Hickory** | **Oak/Gum/ Cypress** | **Elm/Ash/ Cottonwood** | **Maple/Beech/ /Birch** | **Aspen/ Birch** | **Spruce/ Fir** | **White/Red/ Jack** | | **Loblolly/ Shortleaf** | |
| | Naturally Regenerated | Naturally Regenerated | Naturally Regenerated | Naturally Regenerated | Naturally Regenerated | Naturally Regenerated | Naturally Regenerated | Naturally Regenerated | Planted | Naturally Regenerated | Planted |
| **Age Class** | **Volume (cubic meters)** | | | | | | | | | | |
| 0-10 years | 800,471 | 12,290,843 | 0 | 1,312,335 | 5,812,820 | 6,711,818 | 1,245,610 | 685,784 | 195,380 | 1,754 | 57,696 |
| 11-20 years | 1,987,209 | 16,399,197 | 18,243 | 3,811,355 | 10,003,943 | 11,840,764 | 6,103,007 | 1,188,894 | 3,666,406 | 308,783 | 179,140 |
| 21-30 years | 9,021,937 | 65,150,019 | 790,202 | 19,163,725 | 22,190,571 | 26,379,776 | 9,417,820 | 4,621,827 | 11,828,182 | 1,087,734 | 1,681,240 |
| 31-40 years | 17,807,666 | 208,342,108 | 2,039,692 | 46,638,003 | 66,048,439 | 57,738,358 | 17,290,870 | 16,773,137 | 29,760,464 | 6,588,146 | 3,110,025 |
| 41-50 years | 37,348,261 | 404,673,273 | 6,144,208 | 91,565,521 | 187,218,441 | 89,869,563 | 35,434,603 | 37,357,053 | 53,143,650 | 11,278,150 | 8,107,095 |
| 51-60 years | 57,963,920 | 600,763,843 | 5,811,523 | 102,543,784 | 356,151,128 | 95,325,117 | 52,174,810 | 67,492,765 | 35,744,371 | 14,489,657 | 3,331,542 |
| 61-70 years | 55,975,999 | 720,502,380 | 5,248,160 | 75,392,340 | 476,149,213 | 93,764,524 | 61,969,917 | 71,515,177 | 20,211,435 | 10,071,458 | 919,835 |
| 71-80 years | 48,469,679 | 623,136,391 | 9,872,142 | 48,492,071 | 482,135,340 | 60,836,342 | 70,334,506 | 72,234,303 | 8,119,111 | 15,018,662 | 1,876 |
| 81-90 years | 34,979,101 | 402,406,787 | 2,992,920 | 24,935,724 | 303,436,144 | 22,257,585 | 52,963,829 | 43,773,332 | 2,523,930 | 5,006,201 | 0 |
| 91-100 years | 9,758,140 | 223,621,718 | 3,426,465 | 12,387,159 | 158,764,133 | 8,409,246 | 38,094,218 | 29,101,402 | 3,760,557 | 1,856,030 | 32,869 |
| **Total** | **274,112,383** | **3,277,286,559** | **36,343,555** | **426,242,017** | **2,067,910,171** | **473,133,093** | **345,029,190** | **344,743,675** | **168,953,487** | **65,706,575** | **17,421,317** |
| **Age Class** | **Area (hectares)** | | | | | | | | | | |
| 0-10 years | 72,936 | 717,819 | 0 | 159,252 | 335,048 | 710,015 | 91,015 | 35,767 | 48,324 | 31,527 | 9,974 |
| 11-20 years | 86,759 | 771,483 | 12,654 | 216,669 | 465,890 | 831,628 | 436,646 | 57,095 | 127,532 | 13,868 | 3,456 |
| 21-30 years | 181,681 | 1,275,985 | 10,151 | 340,149 | 594,609 | 790,857 | 360,388 | 100,541 | 132,133 | 29,441 | 20,945 |
| 31-40 years | 273,105 | 2,521,124 | 26,762 | 691,585 | 957,191 | 865,005 | 379,298 | 185,626 | 184,116 | 68,308 | 22,612 |
| 41-50 years | 359,264 | 3,785,791 | 54,899 | 932,151 | 1,936,662 | 975,829 | 576,406 | 285,238 | 240,837 | 79,781 | 36,894 |
| 51-60 years | 461,730 | 4,621,383 | 51,105 | 890,090 | 2,802,948 | 868,736 | 674,838 | 372,926 | 138,188 | 109,512 | 15,930 |
| 61-70 years | 330,342 | 4,931,929 | 32,757 | 606,156 | 3,330,277 | 731,016 | 671,558 | 365,605 | 74,612 | 83,907 | 2,779 |
| 71-80 years | 303,838 | 4,007,892 | 66,896 | 364,541 | 2,996,884 | 458,187 | 691,988 | 302,312 | 30,520 | 98,973 | 77 |
| 81-90 years | 181,163 | 2,448,904 | 25,447 | 179,588 | 1,792,403 | 148,109 | 477,481 | 203,208 | 11,071 | 41,710 | 0 |
| 91-100 years | 54,276 | 1,320,786 | 16,714 | 97,062 | 919,998 | 57,529 | 336,087 | 121,306 | 11,432 | 18,305 | 511 |
| **Total** | **2,305,093** | **26,403,096** | **297,385** | **4,477,242** | **16,131,912** | **6,436,912** | **4,695,705** | **2,029,624** | **998,764** | **575,331** | **113,178** |

Data approximate 2005 stocking levels, using USFS-FIA evaluations from 2005 for 21 states: CT, DE, IA, IL, IN, KY, MA, MD, ME, MI, MN, MO, NH, NJ, NY, OH, PA, RI, VT, WI, & WV. Volume is the net merchantable bole volume of growing-stock trees (at least 12.5 cm diameter at breast height), in cubic meters, on timberland. Area is the area of timberland in hectares.

**Table S16. Volume and area in 2022 of stands aged 1 to 100 years in 21 northern states, by forest group and regeneration method.**

| 2022 | | | | | | | | | | | |
|---|---|---|---|---|---|---|---|---|---|---|---|
| | **Oak/ Pine** | **Oak/ Hickory** | **Oak/Gum/ Cypress** | **Elm/Ash/ Cottonwood** | **Maple/Beech/ /Birch** | **Aspen/ Birch** | **Spruce/ Fir** | **White/Red/ Jack** | | **Loblolly/ Shortleaf** | |
| | Naturally Regenerated | Naturally Regenerated | Naturally Regenerated | Naturally Regenerated | Naturally Regenerated | Naturally Regenerated | Naturally Regenerated | Naturally Regenerated | Planted | Naturally Regenerated | Planted |
| **Age Class** | **Volume (cubic meters)** | | | | | | | | | | |
| 0-10 years | 919,830 | 6,932,881 | 0 | 550,643 | 3,627,776 | 4,628,623 | 1,829,643 | 1,782,527 | 316,485 | 138,233 | 100,235 |
| 11-20 years | 1,385,194 | 14,706,169 | 20,522 | 3,802,656 | 6,606,004 | 11,522,082 | 1,606,261 | 3,113,502 | 3,191,121 | 1,084,209 | 2,187,545 |
| 21-30 years | 6,468,015 | 33,840,668 | 678,096 | 9,627,855 | 15,716,736 | 37,050,254 | 10,542,175 | 5,791,783 | 12,588,993 | 2,470,617 | 4,841,480 |
| 31-40 years | 8,160,795 | 78,967,447 | 1,391,528 | 22,826,187 | 39,485,211 | 64,518,189 | 34,401,158 | 8,636,141 | 26,727,783 | 3,900,872 | 8,878,234 |
| 41-50 years | 21,915,934 | 210,120,787 | 3,932,546 | 53,156,274 | 91,593,881 | 81,454,267 | 34,133,559 | 16,355,698 | 30,566,242 | 8,958,777 | 3,477,697 |
| 51-60 years | 38,777,888 | 440,768,947 | 8,162,992 | 97,125,042 | 189,166,094 | 74,934,140 | 35,825,421 | 41,501,557 | 55,952,698 | 12,408,932 | 6,699,168 |
| 61-70 years | 61,419,081 | 639,998,015 | 7,481,176 | 122,803,009 | 338,280,834 | 64,240,974 | 51,297,721 | 67,923,999 | 49,925,229 | 13,383,965 | 6,012,709 |
| 71-80 years | 71,698,116 | 783,799,617 | 12,207,257 | 111,588,163 | 514,853,048 | 58,110,202 | 61,933,142 | 96,921,919 | 36,114,847 | 14,367,244 | 3,337,720 |
| 81-90 years | 53,136,071 | 740,692,843 | 7,452,420 | 68,142,649 | 524,348,947 | 43,820,213 | 64,416,393 | 95,807,897 | 8,660,022 | 10,128,569 | 658,513 |
| 91-100 years | 37,587,492 | 534,295,348 | 5,873,774 | 39,383,643 | 363,150,594 | 22,630,323 | 63,646,365 | 70,258,480 | 3,503,310 | 4,888,090 | 0 |
| **Total** | **301,468,416** | **3,484,122,720** | **47,200,313** | **529,006,121** | **2,086,829,126** | **462,909,266** | **359,631,837** | **408,093,502** | **227,546,732** | **71,729,507** | **36,193,302** |
| **Age Class** | **Area (hectares)** | | | | | | | | | | |
| 0-10 years | 30,449 | 371,294 | 0 | 69,973 | 173,161 | 558,156 | 79,065 | 32,769 | 35,663 | 14,949 | 11,435 |
| 11-20 years | 58,654 | 618,357 | 3,658 | 143,311 | 189,109 | 628,258 | 149,105 | 74,093 | 78,968 | 33,294 | 19,570 |
| 21-30 years | 130,705 | 746,769 | 14,033 | 259,463 | 459,169 | 834,025 | 370,132 | 116,990 | 146,219 | 34,042 | 25,838 |
| 31-40 years | 136,580 | 1,209,220 | 14,664 | 359,255 | 751,056 | 829,772 | 628,469 | 116,864 | 173,690 | 31,024 | 32,194 |
| 41-50 years | 228,467 | 2,152,634 | 34,066 | 587,449 | 1,157,689 | 764,560 | 498,056 | 147,265 | 156,133 | 75,028 | 16,853 |
| 51-60 years | 319,491 | 3,532,749 | 58,320 | 915,567 | 1,767,314 | 614,192 | 523,705 | 253,099 | 216,220 | 72,364 | 21,305 |
| 61-70 years | 405,219 | 4,341,750 | 49,684 | 917,440 | 2,553,528 | 483,647 | 619,103 | 314,032 | 146,070 | 101,337 | 18,333 |
| 71-80 years | 415,212 | 4,868,250 | 64,364 | 791,338 | 3,276,716 | 435,532 | 648,061 | 406,109 | 122,766 | 74,047 | 9,036 |
| 81-90 years | 267,313 | 4,277,989 | 40,953 | 485,730 | 3,056,598 | 276,230 | 588,886 | 353,239 | 31,175 | 58,472 | 2,426 |
| 91-100 years | 170,088 | 2,881,573 | 34,723 | 274,002 | 1,991,974 | 135,301 | 503,427 | 268,093 | 10,436 | 24,557 | 0 |
| **Total** | **2,162,178** | **25,000,585** | **314,465** | **4,803,527** | **15,376,315** | **5,559,673** | **4,608,009** | **2,082,552** | **1,117,340** | **519,115** | **156,989** |

Data approximate 2022 stocking levels, using USFS-FIA evaluations closest to 2022 (range: 2020 to 2022) for 21 states: CT, DE, IA, IL, IN, KY, MA, MD, ME, MI, MN, MO, NH, NJ, NY, OH, PA, RI, VT, WI, & WV. Volume is the net merchantable bole volume of growing-stock trees (at least 12.5 cm diameter at breast height), in cubic meters, on timberland. Area is the area of timberland in hectares.

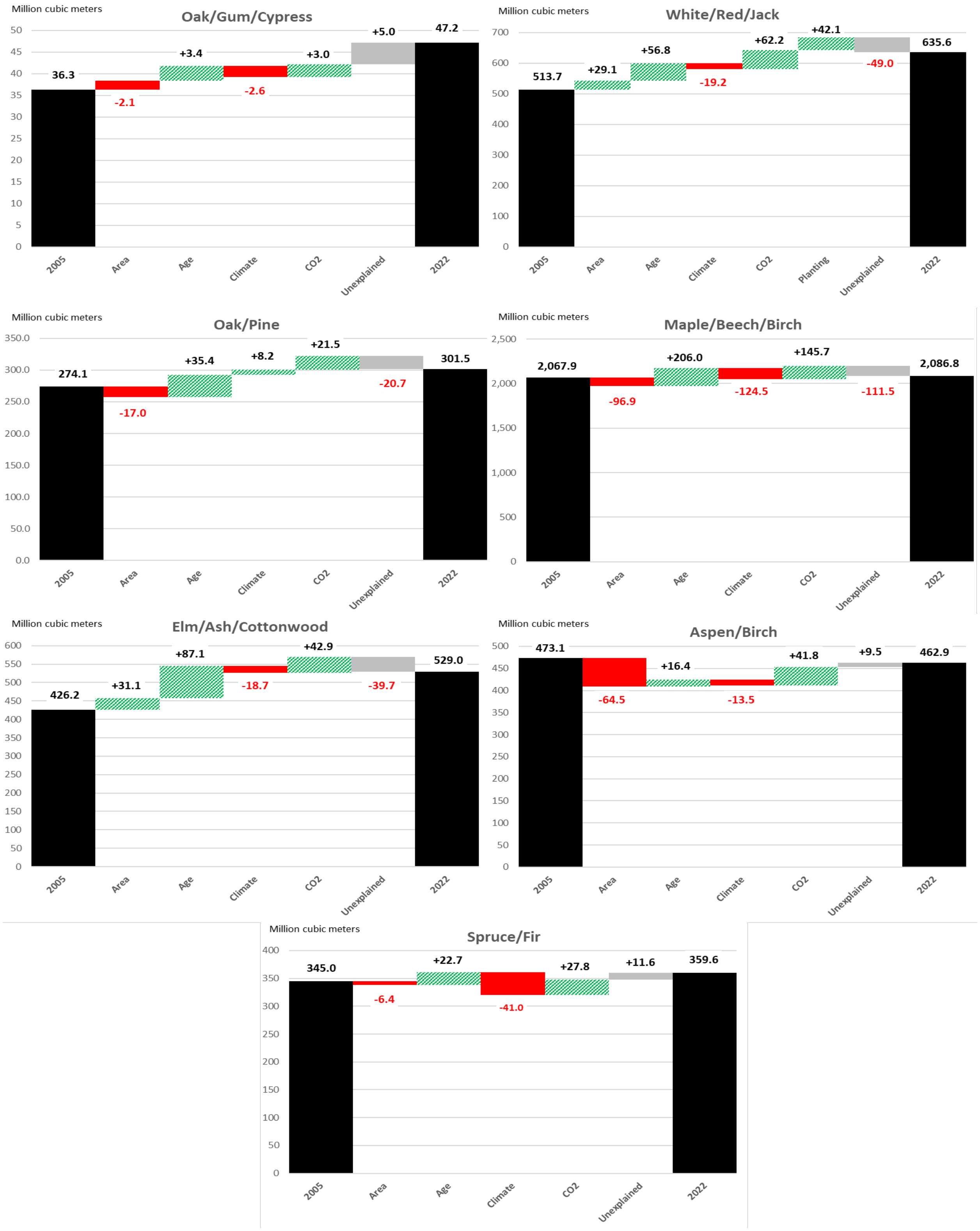


**Fig. S29. Estimated impact in 21 northern states of key drivers (area, age, climate, $CO_2$ and planting) on change in wood volume in stands aged 1 to 100 years from 2005 to 2022.** States are CT, DE, IA, IL, IN, KY, MA, MD, ME, MI, MN, MO, NH, NJ, NY, OH, PA, RI, VT, WI, & WV. Using USFS-FIA evaluations closest to 2005 (range: 2005 to 2005) and 2022 (range: 2020 to 2022), the area column shows the impact of the change in the range of each forest group and the age column shows the impact of changes in age composition from 2005 to 2022. The $CO_2$ column shows in the same way the impact of elevated $CO_2$. The planting column details the gain derived from planting on stands that were managed in 2022. The unexplained column shows the gap between our estimates and the actual change that the USFS recorded as happening across these years.

**Table S17. Volume and area in 2005 of stands aged 1 to 200 in 15 western states, by forest group and regeneration method.**

| 2005 | | | | | |
|---|---|---|---|---|---|
| | **Ponderosa Pine** | **Fir/ Spruce** | **Lodgepole pine** | **Douglas Fir** | |
| | Naturally Regenerated | Naturally Regenerated | Naturally Regenerated | Naturally Regenerated | Planted |
| **Age Class** | **Volume (cubic meters)** | | | | |
| 0-10 years | 3,855,824 | 4,951,262 | 1,909,659 | 10,467,384 | 6,520,198 |
| 11-20 years | 2,848,792 | 16,737,329 | 7,132,388 | 15,498,365 | 48,120,512 |
| 21-30 years | 4,512,024 | 9,446,492 | 6,826,360 | 28,438,677 | 144,921,695 |
| 31-40 years | 13,070,262 | 14,280,733 | 6,160,391 | 92,001,579 | 145,634,479 |
| 41-50 years | 24,729,014 | 27,608,623 | 11,404,977 | 124,324,894 | 84,083,355 |
| 51-60 years | 47,229,431 | 58,307,845 | 18,453,908 | 175,522,462 | 42,966,805 |
| 61-70 years | 81,363,594 | 95,574,539 | 35,095,286 | 191,689,714 | 29,111,261 |
| 71-80 years | 137,065,348 | 150,065,990 | 79,305,060 | 244,839,404 | 9,172,120 |
| 81-90 years | 135,349,950 | 120,928,547 | 71,216,570 | 222,714,780 | 1,939,247 |
| 91-100 years | 158,435,921 | 166,996,504 | 83,117,451 | 274,668,007 | 622,022 |
| 101-110 years | 88,538,748 | 154,898,938 | 66,460,135 | 183,437,422 | 3,194,165 |
| 111-120 years | 57,920,561 | 163,616,420 | 50,916,132 | 145,818,904 | 1,270,805 |
| 121-130 years | 60,972,050 | 129,998,368 | 41,785,386 | 141,067,509 | 897,069 |
| 131-140 years | 30,309,911 | 111,327,256 | 25,559,923 | 96,011,038 | 0 |
| 141-150 years | 36,765,005 | 138,511,989 | 29,640,559 | 119,270,974 | 816,473 |
| 151-160 years | 23,644,749 | 76,923,721 | 17,775,772 | 83,253,448 | 133,821 |
| 161-170 years | 10,667,106 | 71,457,175 | 11,738,612 | 59,533,110 | 744,531 |
| 171-180 years | 11,446,060 | 76,570,456 | 8,690,079 | 48,481,233 | 0 |
| 181-190 years | 7,049,489 | 41,353,843 | 11,359,086 | 41,835,843 | 0 |
| 191-200 years | 20,078,908 | 73,820,333 | 11,243,617 | 93,505,890 | 0 |
| **Total** | **955,852,749** | **1,703,376,363** | **595,791,350** | **2,392,380,636** | **520,148,558** |
| **Age Class** | **Area (hectares)** | | | | |
| 0-10 years | 194,961 | 204,925 | 245,621 | 374,421 | 612,060 |
| 11-20 years | 170,918 | 487,353 | 332,080 | 455,224 | 722,910 |
| 21-30 years | 123,498 | 235,652 | 178,574 | 228,336 | 754,034 |
| 31-40 years | 190,224 | 197,878 | 122,085 | 421,535 | 469,573 |
| 41-50 years | 327,331 | 203,452 | 124,935 | 524,202 | 202,550 |
| 51-60 years | 524,718 | 323,273 | 176,066 | 561,051 | 79,802 |
| 61-70 years | 871,085 | 396,604 | 237,577 | 781,819 | 48,778 |
| 71-80 years | 1,180,278 | 710,910 | 433,994 | 1,149,259 | 19,208 |
| 81-90 years | 1,084,881 | 564,556 | 402,206 | 1,081,960 | 4,810 |
| 91-100 years | 1,160,647 | 718,468 | 478,811 | 1,184,847 | 1,570 |
| 101-110 years | 655,128 | 647,884 | 338,568 | 767,471 | 4,435 |
| 111-120 years | 490,084 | 646,493 | 269,934 | 535,272 | 4,335 |
| 121-130 years | 407,893 | 512,036 | 207,187 | 460,112 | 1,589 |
| 131-140 years | 176,989 | 431,411 | 124,255 | 290,704 | 0 |
| 141-150 years | 216,049 | 504,129 | 117,173 | 315,615 | 1,423 |
| 151-160 years | 107,988 | 302,032 | 78,534 | 274,198 | 1,323 |
| 161-170 years | 79,349 | 255,771 | 46,387 | 183,260 | 854 |
| 171-180 years | 69,257 | 231,428 | 53,564 | 171,318 | 0 |
| 181-190 years | 51,264 | 160,092 | 42,305 | 156,199 | 0 |
| 191-200 years | 85,467 | 216,665 | 52,875 | 246,424 | 0 |
| **Total** | **8,168,008** | **7,951,015** | **4,062,731** | **10,163,226** | **2,929,254** |

*Note*: Data approximate 2005 stocking levels, using USFS-FIA evaluations closest to 2005 (range: 1999 to 2011) in 15 states: AZ, CA, CO, ID, KS, MT, ND, NE, NM, NV, OR, SD, UT, WA, & WY. Volume is the net merchantable bole volume of growing-stock trees (at least 12.5 cm diameter at breast height), in cubic meters, on timberland. Area is the area of timberland in hectares.

**Table S18. Volume and area in 2021 of stands aged 1 to 200 in 15 western states, by forest group and regeneration method.**

| 2021 | | | | | |
|---|---|---|---|---|---|
| | **Ponderosa Pine** | **Fir/ Spruce** | **Lodgepole pine** | **Douglas Fir** | |
| | Naturally Regenerated | Naturally Regenerated | Naturally Regenerated | Naturally Regenerated | Planted |
| **Age Class** | **Volume (cubic meters)** | | | | |
| 0-10 years | 3,535,467 | 6,985,876 | 3,226,340 | 9,281,763 | 6,155,959 |
| 11-20 years | 3,019,257 | 13,420,206 | 8,282,138 | 12,306,566 | 42,765,407 |
| 21-30 years | 3,476,969 | 10,893,200 | 8,707,563 | 16,102,571 | 142,754,288 |
| 31-40 years | 10,101,280 | 16,195,671 | 12,196,831 | 39,220,993 | 236,664,319 |
| 41-50 years | 23,197,020 | 27,468,645 | 17,255,834 | 103,292,224 | 162,950,325 |
| 51-60 years | 34,501,259 | 46,686,357 | 15,713,510 | 112,716,349 | 98,076,981 |
| 61-70 years | 52,115,900 | 68,161,596 | 19,496,897 | 165,755,779 | 48,791,315 |
| 71-80 years | 90,811,134 | 117,530,602 | 34,323,076 | 214,743,699 | 19,924,281 |
| 81-90 years | 141,772,455 | 157,306,806 | 50,726,683 | 243,527,373 | 10,758,499 |
| 91-100 years | 149,602,031 | 158,075,666 | 42,105,890 | 251,285,348 | 9,549,111 |
| 101-110 years | 139,571,834 | 196,234,266 | 43,526,815 | 249,649,378 | 2,978,179 |
| 111-120 years | 90,957,643 | 147,434,777 | 35,882,143 | 185,177,092 | 1,502,242 |
| 121-130 years | 68,370,324 | 134,047,304 | 31,345,611 | 165,048,463 | 1,027,905 |
| 131-140 years | 40,382,098 | 121,406,654 | 25,236,247 | 140,647,087 | 258,588 |
| 141-150 years | 25,243,961 | 104,093,040 | 17,295,612 | 101,640,496 | 0 |
| 151-160 years | 27,451,177 | 78,623,741 | 9,715,582 | 117,216,247 | 1,175,060 |
| 161-170 years | 15,834,499 | 71,754,512 | 5,997,559 | 91,285,072 | 762,604 |
| 171-180 years | 10,031,773 | 54,132,976 | 7,655,018 | 64,013,909 | 0 |
| 181-190 years | 10,178,125 | 65,313,256 | 5,306,698 | 66,171,537 | 1,113,237 |
| 191-200 years | 5,239,930 | 41,790,946 | 2,121,270 | 38,844,002 | 0 |
| **Total** | **945,394,136** | **1,637,556,096** | **396,117,316** | **2,387,925,947** | **787,208,298** |
| **Age Class** | **Area (hectares)** | | | | |
| 0-10 years | 156,491 | 253,933 | 324,146 | 248,928 | 532,610 |
| 11-20 years | 140,999 | 447,342 | 425,038 | 396,965 | 640,391 |
| 21-30 years | 111,588 | 278,473 | 252,535 | 252,593 | 743,364 |
| 31-40 years | 160,698 | 205,318 | 194,334 | 265,965 | 779,002 |
| 41-50 years | 253,691 | 196,785 | 195,852 | 437,685 | 394,448 |
| 51-60 years | 362,753 | 250,245 | 155,931 | 465,949 | 201,379 |
| 61-70 years | 483,509 | 361,576 | 157,277 | 612,026 | 86,349 |
| 71-80 years | 758,675 | 469,791 | 257,408 | 786,055 | 35,239 |
| 81-90 years | 1,145,725 | 685,749 | 295,070 | 1,031,502 | 26,709 |
| 91-100 years | 1,035,259 | 661,041 | 266,191 | 1,084,611 | 11,521 |
| 101-110 years | 940,580 | 779,482 | 257,593 | 949,622 | 4,123 |
| 111-120 years | 643,218 | 595,425 | 226,266 | 746,786 | 1,964 |
| 121-130 years | 442,175 | 573,926 | 198,576 | 574,956 | 3,340 |
| 131-140 years | 262,223 | 476,971 | 126,109 | 483,890 | 1,919 |
| 141-150 years | 145,431 | 391,395 | 78,178 | 333,799 | 0 |
| 151-160 years | 172,034 | 323,077 | 56,119 | 309,328 | 2,947 |
| 161-170 years | 94,665 | 242,866 | 23,545 | 258,176 | 721 |
| 171-180 years | 64,808 | 209,626 | 37,853 | 171,885 | 0 |
| 181-190 years | 53,325 | 213,993 | 35,292 | 177,845 | 781 |
| 191-200 years | 36,063 | 147,816 | 15,984 | 144,089 | 0 |
| **Total** | **7,463,909** | **7,764,830** | **3,579,298** | **9,732,653** | **3,466,808** |

*Note*: Data approximate 2021 stocking levels, using USFS-FIA evaluations closest to 2021 (range: 2020 to 2022) in 15 states: AZ, CA, CO, ID, KS, MT, ND, NE, NM, NV, OR, SD, UT, WA, & WY. Volume is the net merchantable bole volume of growing-stock trees (at least 12.5 cm diameter at breast height), in cubic meters, on timberland. Area is the area of timberland in hectares.

**Table S19. Regression model raw output by forest group, based on observations of stands aged 1 to 200, by forest group and regeneration method.**

| | Natural Log of Volume per Hectare | | | |
|---|---|---|---|---|
| Variables | Douglas-Fir | Ponderosa Pine | Fir/Spruce | Lodgepole Pine |
| Natural Log of Lifetime $CO_2$ | 1.122*10^+00*** | 8.676*10^-01*** | 1.130*10^+00*** | 1.105*10^+00*** |
| 1/Age | 7.047*10^+00*** | 6.968*10^+00*** | 2.634*10^+01*** | 1.869*10^+01*** |
| Management | 7.755*10^-02*** | | | |
| Management-Age Interaction | -5.615*10^+00*** | | | |
| Mean Spring Temperature | 6.136*10^-01*** | 4.879*10^-01*** | -3.653*10^-01*** | 4.176*10^-02 |
| Mean Spring Temperature Squared | -6.764*10^-02*** | -1.617*10^-02 | -5.030*10^-02*** | -2.341*10^-02 |
| Mean Spring Temperature Cubed | 3.242*10^-03*** | -7.474*10^-04 | 6.874*10^-03*** | 5.633*10^-03*** |
| Mean Summer Temperature | -9.506*10^-01 | -2.604*10^+00*** | 1.603*10^+00*** | 7.716*10^-01 |
| Mean Summer Temperature Squared | 3.774*10^-02 | 1.516*10^-01*** | -8.979*10^-02*** | -2.310*10^-03 |
| Mean Summer Temperature Cubed | -3.058*10^-04 | -2.806*10^-03*** | 1.853*10^-03*** | -8.957*10^-04 |
| Mean Fall Temperature | 3.542*10^-01* | 2.332*10^-01 | 1.108*10^+00*** | -5.255*10^-01** |
| Mean Fall Temperature Squared | -6.513*10^-03 | -5.096*10^-02* | -1.895*10^-03 | 1.577*10^-01*** |
| Mean Fall Temperature Cubed | -8.695*10^-04 | 2.641*10^-03** | -2.863*10^-03*** | -9.896*10^-03*** |
| Mean Winter Temperature | -1.227*10^-01*** | -1.562*10^-01*** | -5.848*10^-01*** | -3.874*10^-01*** |
| Mean Winter Temperature Squared | 3.270*10^-02*** | 5.101*10^-03 | 1.872*10^-02** | 7.949*10^-02*** |
| Mean Winter Temperature Cubed | 2.993*10^-03*** | 5.666*10^-04 | 1.154*10^-03* | 5.572*10^-03*** |
| Mean Spring Precipitation | -2.891*10^-02*** | -1.368*10^-02* | -2.608*10^-02*** | -4.563*10^-02*** |
| Mean Spring Precipitation Squared | 1.225*10^-04*** | 1.263*10^-04 | 4.60*10^-05 | 2.531*10^-04*** |
| Mean Spring Precipitation Cubed | -1.48*10^-07*** | -7.66*10^-07** | 1.28*10^-08 | -4.78*10^-07* |
| Mean Summer Precipitation | 8.514*10^-02*** | 8.577*10^-02*** | -5.188*10^-03 | -4.210*10^-02** |
| Mean Summer Precipitation Squared | -6.825*10^-04*** | -1.116*10^-03*** | 4.391*10^-04* | 8.948*10^-04*** |
| Mean Summer Precipitation Cubed | 1.63*10^-06*** | 7.00*10^-06*** | -2.59*10^-06* | -5.03*10^-06** |
| Mean Fall Precipitation | -1.282*10^-02*** | 2.989*10^-02*** | -3.563*10^-02*** | 5.240*10^-03 |
| Mean Fall Precipitation Squared | 3.60*10^-07 | -2.271*10^-04*** | 1.339*10^-04*** | -2.93*10^-05 |
| Mean Fall Precipitation Cubed | 1.06*10^-08 | 5.74*10^-07* | -2.50*10^-07*** | -6.54*10^-08 |
| Mean Winter Precipitation | 3.425*10^-02*** | -1.642*10^-02*** | 4.188*10^-02*** | 1.556*10^-02*** |
| Mean Winter Precipitation Squared | -7.36*10^-05*** | 7.36*10^-05*** | -1.583*10^-04*** | 3.13*10^-06 |
| Mean Winter Precipitation Cubed | 5.22*10^-08*** | -7.77*10^-08 | 1.97*10^-07*** | -8.40*10^-08* |
| Spring Temperature - Latitude Interaction | -1.076*10^+00*** | -1.533*10^-01 | -5.572*10^-01** | -1.464*10^+00*** |
| Summer Temperature - Latitude Interaction | 3.609*10^-01*** | -3.710*10^-01** | 1.046*10^+00*** | 1.302*10^-01 |
| Fall Temperature - Latitude Interaction | 5.138*10^-01*** | 1.088*10^+00*** | -2.811*10^+00*** | 4.542*10^-01 |
| Winter Temperature - Latitude Interaction | -1.002*10^-01 | -1.524*10^-01 | 1.137*10^+00*** | 6.131*10^-01*** |
| Spring Precipitation - Latitude Interaction | -4.112*10^-03* | 2.658*10^-02*** | 1.629*10^-01*** | 1.200*10^-02 |
| Summer Precipitation - Latitude Interaction | -5.412*10^-02*** | -7.465*10^-02*** | -2.978*10^-01*** | -1.004*10^-01*** |
| Fall Precipitation - Latitude Interaction | 1.679*10^-02*** | -8.044*10^-02*** | 8.303*10^-02*** | 6.792*10^-02*** |
| Winter Precipitation - Latitude Interaction | -7.727*10^-03*** | 2.208*10^-02** | -1.476*10^-01*** | -6.301*10^-02*** |
| Site Class 1 | 6.227*10^-01*** | 1.183*10^+00** | 1.319*10^+00*** | -1.366*10^+00*** |
| Site Class 2 | 9.067*10^-01*** | 7.427*10^-01*** | 7.686*10^-01*** | 5.294*10^-01** |
| Site Class 3 | 7.444*10^-01*** | 5.848*10^-01*** | 7.060*10^-01*** | 3.001*10^-01*** |
| Site Class 4 | 5.469*10^-01*** | 5.286*10^-01*** | 5.363*10^-01*** | 5.486*10^-01*** |
| Site Class 5 | 3.606*10^-01*** | 2.880*10^-01*** | 2.656*10^-01*** | 3.190*10^-01*** |
| Site Class 6 | - | - | - | - |
| Time Dummy (2016 to 2023) | -1.947*10^-02 | -5.929*10^-02*** | -2.824*10^-02*** | -3.069*10^-02* |
| Time Dummy (2006 to 2015) | -4.055*10^-02* | -2.215*10^-02*** | -3.984*10^-02*** | -1.391*10^-02 |
| Time Dummy (Pre 2006) | - | - | - | - |
| Elevation | -1.93*10^-07 | 4.68*10^-05*** | -6.27*10^-05*** | 9.94*10^-06 |
| Slope | -4.791*10^-04*** | -4.332*10^-04** | -7.843*10^-04*** | 8.839*10^-04** |
| Disturbances | 1.155*10^-01*** | 7.444*10^-02*** | 1.838*10^-01*** | 2.312*10^-01*** |
| Interventions | -1.294*10^-01*** | | | |
| Xeric Physiography | -1.062*10^-01*** | -8.828*10^-02*** | -1.639*10^-01*** | -1.487*10^-01*** |
| Hydric Physiography | 6.854*10^-02 | 2.570*10^-01*** | -1.442*10^-01*** | -1.307*10^-01 |
| Mesic Physiography | - | - | - | - |
| North-facing Aspect | -2.053*10^-02*** | 5.106*10^-02*** | 1.059*10^-02 | -5.415*10^-02*** |
| Null Aspect | 5.063*10^-03 | 8.831*10^-03 | 4.446*10^-02*** | -1.262*10^-01*** |
| South-facing Aspect | - | - | - | - |
| Private Land Ownership | -1.677*10^-01*** | -9.993*10^-02*** | -2.037*10^-01*** | -1.102*10^-01*** |
| Latitude | 4.491*10^-02*** | 1.713*10^-02* | -8.071*10^-02*** | 6.343*10^-02*** |
| Longitude | -4.623*10^-02*** | 2.093*10^-02** | -7.023*10^-02*** | 9.668*10^-03 |
| Stocking Density 1 | 2.043*10^+00*** | 2.358*10^+00*** | 2.145*10^+00*** | 1.426*10^+00*** |
| Stocking Density 2 | 1.700*10^+00*** | 1.939*10^+00*** | 1.895*10^+00*** | 1.491*10^+00*** |
| Stocking Density 3 | 1.353*10^+00*** | 1.505*10^+00*** | 1.570*10^+00*** | 1.403*10^+00*** |
| Stocking Density 4 | 8.009*10^-01*** | 8.483*10^-01*** | 1.075*10^+00*** | 1.093*10^+00*** |
| Stocking Density 5 | - | - | - | - |
| Constant | -1.474*10^+01*** | 5.237*10^+00 | -2.955*10^+01*** | -1.858*10^+01*** |
| Observations | 53,982 | 33,206 | 44,759 | 21,089 |
| R-squared | 0.721 | 0.671 | 0.629 | 0.537 |

Note: All regressions employed county fixed effects. ***p<0.01//**p<0.05//*p<0.10

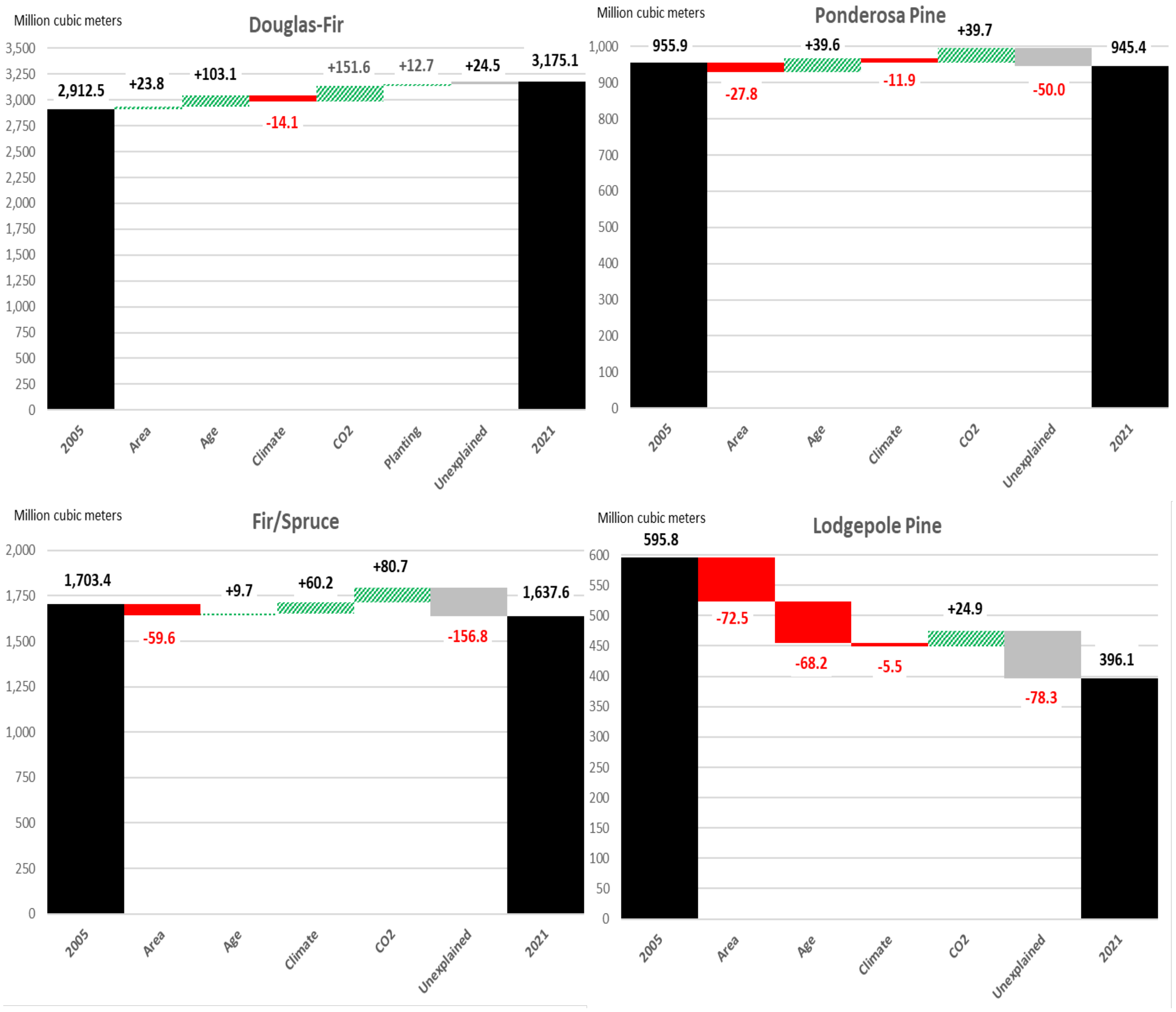


**Fig. S30. Estimated impact in 15 western states of key drivers (area, age, climate, $CO_2$ and planting) on change in wood volume in stands aged 1 to 200 years from 2005 to 2021.** States are: AZ, CA, CO, ID, KS, MT, ND, NE, NM, NV, OR, SD, UT, WA, & WY. Using USFS-FIA evaluations closest to 2005 (range: 1999 to 2011) and 2021 (range: 2020 to 2022), the area column shows the impact of the change in the range of each forest group and the age column shows the impact of changes in age composition from 2005 to 2021. The combined impact of these drivers shows the hypothetical 2021 forest without any changes due to changes in climate or $CO_2$. This hypothetical forest is then used to show the impact of the changes in climate given the age-decile-specific lifetime climate profiles of 2005 & 2021. The $CO_2$ column shows in the same way the impact of elevated $CO_2$. The unexplained column shows the gap between our estimates and the actual change that the USFS recorded as happening across these years.

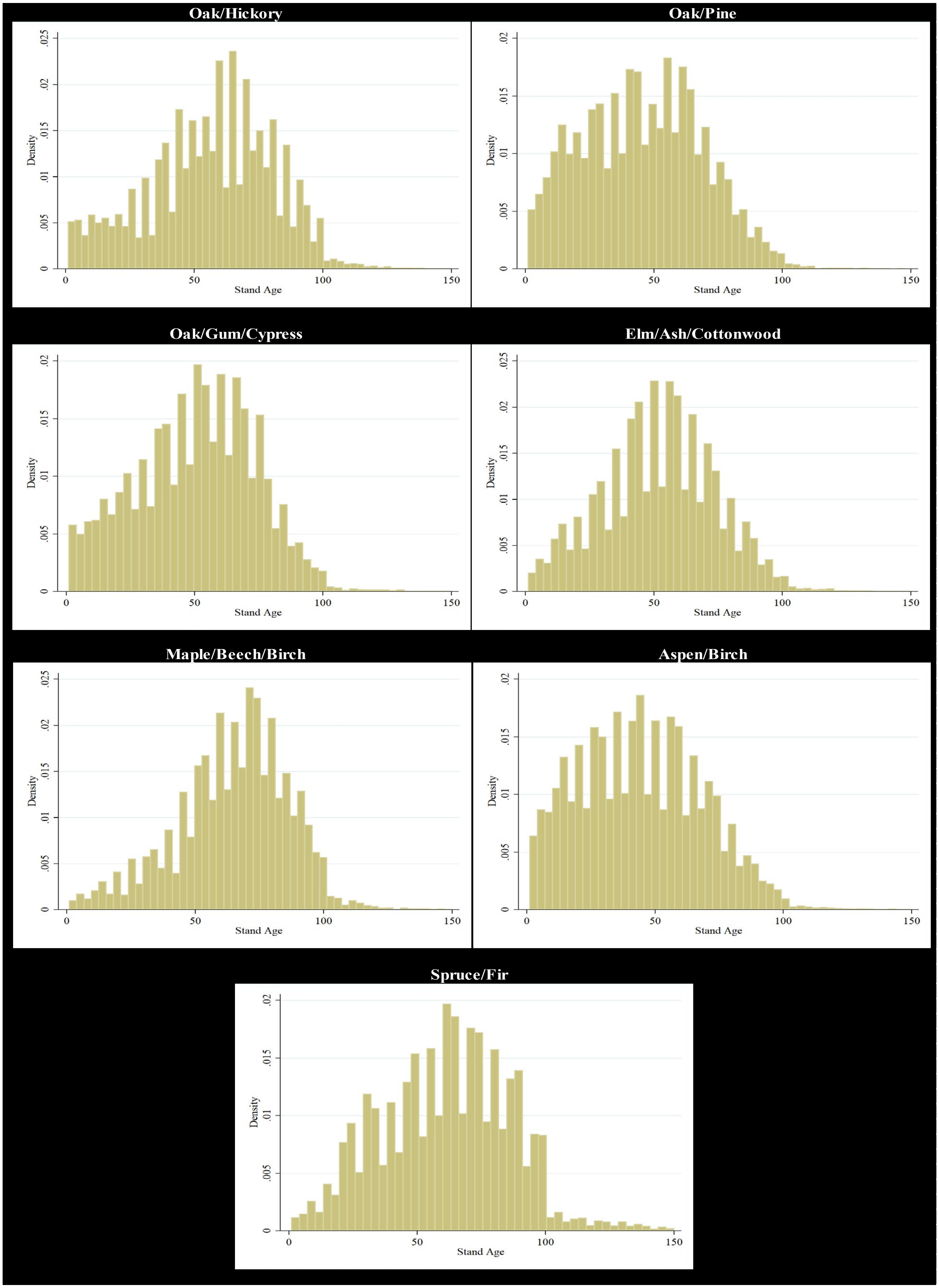


**Fig. S31. Histograms of naturally regenerated plots by age and forest group.**

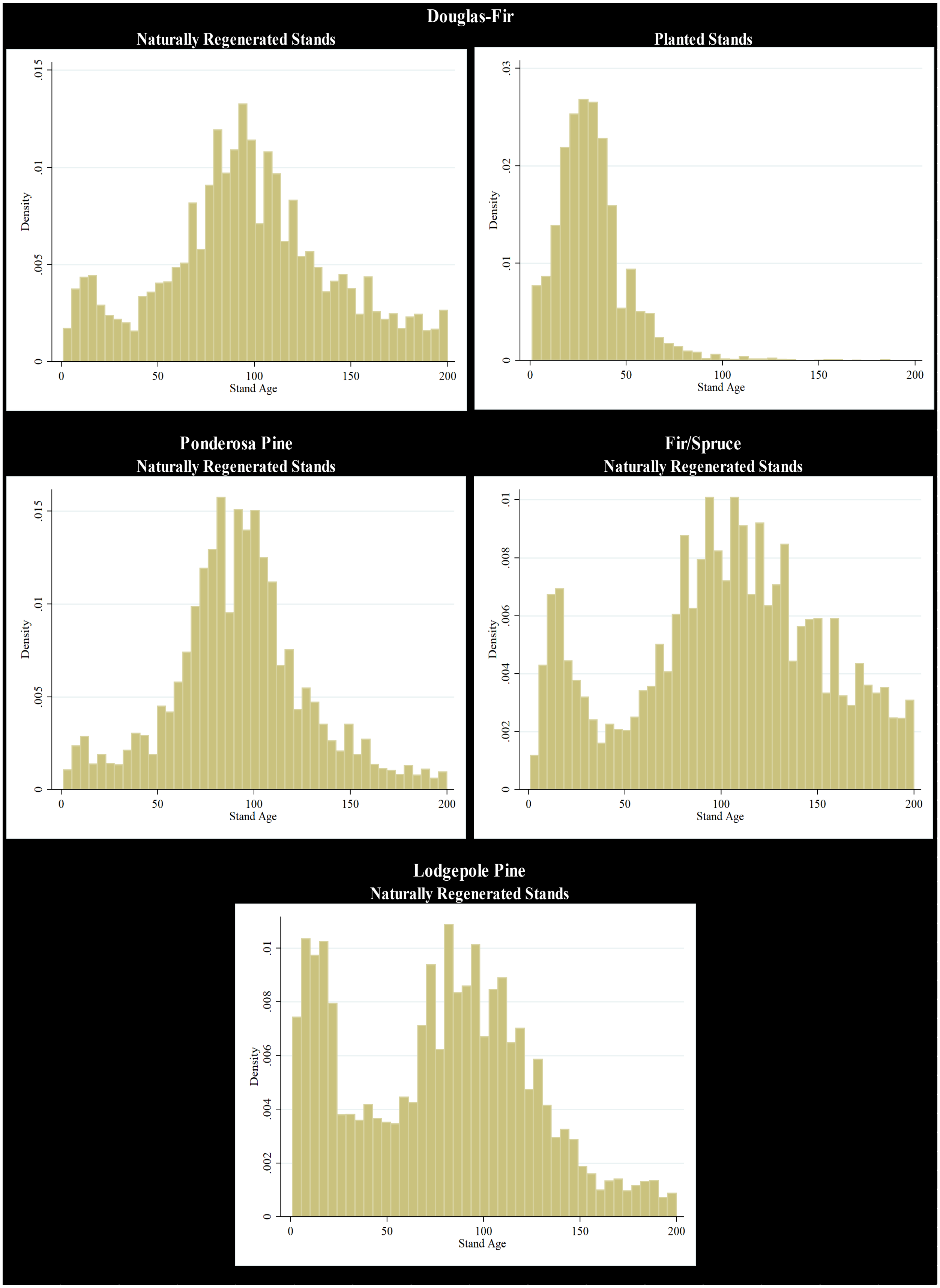


**Fig. S32. Histograms of plots by age, regeneration method, and forest group.**

**Table S20. Key USFS-FIA variables with definitions.**

| | **USFS-FIA Variables** *Burrill et al., 2024* |
|---|---|
| ***Natural Log of Volume per Hectare*** | Variable is a logarithmic transformation of the average net volume of wood in cubic meters per hectare in the central stem of trees. These data are derived from breast-height volume measurements of sample trees that are transformed using techniques that rely on the USFS' sampling design to enable estimation of the theoretical number of trees and therefore volume per hectare on each plot. |
| **Natural Log of Lifetime $CO_2$ Exposure** | Variable is a logarithmic transformation of the sum of yearly atmospheric $CO_2$ exposure over the lifetime of the stand. The $CO_2$ values are those for the years the stand lived based on the age recorded by the USFS. |
| **1/Stand Age** | Variable is the inverse of the field-recorded stand age. |
| **Regrowth Method** | Dummy variable that is equal to 1 if the stand was artificially regenerated. "An artificially regenerated stand is established by planting or artificial seeding." |
| **Site Class** | Variable based on "a classification of forest land in terms of inherent capacity to grow crops of industrial wood. Identifies the potential growth in cubic feet/acre/year and is based on the culmination of mean annual increment of fully stocked natural stands."<br>Class 1: >15.7 cubic meters/hectare/year<br>Class 2: 11.5-15.7 cubic meters/hectare/year<br>Class 3: 8.4-11.4 cubic meters/hectare/year<br>Class 4: 5.9-8.3 cubic meters/hectare/year<br>Class 5: 3.5-5.8 cubic meters/hectare/year<br>Class 6: 1.4-3.4 cubic meters/hectare/year<br>One issue that was encountered was the lack of data for land of the most productive types. This limitation means that our findings are most robust for Class 3 to 6 forest sites. |
| **Elevation** | Variable for the distance above sea level a plot is located. |
| **Aspect** | Variable included in analysis due to relevance identified by Måren et al. (2015) and identifies "the direction of the slope, to the nearest degree. North is recorded as 360. When slope is <5°, there is no aspect and this item is set to zero." Plots with an aspect between 87.5° and 92.5° and between 267.5° and 272.5° were dropped from the study to ensure that the north and south groups had a more than nominal difference. Then, the variable was formulated as a series of dummy variables for north-facing, south-facing and non-sloped plots. |
| **Physiographic Class** | Variable depicts "the general effect of land form, topographical position, and soil on moisture available to trees." There are three major types of sites: 1) Xeric sites that are normally low or deficient in available moisture, 2) Mesic sites that normally have adequate available moisture, and 3) Hydric sites that normally have abundant or overabundant moisture all year. As xeric hydrologic conditions have been found to limit growth (Newingham et al., 2013), physiographic conditions were controlled for through the use of three dummy variables for xeric, mesic and hydric soils. |
| **Slope** | The angle of slope, in percent, of the condition. Valid values are 000 through 155 and were measured by the USFS by sighting along the average incline or decline of the condition. |
| **Ownership** | Variable for the class in which the landowner belongs.<br>Public category includes:<br>11) National Forest; 12) National Grassland and/or Prairie<br>13) Other Forest Service Land; 21) National Park Service<br>22) Bureau of Land Management; 23) Fish and Wildlife Service<br>24) Departments of Defense/Energy; 25) Other Federal<br>31) State including State universities; 32) Local including water authorities<br>33) Other non-federal public<br>Private category references:<br>46) Undifferentiated private and Native American |
| **Stocking Density** | Variable indicating how densely stocked are the trees on the plot. 1) Overstocked (100+%) / 2) Fully Stocked (60-99%) / 3) Medium Stocked (35-59%) / 4) Poorly Stocked (10-34%) / 5) Non-stocked (0-9%) |
| **Disturbances** | Variable "indicating the kind of disturbance that has occurred since the last measurement or within the last 5 years for new plots. The area affected by the disturbance must be at least 1 acre in size. A significant level of disturbance (mortality or damage to 25% of the trees in the condition) is required." |
| **Latitude** | Variable indicates "the approximate latitude of the plot". |
| **Longitude** | Variable indicates "the approximate longitude of the plot". |

## SI References


1. E.A. Burrill *et al*., 2024. The Forest Inventory and Analysis Database, version 9.2, USFS (2024); https://www.fs.usda.gov/research/products/dataandtools/datasets/ fia-datamart.
2. PRISM Climate Group, PRISM Climate Data, Oregon State University (2024); http://prism. oregonstate.edu.
3. D.W. McKenney, J.H. Pedlar, K. Lawrence, K. Campbell, M.F. Hutchinson, Potential impacts of climate change on the distribution of North American trees. *BioScience* **57** (11), 939-948 (2007).
4. D.W. McKenney, J.H. Pedlar, R.B. Rood, D. Price, Revisiting projected shifts in the climate envelopes of North American trees using updated general circulation models. *Glob. Chang. Biol.* **17** (8), 2720-2730 (2011).
5. S. Inoue, Q. Dang, R. Man, B. Tedla, Photoperiod and CO2 elevation influence morphological and physiological responses to drought in trembling aspen: implications to climate change-induced migration. *Tree Physiol.* **40** (7), 917-927 (2020).
6. J. Fuhrer, Agroecosystem responses to combinations of elevated CO2, ozone, and global climate change. *Agriculture, Ecosystems & Environment* **97** (1-3), 1-20 (2003).
7. I. Chuine, A united model for budburst of trees. *Journal of Theoretical Biology* **2007**, 337-347 (2000).
8. O.M. Heide, Daylength and thermal time responses of budburst during dormancy release in some northern deciduous trees. *Physiologia Plantarum* **88** (4), 531-540 (1993).
9. D.F.B. Flynn, E.M. Wolkovich, Temperature and photoperiod drive spring phenology across all species in a temperate forest community. *New Phytol.* **219** (4), 1353-1362 (2018).
10. C.D. Keeling *et al*., Atmospheric carbon dioxide variations at Mauna Loa observatory, Hawaii. *Tellus* **28** (6), 538-551 (1976).
11. K.W. Thoning, P.P. Tans, W.D. Komhyr, Atmospheric carbon dioxide at Mauna Loa Observatory: 2. Analysis of the NOAA GMCC data, 1974–1985. *J. Geophys. Res. Atmos.* **94** (D6), 8549-8565 (1989).
12. NCEI, NOAA, NOAA National Centers for Environmental Information. (2020).
13. I.E. Måren, S. Karki, C. Prajapati, R.K. Yadav, B.B. Shrestha, Facing north or south: Does slope aspect impact forest stand characteristics and soil properties in a semiarid trans-Himalayan valley? *Journal of Arid Environments* **121**, 112-123 (2015).
14. B.A. Newingham *et al*., No cumulative effect of 10 years of elevated [$CO_2$] on perennial plant biomass components in the Mojave Desert. *Glob. Chang. Biol.* **19** (7), 2168-2181 (2013).
15. N. Duan, Smearing estimate: a nonparametric retransformation method. *Journal of the American Statistical Association* **78** (383), 605-610 (1983).
16. R.A. Birdsey, Carbon storage and accumulation in United States forest ecosystems. US Department of Agriculture, Forest Service **59**, (1992).
17. J.E. Smith, L.S. Heath, J.C. Jenkins, Forest volume-to-biomass models and estimates of mass for live and standing dead trees of US forests. US Department of Agriculture, Forest Service, Northeastern Research Station 298, (2003).
18. A. Noormets *et al*., The role of harvest residue in rotation cycle carbon balance in loblolly pine plantations. Respiration partitioning approach. *Glob. Chang. Biol.* **18** (10), 3186-3201 (2012).
19. C.A. Maier, T.J. Albaugh, H.L. Allen, P.M. Dougherty, Respiratory carbon use and carbon storage in mid-rotation loblolly pine (Pinus taeda L.) plantations: the effect of site resources on the stand carbon balance. *Glob. Chang. Biol.* **10** (8), 1335-1350 (2004).
20. K.R. Love-Myers, A. Clark III, L.R. Schimleck, E.J. Jokela, R.F. Daniels, Specific gravity responses of slash and loblolly pine following mid-rotation fertilization. *For. Ecol. Manage.*

**257** (12), 2342-2349 (2009).
21. R.S. Seymour, D.M. Smith, A new stocking guide formulation applied to eastern white pine. *For. Sci.* 33 (2), 469-484 (1987).
22. J.W. Benzie, Manager's handbook for red pine in the north-central states. *General Technical Report NC-33. St. Paul, MN: US Dept. of Agriculture, Forest Service, North Central Forest Experiment Station* 33 (1977).
23. S. Brais, P. David, R. Ouimet, Impacts of wild fire severity and salvage harvesting on the nutrient balance of jack pine and black spruce boreal stands. *For. Ecol. Manage.* **137** (1-3), 231-243 (2000).
24. USDA Forest Service, Forest Inventory EVALIDator web-application, version 2.1.2. (2024); https://apps.fs.usda.gov/fiadb-api/evalidator.
